# Genome-wide hydroxymethylcytosine pattern changes in response to oxidative stress

**DOI:** 10.1038/srep12714

**Published:** 2015-08-04

**Authors:** Benjamin Delatte, Jana Jeschke, Matthieu Defrance, Martin Bachman, Catherine Creppe, Emilie Calonne, Martin Bizet, Rachel Deplus, Laura Marroquí, Myriam Libin, Mirunalini Ravichandran, Françoise Mascart, Decio L. Eizirik, Adele Murrell, Tomasz P. Jurkowski, François Fuks

**Affiliations:** 1 Laboratory of Cancer Epigenetics, Faculty of Medicine, ULB, 1070 Brussels, Belgium; 2 CRUK Cambridge Institute, University of Cambridge, Cambridge CB2 0RE, United Kingdom; 3 ULB, Center for Diabetes Research, Faculty of Medicine, ULB, 1070 Brussels, Belgium.; 4 Laboratory of Vaccinology and Mucosal Immunity, Faculty of Medicine, ULB, 1070 Brussels, Belgium.; 5 Institute of Biochemistry, Stuttgart University, 70569 Stuttgart, Germany

## Abstract

**The TET enzymes convert methylcytosine to the newly discovered base hydroxymethylcytosine. While recent reports suggest that TETs may play a role in response to oxidative stress, this role remains uncertain, and results lack** ***in vivo*** **models. Here we show a global decrease of hydroxymethylcytosine in cells treated with buthionine sulfoximine, and in mice depleted for the major antioxidant enzymes** ***GPx*****1 and 2. Furthermore, genome-wide profiling revealed differentially hydroxymethylated regions in coding genes, and intriguingly in microRNA genes, both involved in response to oxidative stress. These results thus suggest a profound effect of** ***in vivo*** **oxidative stress on the global hydroxymethylome.**

In 2009, Anjana Rao’s laboratory showed that the ten eleven translocation (TET) enzymes (TET1, TET2, TET3) oxidize methylcytosine (mC) to hydroxymethylcytosine (hmC)^1^. In this reaction, the TET proteins use Fe^2+^, O_2_ and α-ketoglutarate as co-substrates, the latter being converted into succinate during Krebs cycle^1^. The Krebs cycle determines the redox state of cells, and a defective Krebs cycle has been linked to elevated levels of cellular oxidative stress^2^. Oxidative stress is intimately involved in chronic inflammation, which in turn is a mediator in the pathology of diseases such as cancer or neurodegenerative disorders^3^,^4^.

Recently, two publications have described the effects of quinones on the TET proteins. The authors found a global increase in genomic hydroxymethylcytosine in response to quinone treatment^5^,^6^. Quinones are toxic, redox-active cyclic compounds that can induce oxidative stress; however, they are also able to be strong reducers as exemplified by the antioxidant effect of hydroquinone against superoxide radical^7^. Quinones also act as “Michael acceptors” and covalently bind to cellular nucleophiles to form DNA/RNA and protein adducts^8^. This makes it hard to determine whether the hmC increase observed upon treatment with quinones is due to oxidative stress or has another explanation. Additionally, Scola *et al.* have observed a small increase in genomic hmC in rotenone-treated cortical neurons, yet the doses used in this study were not tested to produce oxidative stress^9^. Importantly, none of the above studies exploited *in vivo* models of oxidative stress, such as *GPx* or *Sod* knockout mice^10^.

Despite the above-mentioned advances, the impact of oxidative stress on TET-mediated hydroxymethylation is therefore unclear. We have addressed this issue using two experimental models, namely SY5Y neuroblastoma cells treated with buthionine sulfoximine (BSO), one of the most commonly used drug to induce oxidative stress, and Glutathione peroxidase 1 and 2 (*GPx*1/2) double-knockout mice. Our results show, for the first time, that both *exogenous* and *in vivo* oxidative assaults deeply modify the hydroxymethylome. Of particular interest, we unveiled an unexpected link between oxidative-stress-induced hydroxymethylation pattern changes, a set of microRNAs, and oxidative-stress-related genes.

## Results

### BSO-treated SY5Y cells and *GPx*1/2 double-knockout mice have a decreased level of hydroxymethylcytosine

**Figure 1:**
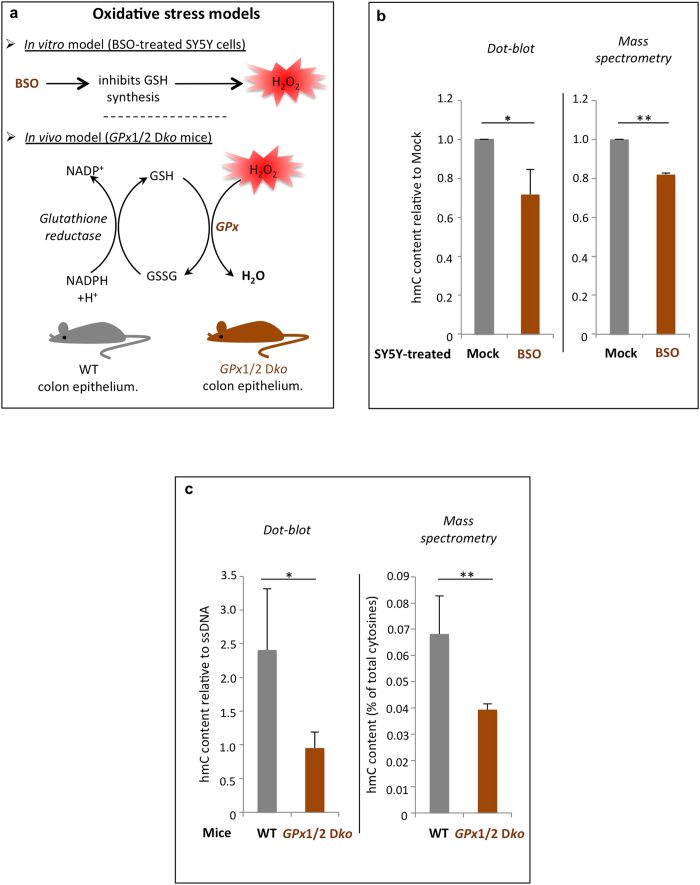
Oxidative stress reduces the global hydroxymethylcytosine level. (**A**) Scheme representing the drug used to induce oxidative stress in SY5Y neuroblastoma cells, and a description of the mouse model used in this study (**B**) Hydroxymethylcytosine quantification by dot blot analysis (left) and mass spectrometry (right). SY5Y cells were treated for 48 h with 500 μM BSO and then the global hmC content was evaluated on extracted genomic DNA. The data represent the amount of hmC normalized with respect to single-stranded DNA (dot blot) and relative to mock-treated cells. Significant differences (Student *t*-test) are indicated by * (p-value ≤ 0.05) and ** (p-value ≤ 0.01). Data represent means ± standard deviations of at least 3 independent experiments. (**C**) Hydroxymethylcytosine quantification by dot blot analysis (left) and mass spectrometry (right). Quantifications were performed on 5 wild-type (wt) and 5 double-knockout (*GPx*1/2 D*ko*) mouse colon epithelial cells, and the signals were normalized with respect to single-stranded DNA (dot blot). Mass spectrometry data shows hmC levels as a percentage of total cytosines. Significant differences (Student *t*-test) are indicated by * (p-value ≤ 0.05) and ** (p-value ≤ 0.01). See also supplementary Fig. S1.

To see whether hmC levels are affected in cells exposed to exogenous oxidative stress, we treated SY5Y cells with BSO and quantified hmC by dot-blot experiments and mass spectrometry (LC/MS/MS) (Fig. 1A,B and supplementary Fig. S1C). SY5Y is a human cell line commonly used as a model for studying oxidative stress and oxidative-stress-related diseases such as Alzheimer’s and Parkinson’s^11^,^12^. Additionally, neuroblastomas are derived from neural crest cells, and neuronal cell types have high levels of hmC^13^, making those cells a good model for our study. BSO inhibits the synthesis of the antioxidant glutathione (GSH) and causes oxidative stress by generating H_2_O_2_ (Fig. 1A and supplementary Fig. S1B)^14^. As shown in Fig. 1B, BSO-treated cells were found to have a significant decrease of the global hmC level. Further, this observation appeared not to be attributable to an increased apoptosis or necrosis, as BSO treatment did not increase the proportion of apoptotic or necrotic cells as compared to mock-treated cells (supplementary Fig. S1A).

Several studies have shown that BSO-treated SY5Y cells display a significant decrease in proliferation in response to oxidative stress^15^,^16^. We therefore measured proliferation with the xCELLigence technology, which records changes in conductivity that are proportional to the number of cells attached to the incubation chamber. Upon treatment with BSO, the proliferation decrease was greater in TET1-knockdown cells than in TET2-knockdown cells or in control cells (supplementary Fig. S1D). It is worth noting that TET3 does not seem to be expressed in SY5Y cells, and that the effects observed here cannot be attributed to an off-target effect of TET1-RNAi on TET2 (supplementary Fig. S1D, and data not shown).

To confirm and extend the above findings, we used an *in vivo* model of oxidative stress: double-knockout mice lacking the genes encoding glutathione peroxidases 1 and 2 (called hereafter *GPx*1/2 D*ko*). The GPx enzymes are known to reduce H_2_O_2_ and are viewed as the major cellular antioxidant enzymes, especially in intestinal epithelial cells where reactive oxygen species accumulate upon their depletion (Fig. 1A)^10^. Dot-blot and mass spectrometry experiments were performed on *GPx*1/2 D*ko* and wild-type (wt) colon epithelia, and in agreement with our data on SY5Y cells, the global hmC level was found to be lower in *GPx*1/2 D*ko* mice than in their wt counterparts (Fig. 1C).

Our results thus suggest that hydroxymethylcytosine levels are decreased upon *exogenous* and *in vivo* oxidative assaults, and that SY5Y cells with reduced TET1 expression are more sensitive to oxidative stress.

### hmC deep-sequencing profiles of BSO-treated SY5Y cells highlight pathways involved in the oxidative stress response

**Figure 2:**
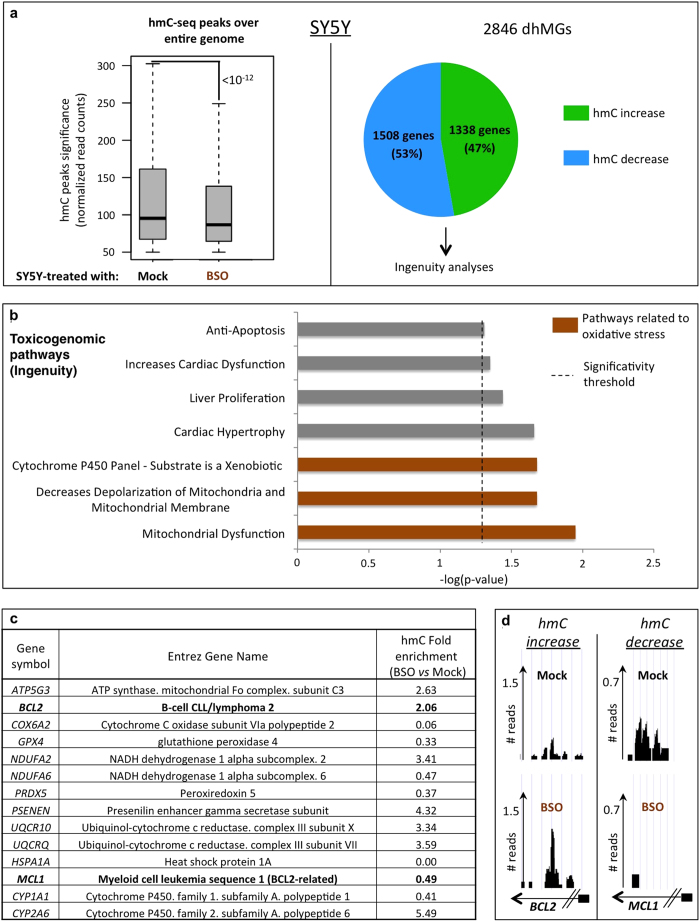
Genome-wide hmC sequencing after cell treatment with BSO highlights oxidative-stress-related pathways. (**A**) Illumina deep sequencing was done on DNA from mock- and BSO-treated SY5Y cells after selective isolation of hydroxymethylated DNA fragments. Upper left panel: The global decrease in hmC (already demonstrated on dot blot and mass spectrometry, see Fig. 1B) is illustrated by a significant decrease (p-value < 10^−12^) in the normalized hmC- read count in BSO-treated cells. Upper right panel: Differentially hydroxymethylated genes (dhMGs) used in Ingenuity analyses. (**B**) Ingenuity toxicogenomic pathway analysis (“IPA-Tox”) of the dhMGs identified in SY5Y cells shows over-representation of oxidative-stress-related pathways (marked in brown). The *x* axis represents the log(p-value) and the dashed line shows the significance threshold for pathway over-representation. (**C,D**) Gene list and UCSC sequencing tracks associated with the oxidative-stress-related pathways shown in Fig. 2B. Gene symbols, Entrez gene names, and hmC fold changes for BSO- *vs.* mock-treated SY5Y cells are depicted. Normalized *BCL2* and *MCL1* read counts are represented in (**D**) as examples. Small black boxes attached to arrows represent the promoters of these genes. See also supplementary Fig. S2.

The global decrease in hmC seen upon treatment of SY5Y cells with BSO (Fig. 1B) led us to interrogate its genome-wide distribution. For this we used the previously described hmC-selective chemical labeling technique *hMe-seal* to selectively isolate hydroxymethylated DNA fragments^17^, and subjected these to Illumina deep sequencing (referred here as “hmC-seq”). As previously described (reviewed in^18^), gene bodies appeared most highly represented among the captured fragments, exons being more enriched than introns (supplementary Fig. S2A). In agreement with the above dot-blot and mass spectrometry data, BSO-treated SY5Y cells displayed a significant global decrease in hmC (Fig. 2A, left panel).

Despite the observed global decrease of hmC, we next looked at differentially hydroxymethylated genes (termed “dhMGs”) to see if certain genes might locally loose or even gain hmC upon oxidative stress. We found 2846 dhMGs (supplementary table I), 53% of which displayed a local decrease in hmC and 47% a local increase (Fig. 2A, right panel). Remarkably, Ingenuity gene ontology analysis applied to these dhMGs revealed significant over-representation of toxicogenomic pathways associated with oxidative stress response, such as mitochondrial dysfunction, decreased polarization of mitochondria, and cytochrome P450 response (Fig. 2B). Of note, the most highly over-represented pathways were different according to whether a differentially hydroxymethylated gene showed a gain or a loss of hmC: the mitochondrial dysfunction pathway in the former case and pathways related to the physiopathology of the heart, liver and kidney in the latter (supplementary Fig. S2B).

Interestingly, genes such as *BCL2* or the *BCL2*-related *MCL1* gene, found to be differentially hydroxymethylated (Fig. 2C; see also sequencing tracks on Fig. 2D), are known to exert important functions during the oxidative stress response: Oxidative stress is attenuated in mice overexpressing *BCL2*, and the hepatitis B HBx protein sensitizes liver cells to H_2_O_2_-induced oxidative stress by reducing *MCL1* expression^19^,^20^.

Our results thus show that BSO treatment affects the hmC patterns both globally and locally, notably in genes important for a protective response to oxidative stress.

### Mice lacking the glutathione peroxidases 1 and 2 display an altered hydroxymethylation on genes involved in the oxidative stress response

**Figure 3:**
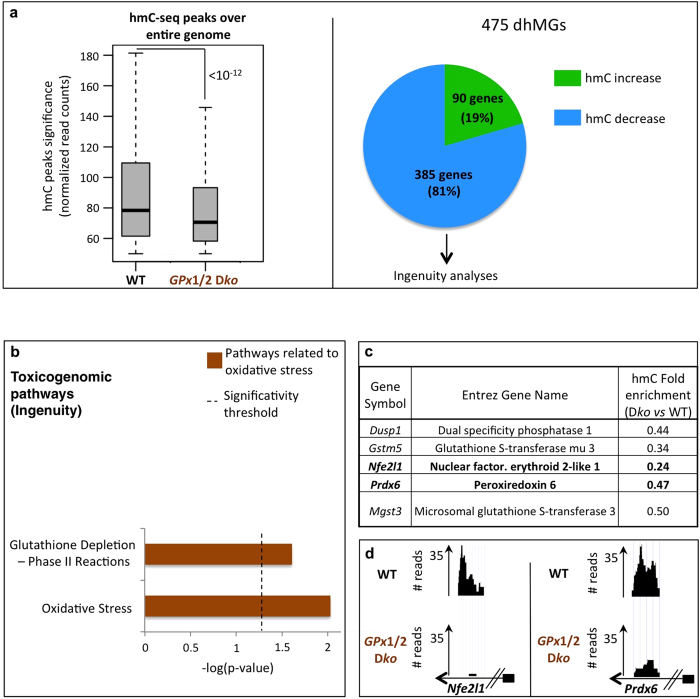
Mice lacking *GPx*1/2 display genome-wide hmC changes in genes involved in oxidative-stress-related pathways. (**A**) Left panel: Illumina deep sequencing was done on captured hydroxymethylated DNA fragments from wt and *GPx*1/2 D*ko* colon epithelial cells. A global decrease in hmC is observed in *GPx*1/2 D*ko* cells (p-value < 10^−12^), representative experiment. Right panel: This panel shows dhMGs that were common in two independent sequencing experiments, and to which Ingenuity analysis was applied. (**B**) IPA-Tox analysis of dhMGs identified in mice reveals significant over-representation of oxidative-stress-related pathways (marked in brown). The log(p-value) is plotted on the *x* axis and a dashed line shows the significance threshold for pathway over-representation. (**C,D**) Gene list and UCSC sequencing tracks associated with the oxidative-stress-related pathways in Fig. 3B. Gene symbols, Entrez gene names, and hmC fold changes for *GPx*1/2 D*ko vs.* wt are depicted. (**D**) Normalized *Nfe2l1* (*Nrf1*) and *Prdx6* read counts are represented as examples. Small black boxes attached to arrows represent the promoters of these genes (representative experiment). See also supplementary Fig. S3.

As for the above, hmC-seq experiments were performed on *GPx*1/2 wt and D*ko* colon epithelia, and confirmed the results obtained with SY5Y cells: mice lacking the *GPx* genes showed a significant overall decrease in hmC (Fig. 3A, left panel); reads most often corresponding to a gene body location and more frequently to an exon than to an intron location (supplementary Fig. S3A).

We next identified 475 dhMGs (supplementary table II), 81% of which showed a local decrease in hmC and 19%, a local increase (Fig. 3A, right panel). Interestingly, a significant proportion of dhMGs were found to be involved in toxicogenomic pathways associated exclusively with oxidative stress, and notably with GSH depletion, precisely emphasizing *GPx* deletion that occurs in the D*ko* mice (Fig. 3B,C. In supplementary figure S3B, results are shown separately for genes showing a gain or loss of hmC).

As for BSO-treated SY5Y cells, we again found key genes involved in the oxidative stress response. Examples include *Nfe2l1*/*Nrf1* and *Prdx6* (Fig. 3C,D), whose products are known, respectively, to initiate transcription of antioxidant genes and to participate in the redox regulation of the cell by reducing H_2_O_2_^21^,^22^. It is also worth noting that our pathway analyses did not highlight the same pathways in BSO-treated SY5Y cells and *GPx*1/2 D*ko* mice (Figs 2B and 3B). This probably reflects the fact that the cultured cells were treated for only 48 h, whereas mice lacking the *GPx* genes were under constant oxidative assault from embryogenesis to the day of sacrifice (1 month).

In summary, these results confirm restructuration of the hydroxymethylome, here in response to an *in vivo* oxidative stress, and uncover a set of genes/pathways that reflect the need for an appropriate cellular response.

### MicroRNAs encoded by differentially hydroxymethylated genes are predicted to target transcripts involved in the oxidative stress response

**Figure 4:**
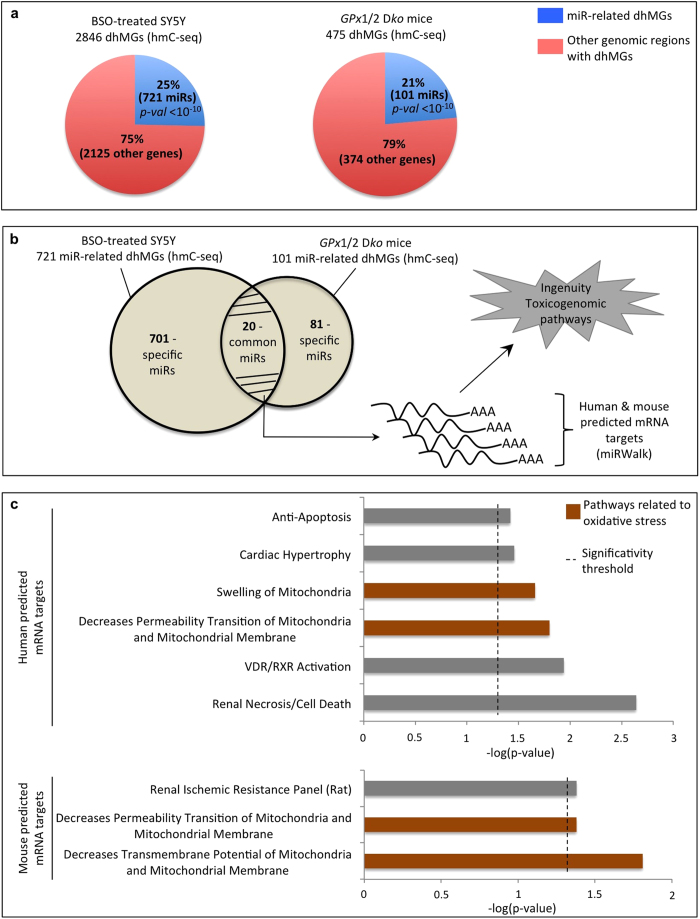
Differentially hydroxymethylated microRNA genes highlight pathways involved in the oxidative stress response. (**A**) Percentage of dhMGs corresponding to microRNA-encoding sequences as compared to other genes in human SY5Y cells and mouse cells. (**B**) A set of 20 dhMG-associated miR genes, common to human and mouse, were identified and their targets predicted. Next, the identified mRNA targets were processed with the Ingenuity software. (**C**) IPA-Tox applied to the predicted S5Y5 and mouse mRNA targets reveals significant over-representation of oxidative-stress-related pathways (marked in brown). In each graph, the *x* axis represents the log(p-value) and the dashed line, the significance threshold for pathway over-representation. See also supplementary Fig. S4.

During our analysis of dhMGs in SY5Y cells and *GPx*1/2 wt and D*ko* mice, we observed an unexpected high proportion of differentially hydroxymethylated microRNA-encoding sequences (Fig. 4A). MicroRNAs (miRs) are non-coding RNAs of 18 to 24 nucleotides long that hybridize to target mRNAs and, depending on the level of complementarity, cause their degradation or translational repression^23^. As depicted in Fig. 4A, 25% of the dhMGs identified in SY5Y cells and 21% of those identified in *GPx*1/2 D*ko* mice were found to correspond to miR-encoding sequences (see also supplementary table III).

As many miR targets are conserved among mammalian species^24^, we examined more closely the miR-encoding sequences showing altered hydroxymethylation in BSO-treated SY5Y cells or in *GPx*1/2 D*ko* mice. Of these miRs, 20 showed a robust increase or decrease in hmC in both SY5Y cells and in *GPx*1/2 D*ko* mice (Fig. 4B and supplementary Fig. S4). Focusing on this set of 20 miRs, we used the miRWalk database, a bioinformatic tool that exploits several prediction programs^25^, to build a comprehensive list of predicted mRNAs targets (Fig. 4B, and supplementary table IV). Surprisingly, Ingenuity pathway analysis applied to the corresponding human and mouse mRNAs revealed over-representation of toxicogenomic pathways involved in oxidative stress, as observed earlier (Fig. 4C; see also Figs 2B and 3B, and supplementary Figs S2B and S3B).

These results thus suggest that hydroxymethylation pattern changes in DNA regions that do not solely code for proteins (here, microRNA genes), but that may contribute to determine the cellular response to oxidative stress.

## Discussion

In this report, we have used different approaches to investigate the links between oxidative stress, the global hmC level, and the local distribution of this epigenetic mark. *In vitro* and *in vivo* data on SY5Y cells and in our mouse model show that hmC is significantly decreased upon oxidative stress. In addition, we show that TET1 depletion sensitizes SY5Y cells to oxidative stress induced by BSO, the most commonly used drug to increase intracellular levels of reactive oxygen species (ROS). This suggests that TET1, at least in part, may play a role in protecting cells against oxidative stress.

Of note, our genome-wide hmC profiling data shows a global decrease of hmC in both SY5Y cells and in *GPx*1/2 D*ko* mice, but locally, some of the differentially hydroxymethylated regions display an increase of hmC in response to oxidative stress. This increase at specific genomic loci might be caused by recruitment of TET enzymes to these regions in order to initiate an appropriate transcriptional program to respond to oxidant assaults.

Surprisingly, around 25% of the dhMGs identified here in either SY5Y or mice lie in microRNA-encoding sequences. *In silico* analyses have revealed that these miRs could target important genes involved in ROS detoxification. MiRs have been shown to target the transcripts of numerous genes that are associated to life-threatening diseases such as neurodegenerative disorders or cancer, and this set of miRs might be exploited, in the future, in biomarker discovery for oxidative stress related diseases^26^,^27^. The present work could thus be the starting point in the development of exciting biomedical applications, once we have gained better understanding of the links between oxidative stress and hydroxymethylcytosine patterns, and between miR-genes hydroxymethylation and the miR-genes transcript levels.

Of note, our study is the first that evaluated the impact of oxidative stress *in vivo.* On the one hand, prolonged oxidative stress is associated with inflammation, which is thought to precede cancer development^28^. In the other hand, a decrease of hmC is now widely accepted as a hallmark of many cancers, linking the TET proteins to cancerogenesis^29^. It is thus tempting to speculate that the decrease of hmC observed during cancer development could be explained, at least partly, by a decrease of hmC emerging during oxidative stress and inflammation.

In conclusion, we propose, on the basis of our results, a model linking oxidative stress to hydroxymethylcytosine patterns. Our results notably highlight the unexpected potential role of certain microRNAs in determining how cells respond to oxidative stress. In addition, we expect that these results, in association with other studies, will lift the veil on molecular aspects that could explain the global decrease of hmC in cancers. However, additional investigation on how the TETs are recruited on genes that gain hmC, as well as the transcriptional effects of differential hydroxymethylation on oxidative stress-related genes and microRNAs, still need to be addressed and should be the subject of future studies.

## Methods

### Statistical analysis

Unless otherwise indicated, all experiments included technical replicates, and were repeated at least three independent times. Data and graphs are presented as averages ± standard deviations. Data were compared by means of two-tailed unpaired *t*-tests for comparison, and statistical significance was accepted at p-values ≤0.05. * and ** represent p-values ≤0.05 and ≤0.01, respectively. Statistics relative to hmC deep-sequencing analysis are further described in supplementary methods. Ingenuity p-values were calculated online with Fisher’s exact *t*-test.

### Cell culture and treatments

SY5Y cells (ATCC #CRL- 2266) were maintained in 1:1 Dubelcco’s modified Eagle’s medium (DMEM) and F12 medium, then supplemented with 10% fetal bovine serum and grown at 37 °C under 5% CO2. The cells were then treated with 500 μM BSO for 48 h. The mock-treatment control contained water instead of BSO.

### *GPx*1/2-knockout mice

In our *in vivo* experiments, purified genomic DNA extracts obtained from the colon epithelia of *129* mice with combined disruption of the *GPx*1 and *GPx*2 genes were generously provided by Dr. F. F. Chu, and are fully described in^10^. The methods used on mice were carried out in accordance with the approved NIH guidelines, which authorize to perform all of the animal studies described in the related manuscript. In addition, all experimental protocols were approved by the COH Research Animal Care Committee (Duarte, California).

Five wild-type and five double-knockout (D*ko*) mice were used for hmC quantification in dot-blot and mass spectrometry experiments. Of these, two wild-type and two D*ko* mice were used for subsequent deep-sequencing analysis.

### Dot blot for 5-hydroxymethylcytosine quantification

Dot-blot experiments were done as previously described, with some modifications^30^. For complete procedure, please refer to supplementary methods.

### Western blot analyses

Standard procedures were used for Western blotting^31^. Primary antibodies used in these experiments were directed against TET2 (ab94580; Abcam) or HDAC1 (Diagenode; pAb-053-050).

### Analysis of global hmC levels by mass spectrometry (LC/MS/MS)

500 ng of genomic DNA was incubated with 5 U of DNA Degradase Plus (Zymo Research) at 37 °C for 3 h. The resulting mixture of 2’-deoxynucleosides was analysed on a Triple Quad 6500 mass spectrometer (AB Sciex) fitted with an Infinity 1290 LC system (Agilent) and an Acquity UPLC HSS T3 column (Waters), using a gradient of water and acetonitrile with 0.1% formic acid. External calibration was performed using synthetic standards, and for accurate quantification, all samples and standards were spiked with isotopically labeled nucleosides. HmC levels are expressed as a percentage of total cytosines.

### Hydroxymethylated DNA fragment affinity purification (*hMe-seal*)

At least 500 ng of genomic DNA was diluted in ultra-pure water to 35 ng/μL and then sonicated in cold water with a Bioruptor sonicator (Diagenode) to obtain fragments averaging 300 bp in size. The fragmented DNA was used in combination with the hydroxymethyl collector (Active Motif) following to the manufacturer’s protocol. Briefly, a glucose moiety that contains a reactive azide group was enzymatically linked to hydroxymethylcytosine in DNA, creating glucosyl-hydroxymethylcytosine. Next, a biotin conjugate was chemically attached to the modified glucose *via* a “click reaction”, and magnetic streptavidin beads were used to capture the biotinylated-hmC DNA fragments. After extensive washing steps and chemical elution, the hydroxymethylated DNA fragments released from the beads were used in sequencing experiments.

### Ingenuity software

Ingenuity IPA software was used for toxicogenomic analysis (“IPA-TOX”). The genes and hmC fold changes were loaded into the Ingenuity database and then core analyses were done, using default parameters and specifying the relevant species (“Human” for SY5Y cells and “Mouse” for mice samples). The tissues used in the experiments were also indicated.

For further details on experimental procedures, including, RNA interference and RT-qPCR analysis, dot blot for 5-hydroxymethylcytosine quantification, Reactive Oxygen Species measurements, measures of apoptosis and necrosis, proliferation experiments, library preparation, deep sequencing workflow, and statistical analysis, please refer to supplementary methods.

## Additional Information

**How to cite this article**: Delatte, B. *et al.* Genome-wide hydroxymethylcytosine pattern changes in response to oxidative stress. *Sci. Rep.* **5**, 12714; doi: 10.1038/srep12714 (2015).

## Supplementary Material

Supplementary Information
