# Supplementary material for: Genome-wide hydroxymethylcytosine pattern changes in response to oxidative stress
Source: Sci Rep. 2015 Aug 4;5:12714. doi: 10.1038/srep12714 (PMC4523844; doi:10.1038/srep12714)
Supplement: Supplementary Information [file srep12714-s1.pdf]

Genome-wide hydroxymethylcytosine pattern changes in response to oxidative stress

Benjamin Delatte[1,*], Jana Jeschke[1], Matthieu Defrance[1], Martin Bachman[2], Catherine Creppe[1], Emilie Calonne[1], Martin Bizet[1], Rachel Deplus[1], Laura Marroquí[3], Myriam Libin[4], Mirunalini Ravichandran[5], Françoise Mascart[4], Decio L. Eizirik[3], Adele Murrell[2], Tomasz P. Jurkowski[5] & François Fuks[1,*]

[1]Laboratory of Cancer Epigenetics, Faculty of Medicine, ULB, 1070 Brussels, Belgium.

[2] CRUK Cambridge Institute, University of Cambridge, Cambridge CB2 0RE, United Kingdom.

[3]ULB, Center for Diabetes Research, Faculty of Medicine, ULB, 1070 Brussels, Belgium.

[4]Laboratory of Vaccinology and Mucosal Immunity, Faculty of Medicine, ULB, 1070 Brussels, Belgium.

[5]Institute of Biochemistry, Stuttgart University, 70569 Stuttgart, Germany.

* Corresponding authors:

Benjamin Delatte: Tel: 32-2-555.60.16; E-mail: benche65@gmail.com

François Fuks: Tel: 32-2-555.62.45; Fax: 32-2-555.62.57. E-mail: ffuks@ulb.ac.be

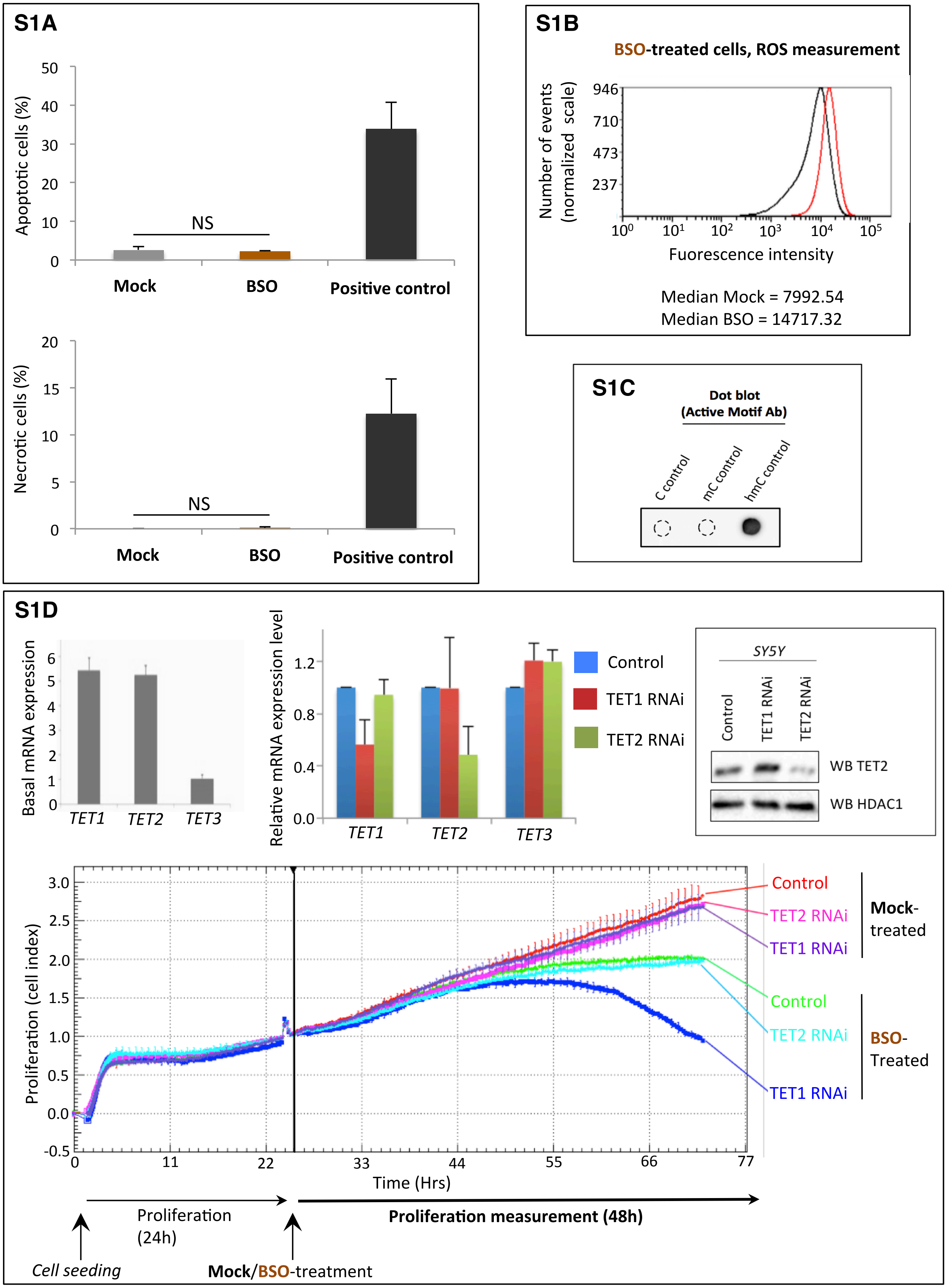

Supplementary Figure 1. Delatte *et al.*

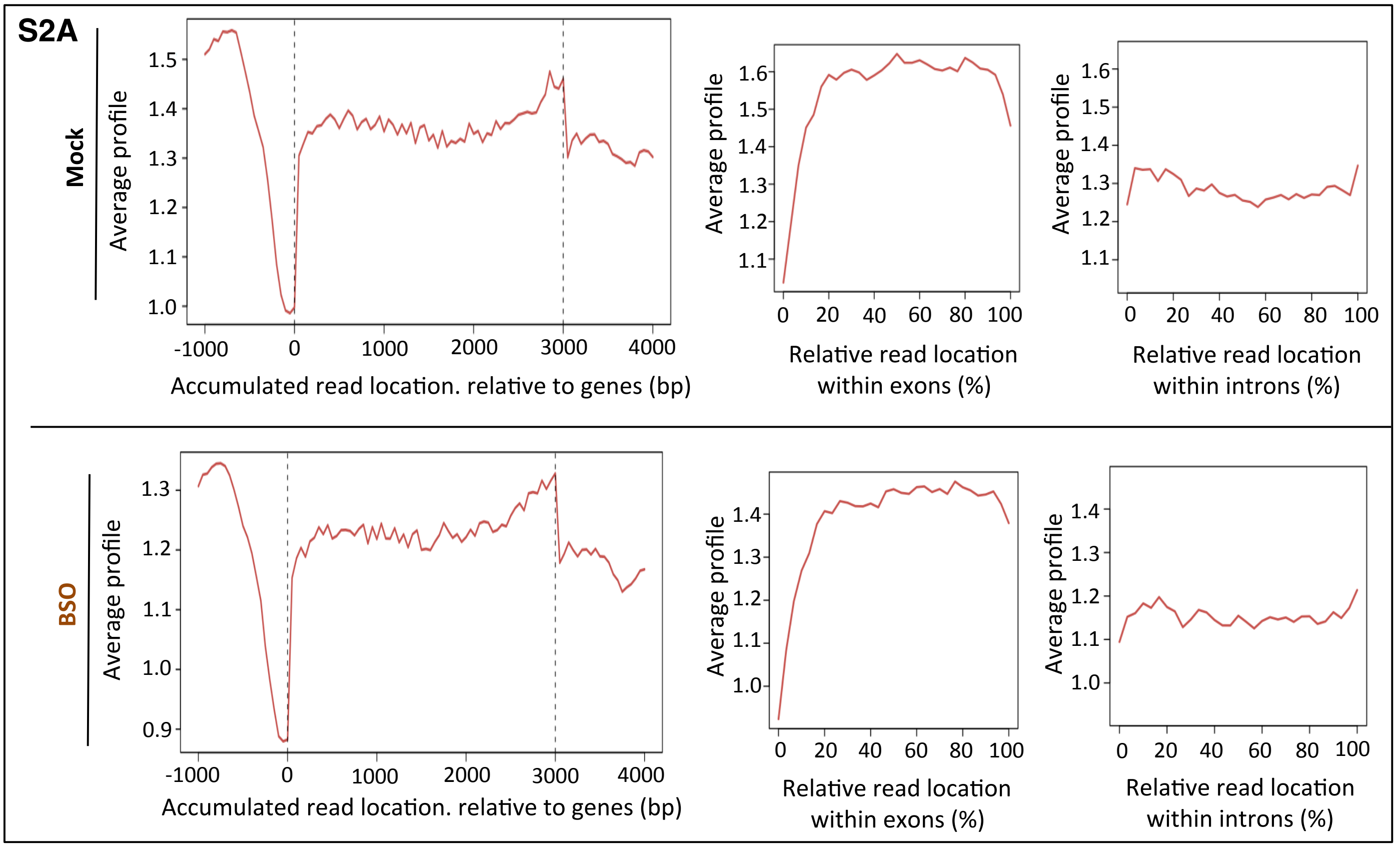

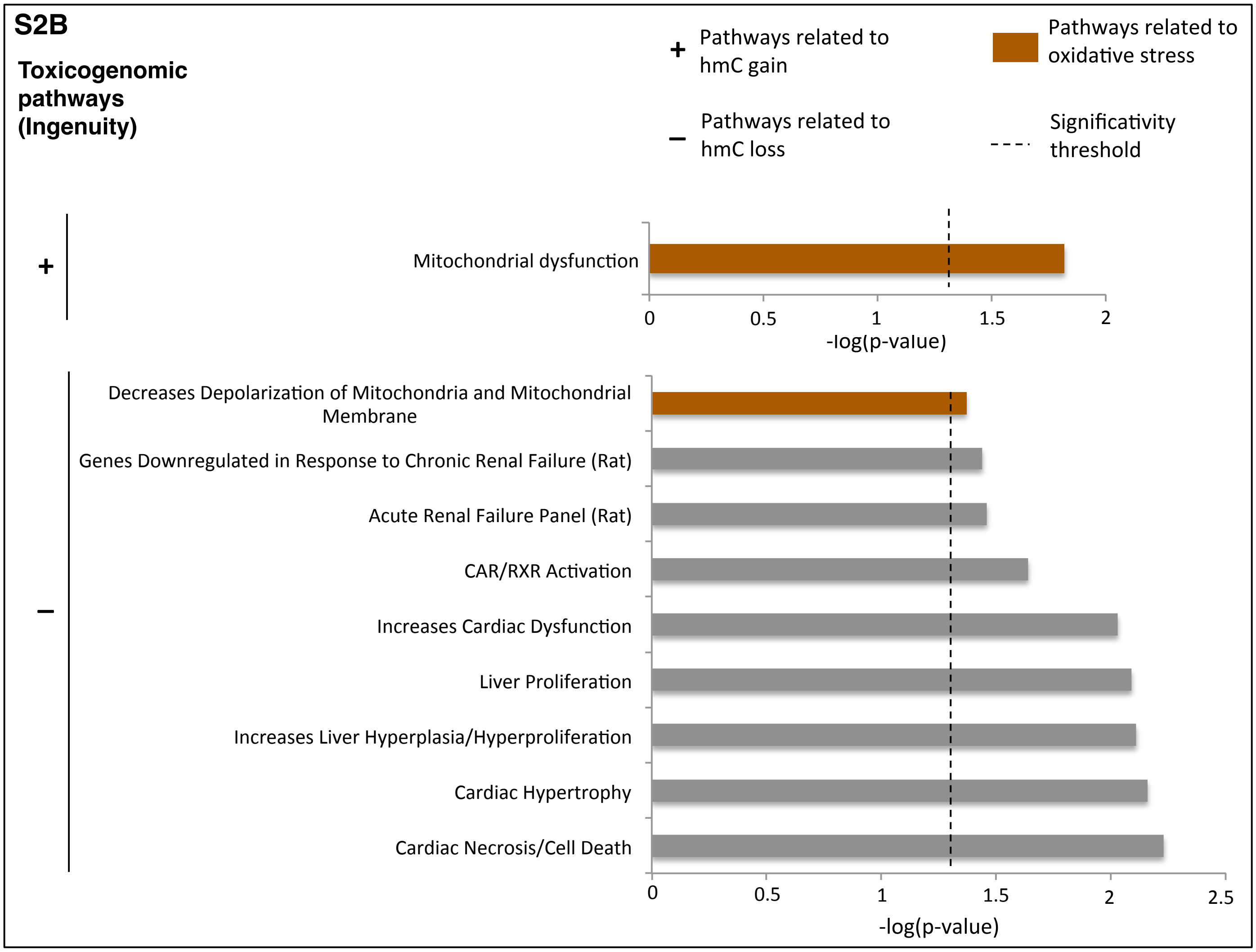

Supplementary Figure 2. Delatte *et al.*

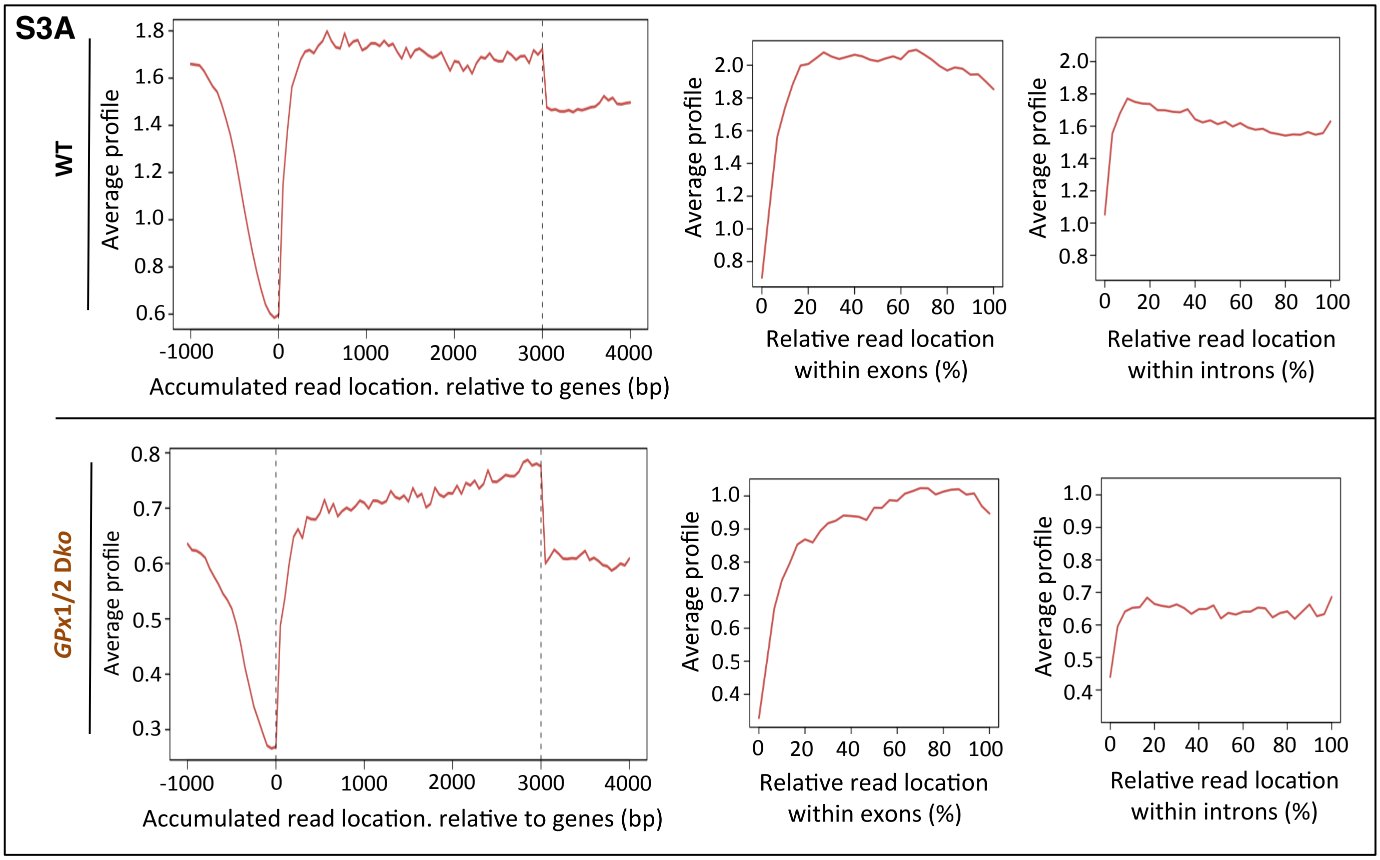

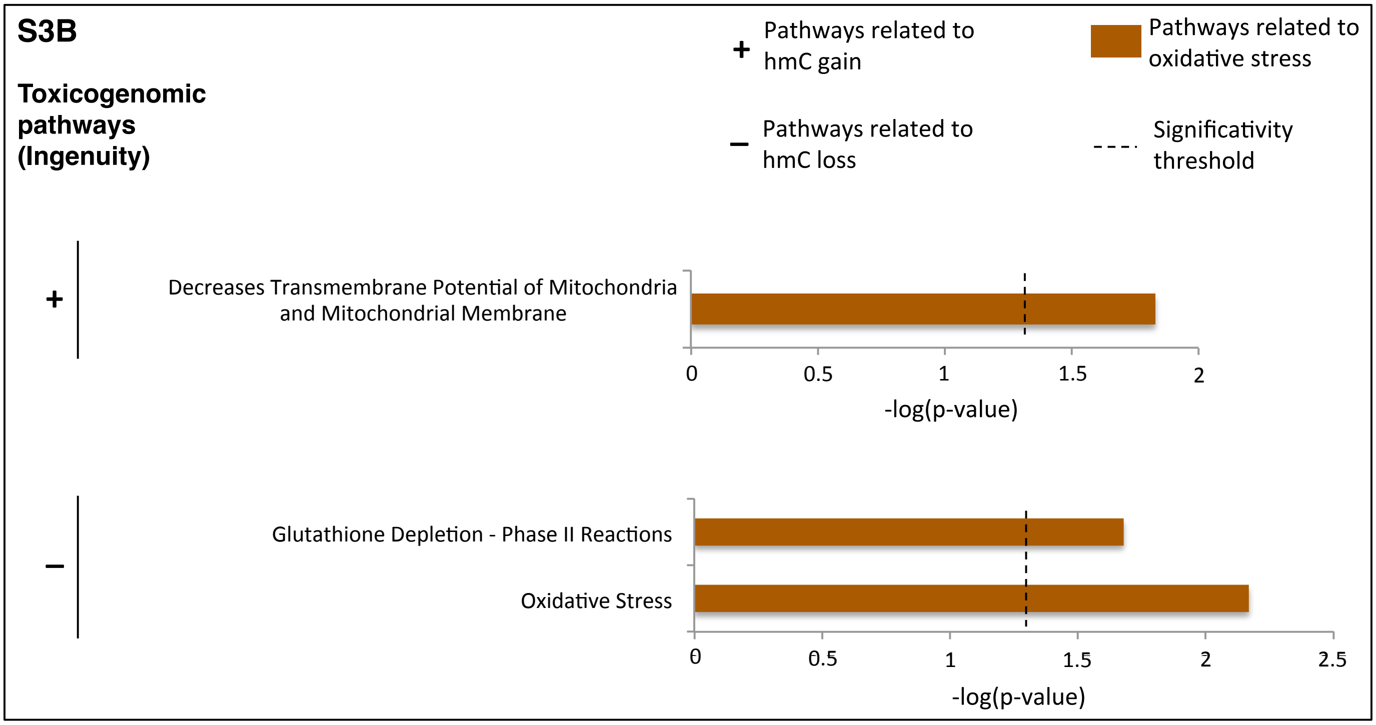

Supplementary Figure 3. Delatte *et al.*

S4

Common miRs contained in dhMGs (SY5Y and mice samples)	Increase (+) or decrease (-) hmC in BSO *vs* mock -treated SY5Y	Increase (+) or decrease (-) hmC in *GPx*1/2 D*ko* *vs* wt mice
**miR-1247**	**-**	**-**
**miR-137**	**+**	**+**
**miR-148a**	**+**	**+**
**miR-148b**	**+**	**+**
**miR-15a**	**-**	**-**
**miR-191**	**-**	**-**
**miR-193b**	**-**	**-**
**miR-202**	**+**	**+**
**miR-22**	**+**	**+**
**miR-30a**	**-**	**-**
**miR-31**	**-**	**-**
**miR-449a**	**+**	**+**
**miR-488**	**-**	**-**
**miR-497**	**+**	**+**
**miR-532**	**-**	**-**
**miR-675**	**+**	**+**
**miR-7-2**	**-**	**-**
**miR-877**	**+**	**+**
**miR-let-7d**	**-**	**-**
**miR-let-7g**	**-**	**-**

Supplementary Figure 4. Delatte *et al.*

Supplementary methods

RNA interference and RT-qPCR analysis

Interfering RNAs for TET1 and TET2 were generated as previously described [1]. Briefly, the target sequence used to silence TETs (primers available upon request) were inserted as a short hairpin into the pRetroSuper (pRS) retroviral vector according to the manufacturer's recommendations (OligoEngine), to form TET1 or TET2 RNAi. Retrovirus production by HEK293GP cells and infection of SY5Y were performed as described in [1]. Infected cells were selected with 1.5 µg/ml puromycin (Sigma). RNA purification and RT-qPCR analysis were performed as described previously [1]. For supplementary Fig S1D (upper right panel), RT-qPCR analysis was performed on mRNAs from TET1- and TET2-knockdown cells (TET1, TET2 RNAi) and results are expressed relatively to the control (pRS-empty). In supplementary Fig S1D (upper left panel), the results are normalized to the level of *HPRT* housekeeping gene. Primers are available upon request.

Dot blot for 5-hydroxymethylcytosine quantification

Isolated genomic DNA was incubated at 95°C for 10 min and immediately chilled on ice to obtain single-stranded DNA. Then, 1.5 µl (approx. 500nG) DNA was spotted in triplicate onto a nylon membrane (GE Healthcare Hybond-N+). After drying the membrane, cross-linking was performed twice with 200 000 µJ/cm^2 of UV. The membrane was blocked overnight at 4°C in 10% (w/v) non-fat dry milk + 1% (w/v) BSA in PBS+0.1% Tween 20. The membrane was then transferred into blocking solution supplemented with rabbit anti-hmC polyclonal antibodies (from Active Motif) diluted 1:1000. This antibody was validated for specificity in [2,3] and further validated as shown in supplementary Fig S1C. The quantity of input DNA was assessed by incubation for 1 h on a stripped membrane with human anti-ssDNA antibody (diluted 1:100)

(LSBio). After incubation with the primary antibody, the membrane was washed 3 times for 10 min with PBS+0.1% Tween 20 and transferred into blocking solution supplemented with HRP-conjugated anti-rabbit isotype (GE Healthcare) (diluted 1:5000) or anti-human isotype (Jackson Immuno Research) (diluted 1:10,000), and incubated for 1 h at room temperature. This was followed by 3 washing steps with PBS+0.1% Tween 20. Peroxidase activity was detected by ECL (Western Lightning Plus-ECL, Perkin-Elmer) and signal detection was done with the Bio-Rad ChemiDoc system (Bio-Rad). For spot signal quantification, imageJ64 software was used. Membrane stripping was performed in PBS supplemented with 20% SDS and 1.5% β-mercaptoethanol for 30 minutes at 50°C, then the membrane was washed extensively with PBS+0.1% Tween 20.

Reactive Oxygen Species (ROS) measurements

Cellular ROS production was determined by CM-H2DCFDA staining (Life technologies) by following the manufacturer's protocol. This chloromethyl derivative of H2DCFDA passively diffuses into cells, where its acetate groups are cleaved by intracellular esterases and its thiol-reactive chloromethyl group reacts with intracellular glutathione and other thiols. Subsequent oxidation produces a fluorescent adduct that is trapped inside the cells, and that can be measured by fluorescence, in our case, by FACS measurement. In brief, SY5Y cells were incubated in PBS containing 5µM of the CM-H2DCFDA dye for 20 min at 37°C, then cells were further incubated in complete medium for 20 min at 37°C and were trypsinized. Measurements (on the FITC channel) were done on a FACSCanto II flow cytometer (BD Biosciences).

Measures of apoptosis and necrosis

Percentages of viable, apoptotic, and necrotic cells were determined on mock-treated SY5Y cells and on SY5Y cells treated for 48 h with 500 µM of BSO or 5 µM rotenone (as a positive control

for apoptosis and necrosis) after a 15-min incubation with the DNA-binding dyes propidium iodide (PI, 5 µg/ml; Sigma) and Hoechst 33342 (HO, 5 µg/ml; Sigma), as previously described [4,5]. A minimum of 600 cells was counted for each experimental condition. Apoptosis and necrosis were evaluated by two independent observers, one of them unaware of sample identity. Agreement between the findings of the two observers was >90%. Results are expressed as percentages calculated by dividing the number of apoptotic or necrotic cells by the total number of cells and multiplying by 100.

Proliferation experiments (with the xCELLigence device)

To evaluate SY5Y cell proliferation upon TET depletion (see above) and upon treatment with BSO, cells were seeded into the xCELLigence E-plate 16 (Roche) at 5000 cells/well according to the manufacturer's instructions, and as described in Denis *et al.* (manuscript in preparation). In this system, the electrical impedance is used to derive a cell index which, when continually monitored, gives a real-time representation of cell growth. Measurements were automatically collected with the RTCA DP analyzer every 40 min for up to 48 h. Three technical replicates were produced per condition and the experiment was done at least three independent times. The data were then analyzed with the provided RTCA software.

Library preparation, deep sequencing workflow, and data analysis

Library preparation was done with the TruSeq ChIP Sample Prep Kit (Illumina) according to the manufacturer's instructions. Briefly, captured hydroxymethylated DNA fragments were subjected to 5' and 3' protruding end repair, and non-templated adenines were added to the 3' ends of the blunted DNA fragments. Illumina adapters were ligated and DNA was size selected with a 2% agarose gel cassette (E-gel, Life technologies) in order to remove all unligated adapters and to select for 300-bp fragments. Next, 18 cycles of PCR were done to amplify the library. DNA was

then quantified by fluorometry with the Qubit 2.0 fluorometer (Life technologies) and its integrity was assessed with the Agilent 2100 BioAnalyzer (Agilent). 6 pM of DNA library, spiked with 1% PhiX viral DNA, was clustered on cBot (Illumina), and then sequencing was performed on a HiScanSQ module (Illumina). The BWA software [6] was used to map sequencing reads that were mapped to the human genome (NCBI Build 36 / UCSC hg18) (SY5Y cells) or to the mouse genome (NCBI Build 37 / UCSC mm9) (mouse samples). Reads not uniquely mapping to the reference genome were discarded. Raw data are available under the Gene Expression Omnibus [http://www.ncbi.nlm.nih.gov/geo/query/acc.cgi?token=cfgzooqetvulvgl&acc=GSE59718] database [GSE59718] ID. Read density was computed by removing duplicate reads (*i.e.* mapping to the same location) and by normalizing the local read count with respect to the total read count. Local enrichment in hmC was evaluated with the MACS software [7], with a p-value cut-off < 10e-10. Differences between hmC levels were assessed for all annotated RefSeq transcripts (Gene Body) by first counting the normalized number of reads (FPKM) and then computing the fold change (FPKM condition 1 / FPKM condition 2) and the absolute delta value (FPKM condition 1 - FPKM condition 2) between the two conditions. Genes were sorted according their absolute fold change after discarding genes with a low absolute difference (absolute delta) to avoid artifactual effects. hmC profiles around the TSS and within exons and introns were computed by first computing the local enrichment with the MACS software and then annotating the enrichment with the CEAS software [8]. All statistics and plotting were done with the R statistical software. To obtain sequencing tracks (Fig 2D and 3D), wig files were uploaded onto the UCSC genome browser.

Supplementary references

1 Vire, E. *et al.* The Polycomb group protein EZH2 directly controls DNA methylation. *Nature* **439**, 871-874, doi:10.1038/nature04431 (2006).

2 Ito, S. *et al.* Role of Tet proteins in 5mC to 5hmC conversion, ES-cell self-renewal and inner cell mass specification. *Nature* **466**, 1129-1133, doi:10.1038/nature09303 (2010).

3 Deplus, R. *et al.* TET2 and TET3 regulate GlcNAcylation and H3K4 methylation through OGT and SET1/COMPASS. *EMBO J* **32**, 645-655, doi:10.1038/emboj.2012.357 (2013).

4 Kutlu, B. *et al.* Discovery of gene networks regulating cytokine-induced dysfunction and apoptosis in insulin-producing INS-1 cells. *Diabetes* **52**, 2701-2719 (2003).

5 Rasschaert, J. *et al.* Toll-like receptor 3 and STAT-1 contribute to double-stranded RNA+ interferon-gamma-induced apoptosis in primary pancreatic beta-cells. *J Biol Chem* **280**, 33984-33991, doi:10.1074/jbc.M502213200 (2005).

6 Li, H. & Durbin, R. Fast and accurate short read alignment with Burrows-Wheeler transform. *Bioinformatics* **25**, 1754-1760, doi:10.1093/bioinformatics/btp324 (2009).

7 Zhang, Y. *et al.* Model-based analysis of ChIP-Seq (MACS). *Genome Biol* **9**, R137, doi:10.1186/gb-2008-9-9-r137 (2008).

8 Shin, H., Liu, T., Manrai, A. K. & Liu, X. S. CEAS: cis-regulatory element annotation system. *Bioinformatics* **25**, 2605-2606, doi:10.1093/bioinformatics/btp479 (2009).

Supplementary Figure S1 (related to Figure 1). Apoptosis, necrosis, and ROS measurements; dot blot controls and cell proliferation.

(A) Percentages of apoptotic (upper panel) and necrotic (lower panel) cells measured by fluorescence microscopy on SY5Y cells subjected to mock treatment, or treated with 500 µM of BSO and 5 µM of rotenone (as a positive control for apoptosis/necrosis) for 48 h. Data are means ± standard deviations of at least 3 independent experiments. NS = non-significant. **(B)** Reactive oxygen species (ROS) were quantitated by FACS analysis with a CM-H2DCFDA probe after treatment of SY5Y cells with 500 µM of BSO. Peak shifts towards higher fluorescence intensities in BSO-treated (red peak) versus mock-treated (black peaks) cells, and are indicative of increased intracellular levels of ROS in the former cell population. Median intensities for this representative experiment are also shown. **(C)** Specificity of the anti-hmC antibody used in our experiments: controls with *in vitro* PCR fragments containing only non-modified cytosines (C control), methylated cytosines (mC control), or hydroxymethylated cytosines (hmC control). Dashed circles denote the total absence of signal. **(D)** Proliferation of SY5Y cells after knockdown of *TET1* or *TET2* expression. Upper left panel: Basal levels of *TET1*, *TET2*, and *TET3* transcripts (relative to *HPRT*). Upper right panel: Levels of the three TET transcripts after treatment with *TET1*- or *TET2*-targeting interfering RNA (RNAi). Results are normalized with respect to the levels observed in control cells transfected with an empty vector. On the upper right panel: validation of the TET2-targeting RNAi at protein level by western blotting. HDAC1 levels confirm that equal amounts of extract were used. The lower panel shows proliferation curves obtained with the xCelligence device for control-, *TET1*- and *TET2*-knockdown cells, mock-treated or BSO-treated. Briefly, SY5Y cells were seeded and grown for 24 h and then the proliferation of mock- and 500-µM-BSO-treated cells was monitored continuously for 48 h.

Supplementary Figure S2 (related to Figure 2). Deep-sequencing profiles and Ingenuity toxicogenomic analyses of dhMGs showing an increase or a decrease of hmC in BSO-treated cells.

(A) Upper panels: Normalized average sequencing read counts obtained for mock-treated S5Y5 cells are plotted against the distance upstream or downstream from transcription start site (TSS). Average read accumulation within exons and introns is also shown. Lower panels: same as above, but for BSO-treated cells. **(B)** Toxicogenomic analyses (IPA-Tox) applied to dhMGs showing an hmC gain in SY5Y cells reveals over-representation of oxidative-stress-related pathways (marked in brown). IPA-Tox applied to dhMGs showing an hmC decrease reveals over-representation of other pathways, such as ones involved in heart or liver physiopathology. In each graph the log(p-value) is plotted on the *x*-axis and a dashed line indicates the significance threshold for pathway over-representation.

Supplementary Figure S3 (related to Figure 3). Deep-sequencing profiles and Ingenuity toxicogenomic analysis of dhMGs showing an increase or a decrease of hmC in *GPx*1/2 D*ko* mice.

(A) Upper panels: Average normalized sequencing reads corresponding to dhMGs are plotted as a function of distance upstream or downstream from the transcription start site (TSS) in epithelial cells from wt mice. Average read accumulation within exons and introns is also shown. Lower panels: Same as above, but for *GPx*1/2 D*ko* mice. Representative experiment. **(B)** IPA-Tox applied to dhMGs identified in cells from the large intestine of mice: oxidative-stress-related pathways (marked in brown) appear over-represented whether the dhMGs display a gain or a loss of hmC in *GPx*1/2 D*ko* *vs.* wt mice (data from the two sequencing experiments). In each graph the *x* axis shows the log(p-value) and a dashed line indicates the significance threshold for

pathway over-representation.

Supplementary Figure S4 (related to Figure 4). Differentially hydroxymethylated microRNA regions common in SY5Y cells and in mice samples.

A list of the 20 human (SY5Y) and mouse miRs is shown here. They were selected by comparing dhMGs in BSO- *vs.* mock-treated SY5Y and in *GPx*1/2 D*ko vs.* wt mice. A “+” means that there is an increase in hmC in BSO- *vs.* mock-treated SY5Y cells (column 2) and in *GPx*1/2 D*ko vs.* wt mouse samples (column 3). Accordingly, a “-” means that there is a decrease in hmC in BSO- *vs.* mock-treated SY5Y cells (column 2) and in *GPx*1/2 D*ko vs.* wt mouse samples.

Table I (related to Fig. 2a). Gene symbol and hmC Fold enrichment (BSO *vs* Mock).

Differentially hydroxymethylated regions (dhMRs) on gene-coding sequences, identified upon comparison of hmC profiles of BSO and mock -treated SY5Y cells.

Table II (related to Fig. 3a). Gene symbol, mice normalized intensity of reads, mean of the duplicate, and hmC Fold enrichment (D*ko* *vs* wt).

Differentially hydroxymethylated regions (dhMRs) on gene-coding sequences, identified upon comparison of hmC profiles of *GPx*1/2 double-knockout (D*ko*) and wild-type (wt) mice.

Table III (related to Fig. 4a). MiRs found in mice or in SY5Y cells.

Differentially hydroxymethylated MicroRNA genes identified upon comparison of hmC profiles of BSO and mock -treated SY5Y cells ("miRs-SY5Y"), and upon comparison of hmC profiles of *GPx*1/2 double-knockout and wild-type mice ("miRs-mice").

Table IV (related to Fig. 4b). MiRWalk Predicted mRNAs found in mice or in SY5Y cells, as well as common mRNAs found in mice and in SY5Y cells.

MiRWalk Predicted SY5Y and mice transcripts of the 20 differentially hydroxymethylated miRs identified upon comparison of hmC profiles of BSO and mock -treated SY5Y cells, and upon comparison of hmC profiles of *GPx*1/2 double-knockout and wild-type mice.

Table I Gene symbol and hmC Fold enrichment (BSO vs Mock), related to Fig. 2a

Gene symbol	hmC Fold enrichment (BSO vs Mock)
USP9Y	inf
NLGN4Y	inf
NBPF24	inf
NBPF11	inf
THAP4	inf
GPR89C	inf
BTNL8	inf
TXLNG2P	inf
HGD	inf
NBPF24	inf
LOC100130000	inf
BCORP1	inf
BMS1P5	inf
TXLNG2P	inf
LOC653501	inf
CRLF2	inf
CRLF2	inf
NPIPA3	inf
NPIPA2	inf
RIMBP3B	inf
SPACA5	inf
DGAT1	inf
GOLGA6L1	inf
UGT2B15	inf
PLGLB2	inf
PLGLB1	inf
TRIM53AP	inf
ANAPC11	inf
ANKRD20A3	inf
ANKRD20A2	inf
ARHGEF35	inf
PRAMEF5	inf
PRAMEF6	inf
LOC100133267	inf
SPATA31A2	inf
SPATA31A1	inf
TMEM167B	inf
EIF3CL	inf
EIF3C	inf
TCP11X2	inf
SCRT1	inf
DUX4L	inf

OPN1MW	inf
DEFB107A	inf
TAF9B	inf
OPN1MW2	inf
LOC151009	inf
PRAMEF6	inf
LOC440894	inf
TMSB4Y	inf
DEFB106B	inf
PRAMEF23	inf
LOC100133267	inf
GAGE2E	inf
FAM225A	inf
RIMBP3C	inf
KIF22	inf
PRAMEF13	inf
CTAGE4	inf
OR6T1	inf
OR2W1	inf
OR7A17	inf
FFAR3	inf
SPATA31D4	inf
FAM27A	inf
PRAMEF16	inf
AMY1A	inf
LOC100287728	inf
AMY1A	inf
AMY1C	inf
AMY1B	inf
PRAMEF10	inf
PRAMEF22	inf
MTRNR2L8	inf
KRTAP13-3	inf
KRTAP29-1	inf
DDX11L1	inf
OR2L3	inf
OR2T27	inf
FBXO22-AS1	inf
RNASE8	inf
MIR3163	inf
LOC100289656	inf
FAM25B	inf
POU5F1P3	inf
C9orf169	inf
REXO1L2P	inf
LOC100272216	inf

FAM226B	inf
OR52E8	inf
KRTAP10-10	inf
FOXD4L3	inf
H1FX	inf
OR13H1	inf
FAM27B	inf
SNORA11	inf
SNORA7B	inf
SCARNA3	inf
PABPC1L2B	inf
LCE3A	inf
PRR21	inf
CXorf27	inf
DPM3	inf
IFNA7	inf
MRPL53	inf
OR7E37P	inf
PRR20E	inf
OR13C2	inf
OR8H3	inf
MIR660	inf
MIR502	inf
MIR3613	inf
MIR497	inf
SNORD115-10	inf
SNORD115-14	inf
SNORD115-19	inf
MIR4503	inf
MIR638	inf
MIR520E	inf
MIR941-3	inf
MIR941-2	inf
MIR5004	inf
MIR580	inf
MIR3960	inf
OR4M2	inf
LOC649352	inf
SF3B5	inf
SPANXC	inf
OR52N4	inf
OR4C13	inf
DPM3	inf
OR2T33	inf
OR2T35	inf
HIST1H4G	inf

OR8B2	inf
HIST1H3A	inf
CLDN17	inf
DUX4L4	inf
OR4F17	inf
SNHG9	inf
OR1E2	inf
MIR3652	inf
BACE1-AS	inf
WWOX	inf
SNORA4	inf
HIST1H3H	inf
MIR4767	inf
MIR4329	inf
MIR888	inf
MIR4302	inf
MIR4488	inf
MIR4294	inf
MIR4482-1	inf
MIR484	inf
MIR328	inf
SNORD114-8	inf
MIR296	inf
MIR3197	inf
SNORA22	inf
HIST1H4H	inf
MIR3692	inf
MIR711	inf
MIR4446	inf
MIR5703	inf
SNORD74	inf
MIR4260	inf
MIR4667	inf
MIR4668	inf
HNRNPCP5	inf
HCG14	inf
WFDC12	inf
OR2A4	inf
OR10H3	inf
OR11H1	inf
KRTAP13-4	inf
USP17L9P	inf
USP17L22	inf
OR5M10	inf
OR56A4	inf
FAM163B	inf

MIR221	inf
MIR106A	inf
MIR4725	inf
SNORD116-4	inf
SNORD116-16	inf
MIR329-2	inf
MIR320C1	inf
MIR5090	inf
MIR1236	inf
MIR324	inf
SNORD115-23	inf
MIR4307	inf
MIR527	inf
MIR4741	inf
MIR548F4	inf
MIR449A	inf
MIR3655	inf
MIR3921	inf
MIR942	inf
SNORA20	inf
SCARNA23	inf
MIR498	inf
TAS2R40	inf
HIST1H2AM	inf
CGB7	inf
OR10A2	inf
HIST1H3F	inf
TAS2R8	inf
OR11G2	inf
KRTAP8-1	inf
OR1D4	inf
FTH1P18	inf
OR1M1	inf
OR5M1	inf
OR6K6	inf
RPL23AP32	inf
OR10H5	inf
MIR5582	inf
OR5F1	inf
MIR4483	inf
SNORD49B	inf
MIR1910	inf
SNORD109A	inf
SNORD113-6	inf
MIR3201	inf
SNORD12C	inf

MIR4645	inf
MIR4270	inf
MIR4782	inf
SNORD38B	inf
MIR4711	inf
KRTAP19-8	inf
MIR450A2	inf
MIR1912	inf
RNU6-78P	inf
MIR670	inf
MIR548AR	inf
RNU6-59P	inf
MIR4726	inf
MIR4315-1	inf
MIR3178	inf
SNORD115-5	inf
SNORD115-10	inf
SNORD115-13	inf
SNORD115-25	inf
SNORD115-47	inf
MIR4312	inf
MIR3173	inf
SNORD113-7	inf
SNORD114-1	inf
MIR889	inf
MIR655	inf
MIR369	inf
MIR4710	inf
MIR4999	inf
MIR515-2	inf
MIR517C	inf
MIR1257	inf
MIR941-4	inf
MIR941-2	inf
MIR4652	inf
MIR592	inf
SNORD52	inf
MIR548AD	inf
SNORA47	inf
RNU6-69P	inf
MIR1301	inf
MIR216B	inf
MIR4444-1	inf
MIR1258	inf
MIR3129	inf
MIR4689	inf

MIR4695	inf
MIR137	inf
MIR2861	inf
SNORD36C	inf
MIR1322	inf
MIR3610	inf
OR51A4	inf
PPIAL4G	inf
MRPL41	inf
RPL23AP64	inf
DEFA9P	inf
OR1A1	inf
HIST1H3I	inf
MIR504	inf
MIR507	inf
MIR548X2	inf
MIR1287	inf
MIR378C	inf
MIR144	inf
MIR4737	inf
SNORD1B	inf
SNORD60	inf
MIR940	inf
SNORD116-9	inf
SNORD115-35	inf
SNORD115-10	inf
MIR4748	inf
MIR519C	inf
MIR519B	inf
MIR520C	inf
MIR521-2	inf
MIR593	inf
MIR9-2	inf
MIR1289-2	inf
MIR4267	inf
OR6C75	inf
MIR3169	inf
SNORD86	inf
MIR5707	inf
MIR194-1	inf
SPRR2A	inf
OR8D4	inf
C6orf226	inf
PI3	inf
KRTAP9-6	inf
HIST1H2BD	inf

LOC100240734	inf
KRTAP12-4	inf
MIR510	inf
MIR675	inf
SNORD114-6	inf
MIR376C	inf
MIR3192	inf
SNORD45C	inf
SNORD45B	inf
MIR4423	inf
SNAR-I	inf
NAT8B	inf
SNORA23	inf
LOC100996349	inf
MIR636	inf
MIR412	inf
SNORD19B	inf
MIR3663	inf
MIR489	inf
MIR1324	inf
SNORA5B	inf
HIST1H2BI	inf
MIR4500	inf
MIR3913-1	inf
MIR3913-2	inf
MIR1178	inf
MIR4694	inf
MIR4490	inf
MIRLET7A2	inf
MIR548AV	inf
MIR3158-2	inf
MIR146B	inf
MIR4484	inf
SNORD49A	inf
MIR4732	inf
MIR4728	inf
MIR4719	inf
SNORD64	inf
SNORD116-24	inf
SNORD115-4	inf
SNORD115-28	inf
MIR3175	inf
MIR4707	inf
MIR487B	inf
SNORA28	inf
SNORD83B	inf

RNU6-33P	inf
MIR4464	inf
MIR4442	inf
MIR4443	inf
MIR4262	inf
MIR4417	inf
MIR30C1	inf
SNORD46	inf
MIR557	inf
MIR1278	inf
MIR4671	inf
MIR601	inf
MIR4669	inf
MIR3689E	inf
MIR4292	inf
MIR4470	inf
MIR4661	inf
SNORA56	inf
SCARNA22	inf
MIR5094	inf
MIR342	inf
MIR647	inf
KRTAP19-5	inf
SNORD70	inf
MIR765	inf
MIR509-3	inf
SNORD115-45	inf
MIR3650	inf
MIR3675	inf
MIR1976	inf
IFNA6	inf
SNORA32	inf
MIR22	inf
MIR516B1	inf
SNORD119	inf
SNORD92	inf
HIST1H2BO	inf
MIR1260B	inf
MIR3183	inf
MIR4513	inf
SNORD59B	inf
MIR4708	inf
MIR3925	inf
MIR605	inf
SNORD115-40	inf
MIR1179	inf

MIR643	inf
SNORA72	inf
MIR555	inf
MIR1244-1	inf
MIR148B	inf
MIR603	inf
SNORD4B	inf
SNORD114-17	inf
MIR380	inf
MIR4745	inf
SNORD105B	inf
SNORD88B	inf
MIR519E	inf
MIR516A2	inf
MIR4325	inf
MIR3914-1	inf
MIR3914-2	inf
SNORD96A	inf
SNORD95	inf
MIR217	inf
MIR4436A	inf
MIR561	inf
MIR4255	inf
MIR3117	inf
MIR181B2	inf
MIR551B	inf
MIR3659	inf
MIR4478	inf
MIR2117	inf
SNORA15	inf
MIR511-1	inf
MIR493	inf
MIR1262	inf
MIR377	inf
MIR4775	inf
MIR1283-1	inf
MIR222	inf
MIR635	inf
SNORD116-21	inf
SNORD115-27	inf
SNORD58A	inf
MIR644A	inf
MIR3646	inf
SNORD63	inf
MIR146A	inf
SNORA16B	inf

MIR483	inf
SNORD116-27	inf
MIR5186	inf
MIR891B	inf
MIR508	inf
SNORD26	inf
MIR5579	inf
MIR553	inf
SNORA1	inf
MIR4263	inf
MIR3910-1	inf
MIR4298	inf
MIR4281	inf
SNORA62	inf
MIR3910-2	inf
KRTAP4-3	41,03
OR5J2	40,49
ACTRT2	35,64
OR52H1	23,22
OR5D18	23,22
SCARNA13	22,68
MIR597	22,67
TMEM256	22,41
MIR184	20,51
SNORA77	19,44
CLDN8	19,44
C6orf100	17,28
OR5H1	16,20
HBE1	14,85
OR51Q1	14,85
C16orf91	14,58
MC3R	14,31
FAM218A	14,04
SNORA67	14,04
OR1L4	13,50
OR10G7	13,23
MIR4784	12,96
OR10A5	12,42
RETN	12,31
H2AFX	12,24
OR13J1	12,15
TIMM8A	11,52
SPRR2G	11,11
FTH1P3	11,07
NEUROG1	10,80
PPBPP2	10,80

KRTAP5-11	10,80
HBA1	10,80
SPCS1	10,58
OR5C1	10,53
LOC100286922	10,08
OR4K15	10,08
MIR29B1	9,72
OR1E1	9,72
SPINT4	9,45
LCE1A	9,18
MIR892B	8,64
MIR1827	8,64
MIR4514	8,64
MIR516A1	8,64
MIR3664	8,64
SNORA58	8,64
OR5H15	8,64
OR9G4	8,28
HIST1H2AH	8,10
KTI12	8,10
OR51S1	7,78
POU5F1B	7,56
MIR376A1	7,56
NAP1L2	7,56
IGBP1P1	7,41
PCDHGA1	7,39
FAM26F	7,38
OR1G1	7,29
LOC1720	7,29
OR3A2	7,20
DEFB129	7,13
KRTAP13-1	7,13
OR6P1	7,02
GPHA2	7,02
SLC35G4	7,02
SNORD35A	7,02
MIR550A1	7,02
SNORD24	7,02
NAT8	6,79
BLOC1S4	6,75
OR4D11	6,75
OR5T1	6,75
OR4C16	6,70
LOC283352	6,69
LOC100270804	6,60
MAGEA6	6,48

MIR4637	6,48
SNORA2B	6,48
MIR4714	6,48
MIR885	6,48
MIR4677	6,48
SNORA74B	6,48
DEFB113	6,48
MIR326	6,48
MIR645	6,48
OR4A47	6,48
SCARNA4	6,48
TAAR3	6,24
TPRX1	6,21
ALYREF	6,21
SNORA9	6,21
MTRNR2L5	6,15
SNORA29	5,94
SNORD33	5,94
LOC392452	5,80
SULT1A4	5,76
LOC100128573	5,76
RPS14P3	5,67
LOC401127	5,67
ZNRF2P1	5,67
C16orf90	5,52
CYP2A6	5,49
RAD21-AS1	5,48
CGB8	5,40
OR5K4	5,40
MIR365A	5,40
MIR4257	5,40
TAS2R31	5,40
LOC100132781	5,40
FKBPL	5,40
OR10T2	5,40
TTC3P1	5,40
TAS2R16	5,40
LCE4A	5,40
SNORD85	5,40
MIR4763	5,40
GLUD1P7	5,40
MAGEB16	5,36
DBIL5P2	5,28
KMT2E-AS1	5,24
HUS1B	5,22
SNAR-A10	5,18

ZNF705B	5,18
SERF1A	5,13
SERF1B	5,13
OR5AC2	5,13
SCGB1D1	5,10
ATP6V0CP3	5,09
MKRN9P	5,08
TPD52L3	5,07
ACTRT1	4,99
OR2G6	4,99
FAM27E2	4,97
SAA3P	4,95
FFAR1	4,94
HIST1H4D	4,86
SNORD111B	4,86
MIR378D2	4,86
OR5L1	4,86
LOC254128	4,83
IGFL1	4,83
UGT2B15	4,80
CTAGE6	4,80
LOC100133985	4,78
TCEAL6	4,75
RBMXL2	4,68
MIR1-2	4,68
LOC613038	4,68
MIR4683	4,68
POU6F2-AS1	4,66
OR10W1	4,62
OR52E2	4,59
MIR657	4,53
FERD3L	4,50
IL36A	4,50
ZNF32-AS1	4,50
REG1P	4,48
HIST1H1D	4,45
LOC641515	4,45
MEIS3P1	4,44
YBX3P1	4,42
PWAR4	4,32
MIR3658	4,32
MIR4288	4,32
MIR4536-1	4,32
MIR4639	4,32
MIR4804	4,32
MIR524	4,32

DCAF12L1	4,32
PSENEN	4,32
KRTAP19-1	4,32
MIR4467	4,32
SCARNA5	4,32
MIR1224	4,32
MIR770	4,32
OR2H2	4,32
OR52B4	4,32
MIR2110	4,32
SNORD51	4,32
HIST4H4	4,32
P2RY13	4,27
LAMB2P1	4,25
CAPZA3	4,24
SLC6A10P	4,20
BTF3P11	4,20
RPL7A	4,18
MIR4308	4,17
HIST1H2BB	4,12
DEFB104B	4,10
MTRNR2L4	4,10
FAM181B	4,09
JUNB	4,08
IRGM	4,07
GOLGA8O	4,05
GPR89C	4,05
OR5K2	4,05
TAAR9	4,05
SPAG11B	4,05
GPR174	4,05
SMN2	4,05
SMN1	4,05
HIST1H2AA	4,05
OR5A2	4,05
CCR5	4,05
TNFRSF17	4,02
CXADRP2	3,99
IL37	3,98
OR2W5	3,98
LCE3B	3,96
MIR4738	3,96
CEMP1	3,96
GPR148	3,96
RPL23P8	3,96
LINC00982	3,95

CCL4	3,94
MRPL17	3,94
OR52A5	3,91
TSPYL6	3,89
KRTAP25-1	3,89
SDHAF1	3,89
OR5R1	3,89
ZNF135	3,88
KLRC2	3,87
HLA-DOA	3,87
LCE1B	3,86
IRS4	3,86
C8orf33	3,85
HOXA7	3,84
PWAR1	3,83
NKX2-3	3,83
SNORD56	3,78
MIR133A1	3,78
RPL21P44	3,78
OR2AK2	3,78
OR7D4	3,78
OR5W2	3,78
POU3F4	3,78
HIST1H2BH	3,78
MIR526A1	3,78
GOLGA8G	3,78
SARS	3,76
FOXF1	3,76
BEX4	3,73
OR5D13	3,73
CMA1	3,68
IL37	3,68
OR52I1	3,67
RBM5-AS1	3,67
GLUD2	3,66
LOC254128	3,66
KRTAP5-5	3,64
EID2	3,64
OR4K5	3,64
CST2	3,63
TPD52L3	3,63
RPL13P5	3,62
GAS1	3,61
OR52N2	3,60
KRTAP9-4	3,60
MIR548A1	3,60

REG3A	3,60
REG3A	3,60
C19orf73	3,60
MIR4656	3,60
SNORD72	3,60
UQCRQ	3,59
NPY5R	3,58
KRT6C	3,58
DLG5-AS1	3,57
HULC	3,56
LINC00588	3,55
FAM229A	3,55
CDRT15P1	3,51
ERVV-1	3,51
NCBP2-AS2	3,51
OR8U8	3,51
MAGEB1	3,50
LOC100130849	3,49
SPANXN3	3,49
OR10H4	3,48
AKR1C2	3,48
PCDHA9	3,47
LINC00662	3,46
OR2G3	3,46
ZNF830	3,46
SPRR1B	3,45
S100A8	3,42
TOB1-AS1	3,42
NDUFA2	3,41
HLA-DQB2	3,39
OR1N2	3,38
OR7G1	3,37
ILF3-AS1	3,36
OR6M1	3,35
DARC	3,34
UQCR10	3,34
AQP12B	3,32
ZNF733P	3,30
RGPD2	3,30
CD1E	3,27
NDUFB4	3,24
KRTAP22-2	3,24
MIR149	3,24
PRAMEF1	3,24
BRWD1-IT2	3,24
TCP11X2	3,24

MIR202	3,24
MIR4436B1	3,24
MIR1304	3,24
MIR4685	3,24
MIR1539	3,24
MIR4529	3,24
SNORD117	3,24
MIR4660	3,24
FAM27L	3,24
DNAJB7	3,24
MIR3916	3,24
LOC100303749	3,24
MIR766	3,24
MIR617	3,24
SNORD83A	3,24
MIR196B	3,24
MIR4275	3,24
SNAR-H	3,24
MIR4290	3,24
OR5M9	3,24
SNORA59A	3,24
DTX3	3,23
PDX1	3,21
PLA2G2E	3,21
WBP11P1	3,20
ADRA2A	3,20
MID1IP1	3,20
OR4K14	3,19
IL1RL1	3,17
DKK1	3,15
IFNA2	3,15
TMEM155	3,13
KIR3DL2	3,13
OR6C3	3,12
PCDHGB3	3,11
OR56B4	3,11
HIST1H3G	3,10
CBWD3	3,09
CBWD5	3,09
KRT34	3,08
TFF2	3,06
APOE	3,06
ULK4P1	3,06
OR10G9	3,06
C17orf97	3,06
IER2	3,06

LINC00173	3,06
NCF1B	3,05
PURG	3,05
MAGEE2	3,04
PYDC1	3,04
COX8C	3,02
CAMP	3,02
MIR136	3,02
MIR5691	3,02
HOXB9	3,02
TAF10	3,01
APOC1P1	3,00
TMEM257	3,00
HES3	3,00
LY6G6F	2,99
OR10R2	2,97
MIR3684	2,97
PNMA5	2,95
OR4Q3	2,95
TIMM8A	2,95
PDZD11	2,94
CARD18	2,93
HIST1H1B	2,93
GPR151	2,92
LHB	2,91
RNASE3	2,91
C10orf40	2,90
OR9Q2	2,90
C3orf36	2,89
ASPRV1	2,88
MIR4672	2,88
SPIN2A	2,88
GPR45	2,88
MIR5193	2,88
MIR602	2,88
KRTAP20-3	2,88
PRR14	2,87
LOC113230	2,86
CSN3	2,86
AQP7P3	2,86
OR5B12	2,85
IFNG	2,85
OR3A4P	2,84
OR14J1	2,83
OR13C5	2,83
FGFBP1	2,83

TISP43	2,83
TISP43	2,83
LOC646743	2,83
PCDHA6	2,82
NLGN4Y	2,81
LOC100287534	2,81
KIR2DL4	2,81
NLGN4Y	2,81
NLGN4Y	2,81
NLGN4Y	2,81
H3F3C	2,81
ZNF32-AS2	2,81
GPX8	2,80
TEX38	2,79
NKX2-8	2,78
HLA-DRB5	2,78
ADAM21P1	2,76
OR2F2	2,76
OR5V1	2,76
LINC00516	2,75
LINC00628	2,75
EGFL8	2,74
ABCC6	2,73
LYZL6	2,73
LOC286437	2,73
CCL1	2,72
LOC731223	2,72
RBMXL3	2,72
NANOG	2,72
GNGT2	2,71
APOA1	2,70
BTNL8	2,70
INGX	2,70
OR7C1	2,70
SCGB1B2P	2,70
SLC25A52	2,70
HIST1H3C	2,70
LCE1F	2,70
HOXD4	2,70
OR52L1	2,70
RPL41	2,70
LOC100289511	2,70
RPL29P2	2,70
AOAH-IT1	2,70
MID1IP1	2,70
SPIN2B	2,70

MIR629	2,70
OR10H1	2,70
MIR4316	2,70
MIR4648	2,70
SNORA5A	2,70
MIR4773-2	2,70
SNORD30	2,70
MIR4258	2,70
BASP1P1	2,70
SCARNA17	2,70
UTY	2,70
KRTAP21-3	2,70
UTY	2,70
FAM25B	2,70
FAM25G	2,70
GOLGA8F	2,70
SNRNP27	2,69
PSMB10	2,68
OR2AT4	2,67
SLURP1	2,67
TRAPPC1	2,67
NAIP	2,66
NAIP	2,66
AA06	2,66
ZNF549	2,65
ZNF135	2,65
DIO3OS	2,65
TARDBP	2,64
UBE2M	2,64
FAM133CP	2,64
ATP5G3	2,64
ATP5G3	2,64
PEX16	2,64
RBAKDN	2,63
HMGN3-AS1	2,63
LOC284933	2,63
PGLYRP2	2,62
HLA-DQA2	2,62
CD3EAP	2,61
DDX11L2	2,61
C15orf40	2,61
SRP14	2,60
EPC1	2,60
HOXD-AS2	2,60
MIR3618	2,59
OR10A4	2,59

SNORA71A	2,59
KRTAP9-9	2,59
CCDC121	2,59
MKRN3	2,59
NHLH1	2,57
OR8I2	2,57
OR52R1	2,56
MIR96	2,56
MIR3605	2,56
MIR3689A	2,56
ODF3	2,56
RPL29	2,56
NAP1L3	2,55
CNTD2	2,55
ZNF705A	2,55
IGFL4	2,55
TRAPPC1	2,54
RFPL4A	2,54
PPP1R3G	2,53
MIR2392	2,52
MIR877	2,52
MT1X	2,52
RIMBP3	2,52
MIR183	2,52
IFITM4P	2,52
OR8U1	2,52
MIR4786	2,52
CGB2	2,52
CPS1-IT1	2,52
MIR550A2	2,52
ANXA2P1	2,52
ADORA3	2,51
AZGP1P1	2,51
ID4	2,51
HIF1A-AS2	2,51
MIR4492	2,50
SH3GL1P2	2,49
CXCR5	2,49
OR52E4	2,48
XCL2	2,48
YRDC	2,48
C12orf74	2,48
MAGEA10	2,46
HAMP	2,45
OR7E2P	2,45
PDZK1IP1	2,45

STATH	2,45
LOC100506384	2,45
TXNDC17	2,45
TSN	2,44
GDF3	2,44
TMCO2	2,44
CITED4	2,44
FAM156B	2,43
FAM156A	2,43
RBM12B-AS1	2,43
LCE3E	2,43
SNORA70C	2,43
UQCRHL	2,43
OR5AN1	2,43
SERP1	2,43
POU4F1	2,42
GNGT2	2,42
C6orf141	2,42
CXCL11	2,42
HIST2H2BE	2,42
TSPO2	2,41
CUTA	2,41
DOCK9-AS2	2,41
MDH2	2,41
NPIPB3	2,40
SPRR2F	2,40
GAGE2C	2,40
SHISA9	2,40
CCL7	2,39
FOXC1	2,39
NPTN-IT1	2,38
SIKE1	2,38
GSX1	2,38
OR4D2	2,38
LOC100288911	2,38
OR12D2	2,38
KRTAP10-8	2,38
NKRF	2,37
GNGT2	2,37
CCDC121	2,37
TAPSAR1	2,36
AOC3	2,36
UTP3	2,36
SNAR-E	2,36
KBTBD13	2,36
LRRC30	2,36

PCED1B-AS1	2,36
C17orf112	2,36
DKFZp434J0226	2,36
MYF6	2,36
PNMA5	2,34
MPHOSPH10	2,34
IRX2	2,34
MRGPRX1	2,34
TSHB	2,33
LOC100129520	2,33
RIPPLY1	2,33
CDV3	2,32
PFN1	2,32
PCDHA1	2,32
BCAS2	2,32
ZNF224	2,32
GSPT2	2,32
CDV3	2,31
NPBWR2	2,31
HOXC-AS1	2,31
LOC100288778	2,31
LOC100506076	2,31
GATA3-AS1	2,31
TMEM86A	2,31
ELOVL3	2,31
APEH	2,31
PMAIP1	2,31
CH25H	2,31
KIR3DL1	2,31
LINC00173	2,31
MIR4276	2,30
PMS2L2	2,30
LOC286359	2,30
OR10A6	2,29
LINC00852	2,29
GHSR	2,29
ZNRF4	2,29
C1QTNF4	2,28
CETN4P	2,28
ECSCR	2,28
SAMD15	2,28
PITX2	2,28
ASPHD1	2,28
MIR612	2,28
LOC100506328	2,28
KLK12	2,27

OR6C6	2,27
LOC100130451	2,27
POU4F3	2,27
SEMG1	2,27
CKMT1B	2,27
MIR148A	2,27
LDHAL6B	2,26
TSPO2	2,26
FAM138D	2,26
FAM187B	2,26
TTC36	2,26
RNF223	2,26
C3orf72	2,25
GPR52	2,25
PRSS2	2,25
RLN2	2,25
PGBD4	2,25
OR6N1	2,25
RSPO1	2,24
ZNF256	2,24
C17orf47	2,24
FOXR2	2,24
TAAR6	2,24
HOXD11	2,24
LINC00661	2,23
DNAJC4	2,23
KCNMB1	2,23
HPS6	2,23
HOXB2	2,23
MAGEB18	2,22
RPL13AP3	2,22
P2RY4	2,22
KRT79	2,22
SPATA8-AS1	2,22
OMP	2,21
KRT33B	2,21
CLK2P	2,21
RPL37A	2,21
SUMO1P3	2,21
CCDC85B	2,20
UBQLNL	2,20
TLR9	2,20
GLYCAM1	2,19
CLDND1	2,19
GPX6	2,19
RAET1E	2,19

TPI1	2,19
PCDHB1	2,19
PET100	2,19
RHOV	2,19
HLA-G	2,18
LILRB2	2,18
LILRB2	2,18
LILRB2	2,18
LILRB2	2,18
NXF3	2,18
C4orf33	2,18
SPEF1	2,18
SNORA49	2,16
MIR139	2,16
SNORD4A	2,16
SNORD34	2,16
MIRLET7C	2,16
HBG2	2,16
MIR224	2,16
MIR5100	2,16
MIR190A	2,16
MIR4309	2,16
SBSN	2,16
LOC100289092	2,16
SNORD69	2,16
MIR4273	2,16
IFNA8	2,16
LOC729080	2,16
LOC284379	2,16
MIR24-2	2,16
MOBP	2,16
MIR4733	2,16
MIR203	2,16
MIR3545	2,16
MIR432	2,16
CCDC163P	2,16
GNLY	2,16
CRB3	2,16
ZNF654	2,16
MIR23B	2,16
SSNA1	2,16
MIR15B	2,16
HILS1	2,16
SORCS3-AS1	2,16
LINC00957	2,16
LOC441455	2,16

MIR3187	2,16
OR9G1	2,16
SEMG2	2,16
RNASE13	2,16
MMP23A	2,16
FAM72A	2,16
LOC643486	2,16
SPACA5	2,16
HGD	2,16
GOLGA8EP	2,16
NEUROG3	2,16
USP17L4	2,16
CDRT7	2,16
MIR1264	2,16
MIR891A	2,16
RNU6-67P	2,16
MIR506	2,16
MIR4705	2,16
MIR1228	2,16
MIR1469	2,16
MIR381	2,16
SNORD110	2,16
MIR4468	2,16
MIR153-1	2,16
MIR4286	2,16
OR2T3	2,16
OR2T34	2,16
LOC729737	2,16
LOC100652736	2,16
HBZ	2,16
TBC1D3H	2,16
VN1R10P	2,16
FAM225B	2,16
SNORD116-18	2,16
MIR574	2,16
CD3E	2,15
MRPL9	2,15
C4orf33	2,15
FAM87A	2,15
PSMB8	2,15
PLA2G2D	2,14
ASAH2B	2,14
GADD45GIP1	2,14
FOXP3	2,14
ZNF737	2,14
MTCP1	2,13

SMR3B	2,13
TCONS_00024492	2,13
CTSLP8	2,13
BOLA3-AS1	2,13
ALKBH4	2,13
C11orf91	2,12
S1PR5	2,12
JPH3	2,12
PCDHA10	2,12
C16orf13	2,12
CTGF	2,12
PMCH	2,12
MRPL11	2,12
FAM71E1	2,11
OR2K2	2,11
CKMT1A	2,11
NKX6-1	2,11
RNF26	2,10
SPACA4	2,10
OR52M1	2,10
SCGB2A2	2,10
OR13C9	2,10
ZNF80	2,10
BTG1	2,09
LOC440905	2,09
FAM72B	2,09
C3orf80	2,09
ATP6V1F	2,09
APOBEC3C	2,09
TMEM31	2,09
CITED1	2,09
RPS12	2,09
CHPF	2,09
LOC100996758	2,09
ALB	2,09
FOXD2-AS1	2,09
HMGB4	2,08
SDR39U1	2,08
ASCL2	2,08
LOC653501	2,08
LOC440896	2,08
OR56A1	2,08
SYCE1	2,08
HCRT	2,07
KLHDC7A	2,07
C11orf1	2,07

PRNT	2,07
KRT14	2,07
BOD1L2	2,07
MED18	2,07
PGA3	2,07
CXorf40A	2,06
MSGN1	2,06
BCL2	2,06
MRPL16	2,06
FGF8	2,06
ZFP92	2,05
DRD1	2,05
C9orf66	2,05
CLEC10A	2,05
TSPAN6	2,05
LINC00421	2,05
PAGE2B	2,04
TWF1	2,04
GNRH2	2,04
CNPPD1	2,04
GALR3	2,04
FKSG29	2,04
OR7E12P	2,04
ZNF619	2,04
HLA-DOB	2,04
LIM2	2,04
FLJ26245	2,04
PAGR1	2,03
TMEM88	2,03
OR10Z1	2,03
ANXA2P3	2,02
RNF5	2,02
AGAP4	2,02
TREM1	2,02
OR4K1	2,02
GUSBP9	2,02
SKIV2L	2,02
LINC00893	2,02
KRTDAP	2,02
PTHLH	2,02
FOXS1	2,02
HINFP	2,02
ESRP2	2,02
TCF15	2,02
SPINK14	2,01
PKI55	2,01

PLA2G12A	2,01
CD207	2,01
NEFM	2,01
AMT	2,01
C1GALT1C1	2,01
CLECL1	2,01
RING1	2,00
IL10RA	2,00
IMP3	0,50
RCOR2	0,50
CALCA	0,50
SNORD114-20	0,50
OR6C65	0,50
SCGB2B3P	0,50
HYI	0,50
ZBTB3	0,50
TRIM49B	0,50
GJD2	0,50
LHX1	0,50
LOC100268168	0,50
CPT1B	0,50
LAGE3	0,50
NR1I3	0,50
CDCA3	0,50
sept-05	0,50
GLIS3-AS1	0,50
OR5AS1	0,49
MCL1	0,49
LOC100268168	0,49
LOC100288069	0,49
ZXDB	0,49
C7orf49	0,49
LYPD5	0,49
COMTD1	0,49
AQP12A	0,49
GH2	0,49
ATP7A	0,49
GSN-AS1	0,49
STAG3L3	0,49
CAMK2N2	0,49
OR2T1	0,49
HMX3	0,49
ANXA8	0,49
DUX4	0,49
TRIM73	0,49
TRIM74	0,49

KRTAP4-6	0,49
C4A	0,49
C4B_2	0,49
OR10Q1	0,49
PIK3R1	0,49
OXLD1	0,49
NKAPL	0,49
RPL15	0,49
MRP63	0,49
CXCR2	0,49
DLX1	0,49
RDH14	0,49
C8orf86	0,49
KLHL38	0,49
BLOC1S1	0,49
TLR8	0,49
LOC653712	0,49
DNASE1L2	0,49
CDK4	0,49
LRRC2-AS1	0,49
NRG1-IT3	0,49
CXorf64	0,49
MIR4285	0,49
MIR4269	0,49
PRR24	0,49
ATXN3L	0,49
FAM115A	0,49
NPW	0,48
CHKB-CPT1B	0,48
SPDYE4	0,48
HBBP1	0,48
ARF6	0,48
MIR3065	0,48
MIR338	0,48
PIP5KL1	0,48
SNRPD2	0,48
IL2RG	0,48
LPP-AS2	0,48
PINLYP	0,48
TIGD2	0,48
CXCR4	0,48
RDH8	0,48
HAGHL	0,48
CD300LD	0,48
RPL15	0,48
CCL15	0,48

C17orf77	0,48
HMGB1	0,48
MORN2	0,48
CYB561D1	0,48
CYB561D1	0,48
SNORA68	0,48
MFAP2	0,48
GH1	0,48
CFL2	0,48
MS4A6E	0,48
MIR520F	0,48
MIR921	0,48
WNT1	0,48
RAB3A	0,48
PCDHGB5	0,48
FOXC2	0,48
HSPB3	0,48
RNF224	0,48
MOSPD3	0,48
RGS1	0,48
RPL15	0,48
TAAR2	0,48
OBP2B	0,48
ZNF296	0,48
BST2	0,48
C10orf12	0,48
B4GALT3	0,48
GALT	0,48
HOXC-AS2	0,48
RPL15	0,48
SOX10	0,48
DEFB136	0,48
RAN	0,48
SPRR2D	0,48
NEURL2	0,47
CHRNG	0,47
PART1	0,47
MFAP2	0,47
TRERNA1	0,47
TFDP3	0,47
RAET1L	0,47
TMEM121	0,47
ATF3	0,47
PTPRCAP	0,47
LOC284578	0,47
TMEM52	0,47

HOXC6	0,47
sept-12	0,47
NDUFA6	0,47
SUPT20HL2	0,47
DEFB127	0,47
FAM74A3	0,47
HRG	0,47
CALML5	0,47
MIR5189	0,47
ARL6IP4	0,47
POLR1C	0,47
TNXB	0,47
C1orf122	0,47
BPIFA4P	0,47
ASIC1	0,47
CPT1B	0,47
C7orf25	0,46
MAF1	0,46
LINC01015	0,46
SNORD91A	0,46
LRRC37A2	0,46
CDC14C	0,46
RNF181	0,46
FAM115C	0,46
MESP1	0,46
ARL17A	0,46
TK1	0,46
LRP5L	0,46
MRPL10	0,46
HCG22	0,46
C2orf68	0,46
SS18L2	0,46
GAMT	0,46
NXNL2	0,46
LTA	0,46
LOC100506795	0,46
APOBEC3H	0,46
IL1F10	0,46
TIGD7	0,46
LOC400685	0,46
DGCR10	0,46
RNF215	0,46
LOC100131094	0,46
CCDC177	0,46
PABPC1L2A	0,46
GJA10	0,45

HEIH	0,45
USE1	0,45
RNASEK	0,45
TCAP	0,45
EGR4	0,45
OR13F1	0,45
KGFLP1	0,45
SNORA63	0,45
IL17C	0,45
POLR2L	0,45
NBPF24	0,45
RPS15AP10	0,45
NBPF11	0,45
NBPF24	0,45
OR13C8	0,45
MT1DP	0,45
CCDC166	0,45
LILRB4	0,45
GCHFR	0,45
SPZ1	0,45
FLJ44313	0,45
RHOB	0,45
SHISA4	0,45
SHISA4	0,45
C1orf64	0,44
RNF103	0,44
NANOS2	0,44
C1orf180	0,44
FBXL6	0,44
KBTBD6	0,44
PLA2G7	0,44
IER5	0,44
MT1JP	0,44
LOC283070	0,44
MED21	0,44
SERTAD4-AS1	0,44
ENPP5	0,44
LTC4S	0,44
LOC100287632	0,44
PIGY	0,44
RPP21	0,44
ORM1	0,44
VSX1	0,44
LOC100287177	0,44
SLC10A3	0,44
C4orf48	0,44

BET1L	0,44
SNTN	0,44
TINF2	0,44
PHGR1	0,43
KCND1	0,43
MESTIT1	0,43
CENPT	0,43
SNORD114-12	0,43
HLA-DRB6	0,43
EPHX3	0,43
MIR5089	0,43
MYADML	0,43
NKX2-2	0,43
SNORD10	0,43
MIR188	0,43
MIR518F	0,43
MIR4759	0,43
MIR1297	0,43
MIR4681	0,43
SNORD18C	0,43
MIR4519	0,43
SNORD101	0,43
SNORA66	0,43
OR2W3	0,43
DPRXP4	0,43
LOC100129917	0,43
POC1B-GALNT4	0,43
FAM92A1P2	0,43
KPNA2	0,43
C22orf42	0,43
LOC146481	0,43
LOC348761	0,43
PLA2G7	0,43
CCL14	0,43
LINC00161	0,43
DLEU2L	0,43
LINC00092	0,43
TMEM211	0,43
SLC7A6OS	0,43
GTSE1-AS1	0,43
SCAND1	0,42
IFITM3	0,42
LINC00657	0,42
GSTM4	0,42
CCDC153	0,42
IFNA22P	0,42

ATOH7	0,42
PRSS8	0,42
APOBEC3H	0,42
NAT14	0,42
OR5D14	0,42
NHLRC4	0,42
TSPY26P	0,42
SLC9A9-AS1	0,42
TUBB2B	0,42
MAD2L1	0,42
PARD6A	0,42
POLR2J	0,42
TUBB	0,42
RAET1G	0,42
FJX1	0,42
NRARP	0,42
ARMCX3	0,42
MGC34796	0,42
MGC16025	0,42
LGALS1	0,42
FAM35BP	0,42
TRIM49C	0,42
TPSAB1	0,42
OR7D2	0,42
OR2A5	0,42
CTAGE10P	0,42
FAM86EP	0,41
UPK1A-AS1	0,41
FEZ1	0,41
EPHX3	0,41
LINC00577	0,41
CRHR1-IT1	0,41
ZBED2	0,41
KRT37	0,41
LOC645434	0,41
OXCT2	0,41
GDNF-AS1	0,41
IRG1	0,41
NUTM2D	0,41
MAGEE1	0,41
TREML4	0,41
FAM83E	0,41
FZD10	0,41
LOC646903	0,41
APOA2	0,41
MIR589	0,41

LINC00618	0,41
CYP1A1	0,41
NUDT8	0,41
NUDT8	0,41
LINC01023	0,41
OR52N5	0,41
HIST3H3	0,41
SNORA71D	0,41
ARMCX3	0,41
MIR2682	0,41
SOX3	0,41
LGALS9B	0,40
SPAG11A	0,40
MIR4793	0,40
MIR556	0,40
MIR4679-2	0,40
MIR4679-1	0,40
SNORA69	0,40
HDGFL1	0,40
PRM3	0,40
OR4K2	0,40
TMEM89	0,40
CREM	0,40
KRT8P41	0,40
RTP3	0,40
LRRC26	0,40
GOLGA8I	0,40
SPATA25	0,40
PTPN20A	0,40
LOC100287534	0,40
PTPN20B	0,40
KIR2DL4	0,40
LOC339524	0,40
SOX21	0,40
DALRD3	0,40
TMEM185B	0,40
CCL18	0,40
NUBP2	0,40
CHKB	0,39
SPATA8	0,39
PIP5K1P1	0,39
GLTPD2	0,39
INSM1	0,39
FAM90A25P	0,39
RRAS	0,39
SULT1A1	0,39

CCDC41-AS1	0,39
GAMT	0,39
LOC100507387	0,39
SIX6	0,39
PRKXP1	0,39
CHMP4A	0,39
DEFB121	0,39
OR2AE1	0,39
CRYGS	0,39
NFE2	0,39
RAB40AL	0,39
FOXD4L1	0,39
LCE2C	0,39
LOC389332	0,39
IFIT3	0,39
PTRHD1	0,39
OR6B2	0,39
RPLP2	0,38
CLDN5	0,38
MPDU1	0,38
ID2	0,38
CHAD	0,38
ACR	0,38
PABPC4L	0,38
DSCAM-AS1	0,38
DSCAM-AS1	0,38
TNP2	0,38
PI16	0,38
GCNT4	0,38
YY2	0,38
SOX14	0,38
LOC401242	0,38
OR4D9	0,38
MEA1	0,38
GATM-AS1	0,38
OR52D1	0,38
KRTAP5-6	0,38
APEX1	0,38
PCDHB17	0,37
MIR126	0,37
ZNF581	0,37
PRDX5	0,37
RPS21	0,37
PITPNA-AS1	0,37
CORT	0,37
HOXD12	0,37

C9orf131	0,37
MSTO1	0,37
FANCD2OS	0,37
C9orf172	0,37
FNDC8	0,37
LINC00515	0,37
SNRPD2P2	0,37
LOC100996255	0,37
GAGE2A	0,37
PCDHGA8	0,37
KBTBD7	0,37
C4orf48	0,37
MAGEB5	0,37
GALNT4	0,37
ZP1	0,37
WBP1	0,37
CST6	0,37
LAMA4	0,37
ACY3	0,37
APOC4	0,37
LOC100128361	0,37
RAB6C	0,36
EIF1AD	0,36
RHOXF2B	0,36
RHOXF2	0,36
SNORD116-19	0,36
MIR1306	0,36
MIR4779	0,36
MIR199B	0,36
GOLGA6B	0,36
OR8H2	0,36
MIR4727	0,36
MIR3940	0,36
MIRLET7G	0,36
MIR4666A	0,36
MIR583	0,36
IFNA10	0,36
IFNL2	0,36
LOC613037	0,36
LOC100130348	0,36
MIR130A	0,36
MIR1276	0,36
LOC100505978	0,36
GPR84	0,36
MIR548J	0,36
MIR3130-2	0,36

MIR935	0,36
C1orf229	0,36
SNORD17	0,36
PRAF2	0,36
ZNHIT2	0,36
SNORA5C	0,36
C9orf131	0,36
ANKRD63	0,36
PRR9	0,36
MIR4297	0,36
SNORD81	0,36
SNORA74A	0,36
SNORD116-8	0,36
SNORD116-29	0,36
MIR3150B	0,36
KRTAP17-1	0,36
ATP5EP2	0,36
OR13C3	0,36
ISG15	0,35
PRM2	0,35
TSTD1	0,35
SRSF1	0,35
MAS1L	0,35
KCNA10	0,35
DPY19L2P3	0,35
MRGPRD	0,35
NXNL1	0,35
LOC149086	0,35
TPP1	0,35
OTOR	0,35
CDK16	0,35
LOC541473	0,35
SCGB1C1	0,35
IFNK	0,35
LOC143666	0,35
OR2A25	0,35
BLOC1S1	0,34
CCL3	0,34
PCDHB9	0,34
MMP9	0,34
MSTO2P	0,34
TMEM14E	0,34
FAM122A	0,34
MIR431	0,34
ULBP3	0,34
HTATSF1P2	0,34

SNORD114-24	0,34
GPX4	0,34
NAP1L6	0,34
PPP3R2	0,34
HOXA6	0,34
PNRC1	0,34
TTC9B	0,34
HIST2H2BA	0,34
LINC00889	0,34
MRPS12	0,34
ATP6V1G2	0,34
UBQLN3	0,34
MRPL34	0,34
PTH2	0,34
GPX4	0,34
ISM1-AS1	0,34
TMEM88B	0,34
OR1K1	0,34
ICAM4	0,33
LOC339524	0,33
GABARAPL3	0,33
SLC39A7	0,33
WHAMMP1	0,33
FABP1	0,33
LINC00634	0,33
PNN	0,33
SLX1B	0,33
OR2AG2	0,33
DANCR	0,33
KCNA2	0,33
HIST1H2BL	0,32
MIR133A2	0,32
MIRLET7F1	0,32
TAS2R14	0,32
MIR34C	0,32
MIR4296	0,32
MIR193A	0,32
C2CD4A	0,32
YAF2	0,32
FOXH1	0,32
CYB561D2	0,32
NACAP1	0,32
C12orf68	0,32
OR52K1	0,32
KRTAP12-2	0,32
LOC641367	0,32

MIR411	0,32
ANXA8L1	0,32
ANXA8L2	0,32
TRMT10C	0,31
GP1BA	0,31
SNORD114-23	0,31
GPR119	0,31
GAS6-AS2	0,31
MIR31	0,31
FAM90A7P	0,31
LOC100287846	0,31
C16orf86	0,31
MIR548U	0,31
MIR7-2	0,31
PRAC1	0,31
YPEL4	0,31
LILRA6	0,31
CCL21	0,31
LOC440117	0,31
C9orf142	0,30
OR10H2	0,30
SPATA33	0,30
MIR3126	0,30
LOC286297	0,30
DKFZp779M0652	0,30
LOC100270746	0,30
STH	0,30
MDK	0,30
MDK	0,30
MDK	0,30
MDK	0,30
OR4A15	0,30
ANXA2R	0,30
PPP1R3E	0,30
ENSA	0,30
SNORD105	0,29
OR56B1	0,29
MAN1B1-AS1	0,29
SNORA27	0,29
FDX1L	0,29
ANKRD36BP1	0,29
MRPS12	0,29
LOC728752	0,29
LOC653653	0,29
MIR3141	0,29
S100A6	0,29

RXFP4	0,29
ARL14	0,29
LOC101059948	0,28
OR5P2	0,28
SNHG5	0,28
TPRKB	0,28
REP15	0,28
LOC100505812	0,28
PCDHGA3	0,28
BIN3-IT1	0,28
GJB5	0,28
FAM195B	0,28
OR2G2	0,28
NGFRAP1	0,27
THAP11	0,27
SNORD116-23	0,27
MIR495	0,27
MIR5088	0,27
MIR1234	0,27
TOPORS-AS1	0,27
SNORD16	0,27
MIR4790	0,27
ZNRF2P2	0,27
H2BFXP	0,27
MIR3165	0,27
SLC35G6	0,27
EIF3IP1	0,27
LCE1C	0,27
HLA-DQA1	0,27
MIR661	0,27
SNORD114-31	0,27
SNORA8	0,27
MIR662	0,27
LY6G5B	0,27
ANP32C	0,27
MIR195	0,27
HOXC12	0,27
SAP25	0,27
OR5D16	0,26
HSPA1B	0,26
TRIM51HP	0,26
GATSL1	0,26
GATSL2	0,26
OR7E14P	0,26
TEX21P	0,26
SNORD114-21	0,26

OR2B11	0,26
GOLGA6D	0,26
MIR3976	0,26
LIPT2	0,26
TOP1P1	0,26
IFNL1	0,26
OR6Y1	0,26
UPK3BL	0,25
TAS2R30	0,25
OR4N2	0,25
FAU	0,25
LCE5A	0,25
NUTM2A	0,25
SBDS	0,25
ZNF524	0,25
SNORD116-2	0,25
BARX1	0,25
CNFN	0,25
IFNA16	0,25
LOC390660	0,25
OR8H1	0,25
OR9A2	0,25
OR14A16	0,25
FAM106CP	0,25
SLC25A2	0,25
LOC392196	0,25
DIO3	0,25
MANEA-AS1	0,25
LOC440925	0,24
NGFRAP1	0,24
LOC101241902	0,24
NPB	0,24
KRTAP20-1	0,24
SNORA31	0,24
LOC100272217	0,24
ATF4	0,24
C11orf86	0,24
SNORD87	0,24
CST1	0,24
DEFB133	0,24
OR10J1	0,24
FTO-IT1	0,24
OR1J2	0,23
LOC729020	0,23
MIR4684	0,23
CDK5R2	0,23

DCAF8L2	0,23
CCL24	0,23
TMEM167B	0,23
MARK2P9	0,23
ZFP62	0,23
OR10G3	0,23
FRMD8P1	0,23
EDDM3A	0,23
COMMD4	0,22
MT2A	0,22
PTPN20A	0,22
LOC100288748	0,22
SNORD6	0,22
MIR558	0,22
TMSB10	0,22
SNORD114-9	0,22
SNORD19	0,22
MIR4265	0,22
MIR27B	0,22
LOC100132356	0,22
SCARNA9L	0,22
OR2M5	0,22
SNORA34	0,22
TXNDC2	0,22
MIR19B1	0,22
HELT	0,21
PRR22	0,21
CLEC3A	0,21
GTSF1L	0,20
SLC25A21-AS1	0,20
IFITM1	0,20
SNORD116-13	0,20
IFNA21	0,20
SNORD1A	0,20
HTR1B	0,20
PFDN6	0,20
PFDN6	0,20
MIR1270-1	0,20
TCEAL8	0,20
D2HGDH	0,20
C12orf57	0,19
C5AR2	0,19
CLEC18C	0,19
DBIL5P	0,19
OR10A3	0,19
HCFC1R1	0,19

TDRG1	0,19
OR5B2	0,18
LILRB4	0,18
MIR1305	0,18
MIR5187	0,18
MIR5190	0,18
SNORA33	0,18
SCARNA1	0,18
OR4C11	0,18
SNORD35B	0,18
LCE3D	0,18
RPS27	0,18
SNORD97	0,18
SNORA70B	0,18
SIGMAR1	0,18
GPR31	0,17
OVCA2	0,17
SOX4	0,17
KRTAP10-6	0,17
HBB	0,17
UCN	0,17
OR51F1	0,17
PPCS	0,17
ACTL7B	0,17
LOC84931	0,17
MIR1247	0,17
HS6ST2-AS1	0,17
OR8D1	0,17
FOXD4	0,17
MIR181B1	0,17
LOC100506314	0,16
SCNM1	0,16
TUBB4B	0,16
MAGEF1	0,16
OR5T3	0,16
EGOT	0,16
OR1C1	0,15
OR1S2	0,15
SPEM1	0,15
OR2B3	0,15
LOC100130075	0,15
SNORA17	0,15
GAGE2A	0,15
GAGE2C	0,15
GAGE2E	0,15
GAGE8	0,15

ARMCX6	0,14
OR8K3	0,14
GADD45G	0,14
GJB1	0,14
POU3F1	0,14
MAGOH2	0,14
PHLDA2	0,14
MIR138-1	0,14
HIST1H2AC	0,14
TOPORS-AS1	0,14
MIR193B	0,14
LOC407835	0,13
SNORD89	0,13
NRN1L	0,13
AHSP	0,13
SLC18A3	0,13
MIR339	0,13
FAM178A	0,13
OR5AK4P	0,13
MIR4460	0,13
TMEM133	0,12
MIR21	0,12
MIR4724	0,12
FAM127C	0,12
MIR4757	0,12
SNORD94	0,12
OR7E24	0,12
DRAP1	0,12
SNORD32A	0,12
UPK2	0,12
LTB4R2	0,11
OR2AP1	0,11
MIR5687	0,11
MIR190B	0,11
CCNO	0,11
FAM71C	0,11
MGC45922	0,10
NBPF6	0,10
C9orf135-AS1	0,10
MIR367	0,10
MT1HL1	0,10
HIST2H2AC	0,10
SSTR5	0,09
C17orf100	0,09
MIR1291	0,09
LOC284023	0,09

OR7G2	0,09
RPPH1	0,08
OR51I1	0,08
PCDHGB4	0,08
MT1M	0,07
OR8K1	0,07
MIR218-2	0,07
HAND1	0,06
COX6A2	0,06
HIST1H4L	0,06
MIR302D	0,05
PRLH	0,05
OR4S2	0,05
LINC00895	0,05
OR51B6	0,04
OXT	0,04
OR52W1	0,04
OR4C6	0,03
SNORA39	0,03
RPS26	0,02
MIR4633	0,00
MIR599	0,00
MIR323A	0,00
MIR330	0,00
MIR1200	0,00
MIR4540	0,00
MIR4642	0,00
MIR3909	0,00
MIR5047	0,00
MIR624	0,00
SNORD56B	0,00
MIRLET7E	0,00
MIR4758	0,00
MIR3679	0,00
SCARNA8	0,00
MIR181D	0,00
MIR337	0,00
MIR5700	0,00
MIR563	0,00
MIR3186	0,00
SNORD88A	0,00
MIR20B	0,00
MIR548G	0,00
SNORD115-20	0,00
SNORD112	0,00
MIR4526	0,00

RNU86	0,00
MIR4640	0,00
MIR4799	0,00
MIR3714	0,00
SNORD53	0,00
SNORD62B	0,00
MIR30B	0,00
MIR4736	0,00
MIR5699	0,00
MIR1197	0,00
SNORA3	0,00
MIR4682	0,00
MIR632	0,00
MIR152	0,00
SNORD107	0,00
SNORD116-5	0,00
MIR382	0,00
SNORD37	0,00
MIR3198-1	0,00
MIR4761	0,00
MIR4429	0,00
MIR4251	0,00
MIR101-2	0,00
MIR181A2	0,00
MIR4673	0,00
MIR875	0,00
MIR199A2	0,00
MIR4731	0,00
MIR4718	0,00
SCARNA27	0,00
MIR3148	0,00
MIR3924	0,00
MIR335	0,00
MIR4644	0,00
SNORA36A	0,00
MIR890	0,00
MIR4704	0,00
SNORA70G	0,00
MIR4486	0,00
SNORD18B	0,00
MIR127	0,00
SNORD114-19	0,00
MIR376A2	0,00
MIR487A	0,00
MIR134	0,00
MIR323B	0,00

MIR4530	0,00
MIR4751	0,00
MIR1323	0,00
MIR5011	0,00
MIR3196	0,00
MIR5690	0,00
MIR449C	0,00
MIR3938	0,00
MIR548F2	0,00
MIR548AR	0,00
MIR551A	0,00
MIR3122	0,00
MIR455	0,00
MIR147A	0,00
MIR490	0,00
MIR621	0,00
MIR4688	0,00
MIR4691	0,00
MIR3167	0,00
MIR4518	0,00
SNORD116-11	0,00
SNORD116-25	0,00
SNORD115-32	0,00
MIR4716	0,00
MIR1238	0,00
SNORD123	0,00
MIR3142	0,00
MIR135A1	0,00
MIR5000	0,00
MIR219-2	0,00
MIR2964A	0,00
KRTAP6-2	0,00
MIR644B	0,00
MIR3128	0,00
SNORD77	0,00
SNORD54	0,00
MIR1203	0,00
SNORD116-28	0,00
MIR549	0,00
MIR130B	0,00
MIR3199-1	0,00
MIR3199-2	0,00
MIR145	0,00
SNORD11B	0,00
MIR3119-2	0,00
MIR5194	0,00

SCARNA11	0,00
HSPB9	0,00
CYCSP52	0,00
UBE2DNL	0,00
OR10AG1	0,00
OR10S1	0,00
MIR362	0,00
MIR1468	0,00
MIR223	0,00
MIR374A	0,00
MIR19B2	0,00
MIR4299	0,00
MIR129-2	0,00
MIR4493	0,00
MIR1265	0,00
MIR1253	0,00
MIR4723	0,00
MIR451A	0,00
MIR451B	0,00
MIR548AA2	0,00
SCARNA16	0,00
MIR1538	0,00
SNORD115-10	0,00
SNORD115-19	0,00
MIR211	0,00
MIR2116	0,00
MIR433	0,00
SNORD113-4	0,00
SNORD114-13	0,00
MIR3188	0,00
MIR4324	0,00
MIR519D	0,00
MIR526A2	0,00
MIR1283-2	0,00
MIR373	0,00
MIRLET7B	0,00
MIR1292	0,00
MIR646	0,00
MIR155	0,00
KRTAP19-7	0,00
MIR3146	0,00
MIR4657	0,00
MIR4284	0,00
MIR3666	0,00
MIR29A	0,00
MIR548AR	0,00

MIR4647	0,00
MIR3668	0,00
MIR4456	0,00
MIR4457	0,00
MIR4636	0,00
MIR5197	0,00
MIR3912	0,00
MIR4800	0,00
MIR302B	0,00
MIR1243	0,00
MIR4788	0,00
SNORD2	0,00
MIR3127	0,00
MIR4771-2	0,00
MIR4268	0,00
MIR4632	0,00
MIR3675	0,00
MIR3115	0,00
SNORD21	0,00
MIR760	0,00
MIR4654	0,00
SNORD76	0,00
MIR4476	0,00
MIR486	0,00
MIR1205	0,00
MIR937	0,00
KRTAP19-3	0,00
TYRO3P	0,00
KRTAP4-1	0,00
DPPA2P3	0,00
PPP1R2P9	0,00
OR8J3	0,00
SNORD15A	0,00
SNORA10	0,00
SNORA75	0,00
SNORA16A	0,00
CLDN25	0,00
MIR450A1	0,00
MIR17	0,00
OR1D5	0,00
SNORD116-1	0,00
SNORD115-3	0,00
MIR1282	0,00
MIR3936	0,00
MIR5702	0,00
MIR5191	0,00

MIR1231	0,00
LINC01003	0,00
OR52E6	0,00
SNORA53	0,00
OR5E1P	0,00
RNASE1	0,00
MIR4686	0,00
MIR4480	0,00
MIR4521	0,00
SNORA38B	0,00
MIR3529	0,00
MIR208B	0,00
SNORA79	0,00
MIR1275	0,00
SNORA44	0,00
SNORD78	0,00
SNORD44	0,00
SNORD75	0,00
MIR4670	0,00
OR4D5	0,00
HIST1H4E	0,00
MIR651	0,00
MIR4305	0,00
MIR15A	0,00
MIR331	0,00
MIR548K	0,00
SNORD91B	0,00
SNORD116-2	0,00
SNORD115-2	0,00
MIR518E	0,00
SNORD58C	0,00
MIR122	0,00
MIR578	0,00
MIR5192	0,00
MIR200A	0,00
MIR554	0,00
MIR204	0,00
GSTM2P1	0,00
LYPD8	0,00
SNORA48	0,00
SNORA24	0,00
SCARNA9	0,00
OR52J3	0,00
RNASE1	0,00
TAS2R7	0,00
OR8G2	0,00

HIST1H4B	0,00
KRTAP12-3	0,00
OR5AU1	0,00
SPRR4	0,00
CGB1	0,00
OR5K1	0,00
APOOP5	0,00
MIR1911	0,00
MIR542	0,00
MIR4499	0,00
SNORD102	0,00
MIR18A	0,00
MIR613	0,00
MIR615	0,00
MIR1252	0,00
MIR4700	0,00
MIR4491	0,00
MIR125B1	0,00
MIR5692C2	0,00
MIR3184	0,00
MIR2909	0,00
MIR196A1	0,00
SNORA76	0,00
MIR3064	0,00
MIR4721	0,00
MIR4508	0,00
SNORD116-12	0,00
SNORD116-17	0,00
MIR628	0,00
SNORD127	0,00
MIR4709	0,00
MIR665	0,00
SNORD113-8	0,00
SNORD114-11	0,00
MIR543	0,00
MIR668	0,00
MIR656	0,00
MIR4322	0,00
SNORD41	0,00
MIR181C	0,00
MIR639	0,00
MIR3189	0,00
MIR4531	0,00
SNAR-C1	0,00
SNAR-C3	0,00
MIR518A1	0,00

MIR371A	0,00
MIR371B	0,00
MIR4320	0,00
MIR3591	0,00
MIR650	0,00
SNORA71C	0,00
MIR3194	0,00
MIR941-1	0,00
KRTAP27-1	0,00
SNORA80	0,00
MIR4658	0,00
MIR3691	0,00
SNORD48	0,00
MIR219-1	0,00
MIR30A	0,00
MIR3939	0,00
MIR4280	0,00
VTRNA1-3	0,00
MIR103A1	0,00
MIR103B1	0,00
MIR943	0,00
MIR4798	0,00
MIR4802	0,00
MIR1973	0,00
SNORD73A	0,00
MIR3945	0,00
MIR191	0,00
MIR4787	0,00
MIR3136	0,00
OR5H14	0,00
MIR568	0,00
MIR5704	0,00
PISRT1	0,00
MIR3919	0,00
MIR944	0,00
MIR570	0,00
MIR4797	0,00
MIR4261	0,00
MIR4778	0,00
MIR4264	0,00
MIR378F	0,00
MIR4420	0,00
MIR5584	0,00
MIR3671	0,00
MIR5087	0,00
MIR4424	0,00

MIR4735	0,00
MIR664	0,00
MIR4742	0,00
MIR5008	0,00
MIR3620	0,00
MIR3153	0,00
MIR2278	0,00
MIR3689D2	0,00
MIR5708	0,00
MIR3150A	0,00
MIR1206	0,00
MIR4472-1	0,00
OR51G2	0,00
ACTL7A	0,00
OR52A1	0,00
OR6Q1	0,00
OR4K13	0,00
OR2A2	0,00
KRTAP13-2	0,00
OR8J1	0,00
HIST1H2BJ	0,00
IQCF4	0,00
KRTAP5-3	0,00
OR2Z1	0,00
KRTAP9-8	0,00
PCNA-AS1	0,00
MTRNR2L10	0,00
OR4F5	0,00
TAL2	0,00
OR6S1	0,00
SNORA45	0,00
SNAR-F	0,00
SNORA51	0,00
SNORA42	0,00
SCARNA12	0,00
KRTAP4-2	0,00
RPS28	0,00
KRTAP10-4	0,00
MIR532	0,00
MIR452	0,00
MIR105-2	0,00
MIR623	0,00
MIR1244-1	0,00
MIR3973	0,00
MIR3160-1	0,00
MIR3160-2	0,00

MIR3162	0,00
MIR423	0,00
MIR3615	0,00
SNORD116-22	0,00
SNORD115-41	0,00
MIR4515	0,00
SNORD8	0,00
MIR758	0,00
MIR654	0,00
MIR7-3	0,00
SNAR-D	0,00
MIR523	0,00
MIR124-3	0,00
MIR3943	0,00
MIR590	0,00
MIR182	0,00
MIR671	0,00
MIR586	0,00
MIR2113	0,00
MIR4277	0,00
MIR5688	0,00
MIR4796	0,00
TERC	0,00
MIR4432	0,00
MIR30E	0,00
SNORD45A	0,00
MIR488	0,00
MIRLET7D	0,00
MIR124-1	0,00
C11orf96	0,00
OR6C4	0,00
PYDC2	0,00
HIST3H2A	0,00
DPPA5	0,00
CHKB-AS1	0,00
PIN1P1	0,00
IFNA4	0,00
CGB5	0,00
CLDN24	0,00
OR4P4	0,00
HIST1H3B	0,00
KRTAP10-3	0,00
MTRNR2L9	0,00
CDRT15P2	0,00
KRTAP10-12	0,00
OR4L1	0,00

HIST1H2AE	0,00
ZNF847P	0,00
MIR363	0,00
MIR503	0,00
SNORA57	0,00
SNORD31	0,00
UBTFL1	0,00
MIR4693	0,00
OR10G4	0,00
SNORD42B	0,00
SNORD1C	0,00
SNORD115-24	0,00
SCARNA15	0,00
SNORD113-2	0,00
SNORD113-3	0,00
SNORD114-18	0,00
MIR329-1	0,00
MIR1193	0,00
OR7G3	0,00
MIR4750	0,00
SNORD58B	0,00
MIR103A2	0,00
MIR103B2	0,00
SNORA71B	0,00
MIR1914	0,00
MIR4650-1	0,00
MIR3139	0,00
SNORD20	0,00
SNORA36B	0,00
OR1L8	0,00
SNORD36A	0,00
DEFA8P	0,00
MIR4287	0,00
KRTAP5-4	0,00
CDRT15L2	0,00
OR5B3	0,00
HBQ1	0,00
FAM223B	0,00
VPS13A-AS1	0,00
ECRP	0,00
KRTAP21-2	0,00
OR8B8	0,00
HIST2H3D	0,00
FAM127B	0,00
MPC1L	0,00
HIST1H4C	0,00

VCX3B	0,00
IFNA13	0,00
LILRB2	0,00
C10orf120	0,00
OR4D10	0,00
MIR98	0,00
TGIF2LX	0,00
MIR448	0,00
MIR2276	0,00
MIR26A2	0,00
MIR210	0,00
MIR34B	0,00
OR8B3	0,00
MIR608	0,00
MIR138-2	0,00
SNORD116-9	0,00
SNORD115-11	0,00
SNORD115-15	0,00
SNORD115-38	0,00
MIR3942	0,00
MIR627	0,00
MIR125A	0,00
MIR521-1	0,00
MIR4764	0,00
MIR941-3	0,00
MIR941-2	0,00
MIR941-4	0,00
HIST1H3E	0,00
TREML5P	0,00
MIR567	0,00
MIR1244-1	0,00
MIR5001	0,00
MIR200B	0,00
MIR3917	0,00
MIR92B	0,00
MIR4753	0,00
OR2L5	0,00
MIR491	0,00
MIR4674	0,00
OR11H12	0,00
RNASE2	0,00
NPBWR1	0,00
APOA4	0,00
CTAGE9	0,00
MSMP	0,00
KRT19P2	0,00

HIST1H1C	0,00
HBG1	0,00
MIR3654	0,00
ATP5L2	0,00
PF4	0,00
KRT16P1	0,00
RNASE10	0,00
SPANXD	0,00
KRTAP10-1	0,00
HIST2H2BF	0,00
HIST1H4K	0,00
SPANXE	0,00
CSH2	0,00
OR4C46	0,00
OR4E2	0,00
OR3A3	0,00
HBM	0,00
UBE2MP1	0,00
OR11H2	0,00
LOC392364	0,00
RPL10L	0,00
RHOXF2	0,00
RHOXF2B	0,00
OR2M2	0,00
CYP21A1P	0,00
HIST1H2APS1	0,00
PAGE2	0,00
FAM106A	0,00
TAS2R42	0,00
OR6C68	0,00
OR7C2	0,00
OR2A7	0,00
HIST3H2BB	0,00
SPAG11B	0,00
KRTAP10-11	0,00
CCRL2	0,00
LOC643648	0,00
FAM90A27P	0,00
TMEM191B	0,00
CCRL2	0,00
GOLGA6A	0,00
SPDYE2B	0,00
CLEC11A	0,00
HERC2P4	0,00
PRAMEF21	0,00
LOC100133920	0,00

FAM27E3	0,00
ARHGDIA	0,00
VCX3A	0,00
FOXD4L5	0,00
SPACA5B	0,00
HIST1H4F	0,00
USP17L1P	0,00
HSPA1A	0,00
CTAG2	0,00
LOC100134868	0,00
PNMA6A	0,00
APOA1	0,00
CCR2	0,00
HIST1H2BM	0,00
OR2T29	0,00
DEFB4B	0,00
SPDYE8P	0,00
LGALS7	0,00
PNMA6D	0,00
CTAGE15	0,00
TRIM64	0,00
PRAMEF20	0,00
CCR2	0,00
GAGE2B	0,00
FLJ44511	0,00
SPATA31A2	0,00
SPATA31A1	0,00
HERC2P7	0,00
DEFB106B	0,00
GOLGA8CP	0,00
CCR5	0,00
LOC100506076	0,00
HSFX2	0,00
HSFX1	0,00
SLX1B-SULT1A4	0,00
MLLT4-AS1	0,00
GAGE12J	0,00
SLX1B	0,00
DMRTC1B	0,00
TAS1R3	0,00
LOC643648	0,00
TBC1D3F	0,00
TBC1D3C	0,00
TBC1D3	0,00
DNM1P41	0,00
GOLGA6C	0,00

STAG3L1	0,00
TBC1D3B	0,00
USP17L6P	0,00
LOC100190986	0,00
SSX4B	0,00
SSX4	0,00
SULT1A4	0,00
PRAMEF8	0,00
FAM95B1	0,00
SRSF10	0,00
GAGE12I	0,00
GAGE7	0,00
GAGE4	0,00
GAGE5	0,00
BEND3P3	0,00
LINC00891	0,00
ACOT1	0,00
SPDYE8P	0,00
SRSF10	0,00
ARL17B	0,00
LINC00854	0,00
GYG2P1	0,00
LOC100505679	0,00
PCYT2	0,00
PCYT2	0,00
NXF2B	0,00
PCYT2	0,00
FAM74A1	0,00
C6orf123	0,00
GOLGA8G	0,00
DTYMK	0,00
TTTY15	0,00
DGAT1	0,00
KIR2DS3	0,00
PDZK1P1	0,00
KIR2DS5	0,00
LIMS3-LOC440895	0,00
EIF1AY	0,00
P4HB	0,00
CT45A4	0,00
FAM66E	0,00
TMPRSS11E	0,00
PCDH11Y	0,00
TBL1Y	0,00

Table **II** Gene symbol, mice normalized intensity of reads, mean of the duplicate, and hmC Fold enrichment (Dko vs wt), related to Fig. 3.a

Gene symbol	Mouse 16756 (D*ko*#1) normalized intensity of reads	Mouse 16641 (D*ko*#2) normalized intensity of reads	Mouse 16670 (WT#1) normalized intensity of reads	Mouse 16669 (WT#2) normalized intensity of reads	*GPx*1/2 D*ko* intensity MEAN	WT intensity MEAN	hmC Fold enrichment (D*ko* vs wt)
Mir3076	0,00	0,00	0,34	0,14	0,00	0,24	0,00
Snord58b	0,00	0,00	0,17	0,25	0,00	0,21	0,00
Mir92-1	0,00	0,00	0,34	0,07	0,00	0,20	0,00
Mir26b	0,00	0,00	0,21	0,19	0,00	0,20	0,00
Mir434	0,00	0,00	0,26	0,11	0,00	0,18	0,00
Mir10a	0,00	0,00	0,26	0,11	0,00	0,18	0,00
Mir488	0,00	0,00	0,34	0,01	0,00	0,17	0,00
Mir3103	0,00	0,00	0,34	0,00	0,00	0,17	0,00
Snord100	0,00	0,00	0,06	0,27	0,00	0,16	0,00
Mir322	0,00	0,00	0,22	0,04	0,00	0,13	0,00
Mir139	0,00	0,00	0,22	0,04	0,00	0,13	0,00
Mir191	0,00	0,00	0,06	0,18	0,00	0,12	0,00
Sprr4	0,00	0,00	0,08	0,14	0,00	0,11	0,00
Mir106a	0,00	0,00	0,22	0,00	0,00	0,11	0,00
Mir540	0,00	0,00	0,22	0,00	0,00	0,11	0,00
Mir1247	0,00	0,00	0,22	0,00	0,00	0,11	0,00
Mir193b	0,00	0,00	0,22	0,00	0,00	0,11	0,00
Mir706	0,00	0,00	0,00	0,21	0,00	0,11	0,00
Mirlet7g	0,00	0,00	0,17	0,04	0,00	0,10	0,00
Mir7-2	0,00	0,02	0,17	0,08	0,01	0,13	0,07
Mir3061	0,00	0,02	0,04	0,21	0,01	0,13	0,07
Mir1192	0,00	0,04	0,28	0,20	0,02	0,24	0,08
Krtap19-3	0,00	0,03	0,24	0,08	0,02	0,16	0,10
Snora34	0,00	0,05	0,47	0,04	0,03	0,25	0,10
Mir3075	0,00	0,03	0,17	0,17	0,02	0,17	0,10
Mir31	0,00	0,03	0,11	0,21	0,02	0,16	0,11

Scarna13	0,00	0,04	0,37	0,05	0,02	0,21	0,11
Snord123	0,00	0,07	0,45	0,17	0,03	0,31	0,11
Mir103-1	0,00	0,07	0,22	0,39	0,03	0,31	0,11
Snora2b	0,00	0,03	0,15	0,15	0,02	0,15	0,11
Mir1930	0,00	0,05	0,22	0,21	0,03	0,22	0,12
Mir15a	0,00	0,03	0,09	0,19	0,02	0,14	0,12
Mir1961	0,00	0,10	0,22	0,58	0,05	0,40	0,13
Mir30a	0,00	0,10	0,48	0,30	0,05	0,39	0,13
Snord35b	0,00	0,12	0,58	0,30	0,06	0,44	0,14
Snora75	0,00	0,03	0,22	0,01	0,02	0,12	0,15
Mir468	0,00	0,05	0,28	0,05	0,03	0,17	0,15
Mir326	0,00	0,14	0,39	0,48	0,07	0,44	0,16
Gm5547	0,00	0,05	0,16	0,15	0,02	0,16	0,16
Hps6	0,00	0,05	0,14	0,16	0,02	0,15	0,16
Snord69	0,00	0,05	0,11	0,20	0,03	0,15	0,17
A630066F11Rik	0,02	0,03	0,18	0,08	0,02	0,13	0,17
Mir511	0,00	0,05	0,22	0,07	0,03	0,15	0,17
Mir3095	0,00	0,14	0,43	0,34	0,07	0,39	0,18
Snord96a	0,00	0,05	0,06	0,23	0,03	0,14	0,18
Mir1929	0,00	0,07	0,19	0,19	0,03	0,19	0,18
Snord1b	0,00	0,13	0,51	0,18	0,06	0,34	0,19
Isg15	0,10	0,04	0,44	0,27	0,07	0,36	0,19
D4Wsu53e	0,00	0,06	0,18	0,12	0,03	0,15	0,20
Gm13580	0,00	0,07	0,18	0,16	0,03	0,17	0,20
Mir429	0,00	0,22	0,32	0,77	0,11	0,54	0,21
Sstr2	0,01	0,09	0,20	0,28	0,05	0,24	0,21
1600014C23Rik	0,00	0,06	0,19	0,09	0,03	0,14	0,21
Snora28	0,00	0,17	0,42	0,38	0,08	0,40	0,21
Mia	0,04	0,09	0,35	0,26	0,06	0,31	0,21
Mir763	0,00	0,19	0,49	0,40	0,09	0,44	0,21
Crip1	0,06	0,13	0,47	0,38	0,09	0,43	0,21
Mir26a-1	0,00	0,09	0,32	0,08	0,04	0,20	0,21

H2-Q8	0,05	0,03	0,21	0,12	0,04	0,17	0,21
Mir147	0,00	0,10	0,34	0,14	0,05	0,24	0,22
Snord37	0,27	0,21	1,32	0,89	0,24	1,10	0,22
H2-Q6	0,05	0,03	0,21	0,12	0,04	0,17	0,22
Cyhr1	0,05	0,09	0,39	0,23	0,07	0,31	0,23
Leng9	0,02	0,04	0,18	0,09	0,03	0,13	0,23
Aprt	0,05	0,04	0,23	0,14	0,04	0,19	0,23
Sstr2	0,01	0,16	0,29	0,42	0,08	0,36	0,24
Nfe2l1	0,02	0,06	0,19	0,15	0,04	0,17	0,24
Nfe2l1	0,02	0,06	0,19	0,16	0,04	0,17	0,24
Mir20a	0,00	0,21	0,34	0,53	0,10	0,43	0,24
Pck1	0,13	0,15	0,65	0,50	0,14	0,58	0,24
Cct8l1	0,01	0,08	0,17	0,24	0,05	0,20	0,24
Gjb5	0,02	0,11	0,25	0,25	0,06	0,25	0,24
Cdx2	0,05	0,04	0,21	0,14	0,04	0,17	0,24
Mdp1	0,02	0,15	0,37	0,34	0,09	0,35	0,25
Nat9	0,07	0,07	0,31	0,26	0,07	0,28	0,25
Hrc	0,01	0,07	0,14	0,20	0,04	0,17	0,25
Pabpn1	0,01	0,07	0,19	0,15	0,04	0,17	0,25
Gm5111	0,05	0,04	0,07	0,28	0,04	0,17	0,26
Mir1982	0,00	0,16	0,42	0,18	0,08	0,30	0,26
Lenep	0,04	0,05	0,29	0,08	0,05	0,19	0,26
Aurkaip1	0,03	0,04	0,14	0,14	0,04	0,14	0,26
Cyhr1	0,04	0,09	0,30	0,18	0,07	0,24	0,27
Mir3069	0,00	0,16	0,45	0,12	0,08	0,29	0,27
Tigd5	0,03	0,07	0,20	0,20	0,05	0,20	0,27
Mirlet7c-2	0,00	0,40	0,62	0,82	0,20	0,72	0,28
Gpbar1	0,00	0,08	0,16	0,12	0,04	0,14	0,28
Mir1981	0,27	0,24	0,86	0,97	0,26	0,92	0,28
Mir1190	0,00	0,22	0,39	0,39	0,11	0,39	0,28
Dbil5	0,10	0,07	0,39	0,22	0,09	0,30	0,28
Gm14873	0,00	0,12	0,16	0,26	0,06	0,21	0,28

1700018L02Rik	0,00	0,15	0,32	0,23	0,08	0,27	0,28
Coa4	0,04	0,12	0,31	0,24	0,08	0,27	0,29
Mir29c	0,18	0,05	0,47	0,34	0,12	0,41	0,29
Mir340	0,27	0,19	0,94	0,66	0,23	0,80	0,29
BC048609	0,09	0,06	0,33	0,18	0,07	0,26	0,29
4930539J05Rik	0,07	0,05	0,20	0,20	0,06	0,20	0,29
S100a16	0,06	0,09	0,30	0,18	0,07	0,24	0,30
AA388235	0,06	0,05	0,22	0,15	0,06	0,19	0,30
Cyp8b1	0,11	0,12	0,38	0,34	0,11	0,36	0,31
Defb45	0,07	0,06	0,19	0,23	0,06	0,21	0,31
Cend1	0,07	0,10	0,33	0,23	0,09	0,28	0,31
Gstp2	0,07	0,08	0,28	0,22	0,08	0,25	0,31
Mir505	0,00	0,16	0,28	0,22	0,08	0,25	0,31
BC068157	0,03	0,07	0,16	0,16	0,05	0,16	0,31
Ankrd9	0,13	0,18	0,56	0,44	0,16	0,50	0,31
Mir1839	0,00	0,23	0,34	0,41	0,12	0,37	0,31
Mir3058	0,27	0,48	1,31	1,10	0,38	1,20	0,31
Tgm3	0,07	0,05	0,21	0,20	0,06	0,20	0,31
Gdap10	0,07	0,10	0,25	0,27	0,08	0,26	0,31
Atp12a	0,05	0,05	0,18	0,14	0,05	0,16	0,32
Mpv17l2	0,15	0,08	0,43	0,32	0,12	0,37	0,32
Tnp2	0,00	0,12	0,19	0,19	0,06	0,19	0,32
Mir3063	0,00	0,12	0,26	0,12	0,06	0,19	0,32
Fhl1	0,04	0,05	0,14	0,16	0,05	0,15	0,32
Snord16a	0,00	0,10	0,11	0,21	0,05	0,16	0,32
Tusc2	0,11	0,06	0,26	0,28	0,09	0,27	0,32
Dcaf11	0,06	0,04	0,20	0,11	0,05	0,15	0,32
Gm10485	0,04	0,12	0,26	0,24	0,08	0,25	0,33
Slc16a11	0,05	0,10	0,27	0,20	0,08	0,23	0,33
Tppp	0,09	0,07	0,25	0,22	0,08	0,24	0,33
Mir3968	0,59	0,38	1,78	1,16	0,48	1,47	0,33
Mir532	0,00	0,16	0,19	0,28	0,08	0,24	0,33

1700119H24Rik	0,12	0,09	0,36	0,29	0,11	0,32	0,33
Mir205	0,14	0,00	0,00	0,41	0,07	0,20	0,33
Tbx6	0,08	0,08	0,29	0,20	0,08	0,25	0,33
Cldn9	0,02	0,10	0,16	0,19	0,06	0,18	0,33
Gm757	0,04	0,06	0,17	0,14	0,05	0,16	0,33
Fv1	0,06	0,05	0,17	0,15	0,05	0,16	0,34
Sertad1	0,15	0,08	0,41	0,26	0,11	0,34	0,34
4933427E11Rik	0,09	0,07	0,28	0,20	0,08	0,24	0,34
Stub1	0,04	0,12	0,26	0,21	0,08	0,24	0,34
Aqp8	0,06	0,08	0,22	0,20	0,07	0,21	0,34
Rxfp4	0,18	0,15	0,54	0,42	0,16	0,48	0,34
Gpr137	0,07	0,12	0,35	0,22	0,10	0,29	0,34
Gpr137	0,08	0,13	0,38	0,23	0,11	0,31	0,34
Gstm5	0,06	0,05	0,14	0,17	0,05	0,16	0,34
Vamp8	0,05	0,07	0,23	0,14	0,06	0,18	0,34
Slc50a1	0,02	0,24	0,40	0,34	0,13	0,37	0,35
Gpr137	0,06	0,10	0,28	0,17	0,08	0,23	0,35
Vmo1	0,12	0,03	0,21	0,19	0,07	0,20	0,35
Map1lc3a	0,02	0,11	0,18	0,18	0,06	0,18	0,36
Efna1	0,10	0,09	0,29	0,24	0,10	0,26	0,36
Dusp28	0,18	0,07	0,40	0,28	0,12	0,34	0,36
Pnpo	0,15	0,11	0,41	0,31	0,13	0,36	0,36
Ndufv1	0,05	0,07	0,21	0,12	0,06	0,16	0,36
Cyp2c55	0,08	0,04	0,15	0,17	0,06	0,16	0,36
Efna1	0,12	0,10	0,32	0,28	0,11	0,30	0,36
5033406O09Rik	0,07	0,13	0,31	0,22	0,10	0,27	0,37
Pmp22	0,10	0,09	0,27	0,25	0,10	0,26	0,37
Gm2061	0,18	0,09	0,38	0,34	0,13	0,36	0,37
Khnyn	0,10	0,09	0,29	0,24	0,10	0,27	0,37
Psmb8	0,09	0,09	0,25	0,23	0,09	0,24	0,37
Polr1c	0,03	0,12	0,23	0,17	0,07	0,20	0,37
Mir26a-2	0,00	0,19	0,36	0,15	0,09	0,25	0,37

Espn	0,13	0,09	0,32	0,28	0,11	0,30	0,37
Lefty1	0,08	0,13	0,31	0,26	0,11	0,28	0,37
Sigmar1	0,08	0,08	0,22	0,21	0,08	0,21	0,37
Spaca4	0,12	0,33	0,78	0,41	0,23	0,60	0,38
Mir700	0,00	0,23	0,42	0,20	0,12	0,31	0,38
Mapk15	0,08	0,10	0,28	0,18	0,09	0,23	0,38
Ppp1r27	0,10	0,03	0,17	0,18	0,07	0,18	0,38
Pfdn5	0,03	0,11	0,22	0,14	0,07	0,18	0,38
Hcfc1r1	0,00	0,14	0,20	0,17	0,07	0,19	0,38
Arhgdia	0,09	0,10	0,29	0,21	0,10	0,25	0,38
Mir33	0,00	0,13	0,34	0,00	0,06	0,17	0,38
Tmem115	0,08	0,08	0,26	0,18	0,08	0,22	0,38
Gm6654	0,15	0,09	0,42	0,20	0,12	0,31	0,39
Rnf223	0,10	0,07	0,18	0,26	0,08	0,22	0,39
Fau	0,10	0,04	0,22	0,15	0,07	0,19	0,39
Slc20a1	0,13	0,09	0,28	0,29	0,11	0,29	0,39
Gpt	0,14	0,16	0,45	0,31	0,15	0,38	0,39
Mir704	0,00	0,13	0,22	0,11	0,06	0,17	0,39
Fau	0,10	0,05	0,23	0,16	0,08	0,19	0,39
Ift20	0,06	0,07	0,21	0,13	0,07	0,17	0,39
Tmed6	0,06	0,08	0,19	0,18	0,07	0,19	0,39
Rfxank	0,06	0,08	0,19	0,16	0,07	0,17	0,39
AI854703	0,11	0,11	0,30	0,27	0,11	0,28	0,39
Slc26a3	0,14	0,13	0,34	0,33	0,13	0,34	0,39
Fam134b	0,13	0,09	0,28	0,26	0,11	0,27	0,39
Fbxo32	0,08	0,08	0,22	0,19	0,08	0,20	0,40
A430035B10Rik	0,21	0,10	0,44	0,35	0,16	0,39	0,40
Fam134b	0,13	0,09	0,29	0,28	0,11	0,29	0,40
Ihh	0,06	0,09	0,18	0,20	0,08	0,19	0,40
Coasy	0,06	0,11	0,26	0,17	0,09	0,21	0,40
Klhdc3	0,05	0,12	0,23	0,21	0,09	0,22	0,40
Fam134b	0,13	0,09	0,29	0,28	0,11	0,28	0,40

Slc20a1	0,10	0,09	0,26	0,23	0,10	0,25	0,40
Atp5b	0,16	0,10	0,39	0,25	0,13	0,32	0,40
D830046C22Rik	0,10	0,11	0,30	0,21	0,10	0,26	0,40
Cox6a1	0,09	0,09	0,23	0,21	0,09	0,22	0,40
Pmaip1	0,11	0,13	0,31	0,29	0,12	0,30	0,40
Trim15	0,08	0,08	0,23	0,19	0,08	0,21	0,40
Trim15	0,08	0,08	0,23	0,19	0,08	0,21	0,40
Slc39a5	0,04	0,10	0,16	0,18	0,07	0,17	0,40
Slc39a5	0,04	0,12	0,19	0,21	0,08	0,20	0,40
Slc39a5	0,04	0,12	0,19	0,20	0,08	0,19	0,40
Commd5	0,08	0,08	0,22	0,18	0,08	0,20	0,41
Ankrd23	0,10	0,08	0,25	0,20	0,09	0,22	0,41
Fam134b	0,14	0,09	0,30	0,27	0,12	0,29	0,41
Tmem174	0,09	0,20	0,40	0,33	0,15	0,36	0,41
Tnfrsf25	0,11	0,06	0,21	0,21	0,09	0,21	0,41
Cox14	0,12	0,12	0,33	0,26	0,12	0,29	0,41
AI837181	0,18	0,13	0,45	0,30	0,15	0,38	0,41
Tmem79	0,06	0,10	0,23	0,17	0,08	0,20	0,41
Adh1	0,12	0,07	0,23	0,23	0,10	0,23	0,41
Eif4ebp3	0,14	0,07	0,27	0,24	0,11	0,26	0,41
Fam166a	0,05	0,10	0,25	0,13	0,08	0,19	0,41
Pdp2	0,25	0,17	0,50	0,52	0,21	0,51	0,41
Tctex1d4	0,20	0,17	0,42	0,49	0,19	0,45	0,41
Snord71	0,00	0,18	0,22	0,21	0,09	0,22	0,41
Nfe2l1	0,07	0,09	0,23	0,16	0,08	0,19	0,41
Lsm4	0,18	0,11	0,43	0,27	0,15	0,35	0,41
AU023871	0,15	0,10	0,30	0,30	0,12	0,30	0,42
Cdk3-ps	0,05	0,16	0,29	0,22	0,11	0,25	0,42
Rogdi	0,13	0,15	0,37	0,31	0,14	0,34	0,42
Nfe2l1	0,07	0,09	0,22	0,16	0,08	0,19	0,42
Uqcr10	0,07	0,08	0,24	0,11	0,07	0,17	0,42
Cdk2ap2	0,14	0,24	0,47	0,41	0,19	0,44	0,42

G0s2	0,05	0,12	0,11	0,29	0,08	0,20	0,42
Zfp707	0,13	0,09	0,25	0,26	0,11	0,26	0,42
Aldoa	0,14	0,06	0,30	0,18	0,10	0,24	0,42
Aldoa	0,14	0,06	0,31	0,18	0,10	0,24	0,42
1810007I06Rik	0,07	0,08	0,20	0,17	0,08	0,18	0,42
Lsm10	0,08	0,10	0,23	0,20	0,09	0,21	0,43
Smdt1	0,21	0,16	0,50	0,38	0,19	0,44	0,43
Gpsm3	0,11	0,11	0,34	0,17	0,11	0,25	0,43
Slc10a5	0,13	0,22	0,40	0,43	0,18	0,41	0,43
Ly6g6c	0,12	0,10	0,27	0,24	0,11	0,26	0,43
Fam83e	0,17	0,21	0,53	0,35	0,19	0,44	0,43
Snx15	0,14	0,14	0,35	0,30	0,14	0,33	0,43
Dnase2a	0,07	0,10	0,22	0,16	0,08	0,19	0,43
Atp6v0b	0,04	0,11	0,18	0,18	0,08	0,18	0,43
Ckmt1	0,18	0,15	0,37	0,40	0,17	0,38	0,43
Brk1	0,10	0,11	0,27	0,21	0,10	0,24	0,43
Mirlet7d	0,09	0,29	0,45	0,44	0,19	0,44	0,43
Sult1a1	0,17	0,14	0,37	0,33	0,15	0,35	0,43
Ptger1	0,15	0,17	0,40	0,33	0,16	0,37	0,43
Slc4a2	0,11	0,09	0,24	0,22	0,10	0,23	0,43
Mxd1	0,27	0,20	0,56	0,51	0,23	0,53	0,43
Pnkd	0,12	0,07	0,28	0,17	0,10	0,22	0,44
Gjb3	0,10	0,06	0,21	0,17	0,08	0,19	0,44
BC037034	0,09	0,10	0,20	0,24	0,10	0,22	0,44
Rps4y2	0,17	0,07	0,37	0,18	0,12	0,28	0,44
2010010A06Rik	0,14	0,14	0,34	0,30	0,14	0,32	0,44
Usp2	0,10	0,09	0,20	0,23	0,09	0,22	0,44
Gjb3	0,15	0,09	0,29	0,23	0,12	0,26	0,44
Tmem258	0,05	0,16	0,24	0,22	0,10	0,23	0,44
Tsc22d1	0,16	0,20	0,34	0,47	0,18	0,41	0,44
Aldoa	0,13	0,05	0,26	0,15	0,09	0,20	0,44
Slc15a1	0,09	0,07	0,18	0,20	0,08	0,19	0,44

Dusp1	0,13	0,10	0,22	0,28	0,11	0,25	0,44
4930483J18Rik	0,08	0,13	0,29	0,19	0,11	0,24	0,44
Spef1	0,05	0,11	0,21	0,16	0,08	0,18	0,44
Ccng2	0,15	0,07	0,22	0,28	0,11	0,25	0,44
Ensa	0,15	0,10	0,36	0,19	0,12	0,28	0,44
Cd68	0,09	0,24	0,47	0,26	0,16	0,37	0,44
Acaa1a	0,12	0,07	0,21	0,20	0,09	0,21	0,44
Cdkn2b	0,10	0,15	0,29	0,28	0,13	0,28	0,44
Retsat	0,10	0,15	0,30	0,27	0,13	0,28	0,44
Hist1h1t	0,14	0,42	0,65	0,62	0,28	0,64	0,44
Mir761	0,00	0,18	0,34	0,07	0,09	0,20	0,44
Gm14446	0,13	0,12	0,30	0,28	0,13	0,29	0,44
Ube2m	0,10	0,07	0,21	0,17	0,08	0,19	0,44
Ube2m	0,11	0,08	0,24	0,19	0,10	0,22	0,44
Plk3	0,13	0,19	0,34	0,37	0,16	0,35	0,44
Cideb	0,10	0,10	0,21	0,24	0,10	0,23	0,44
Krt15	0,11	0,07	0,24	0,16	0,09	0,20	0,45
Hspb9	0,07	0,11	0,21	0,20	0,09	0,21	0,45
Mir677	0,81	0,23	1,46	0,89	0,52	1,17	0,45
Gtpbp2	0,14	0,15	0,33	0,33	0,15	0,33	0,45
Mir1904	0,00	0,36	0,42	0,39	0,18	0,41	0,45
Sema4g	0,13	0,12	0,30	0,27	0,13	0,28	0,45
Sowahb	0,26	0,20	0,55	0,48	0,23	0,51	0,45
Fis1	0,04	0,16	0,23	0,21	0,10	0,22	0,45
Polr2e	0,07	0,10	0,17	0,20	0,08	0,19	0,45
Espn	0,13	0,09	0,26	0,23	0,11	0,24	0,45
Slc4a2	0,10	0,09	0,21	0,19	0,09	0,20	0,45
Slc25a35	0,16	0,12	0,34	0,27	0,14	0,30	0,45
Mat2a	0,09	0,11	0,23	0,22	0,10	0,22	0,45
2010001M06Rik	0,17	0,11	0,40	0,21	0,14	0,31	0,45
Adprm	0,08	0,14	0,25	0,23	0,11	0,24	0,45
Psma7	0,12	0,08	0,24	0,20	0,10	0,22	0,45

Entpd8	0,13	0,11	0,29	0,24	0,12	0,27	0,46
Pomgnt1	0,20	0,20	0,47	0,41	0,20	0,44	0,46
Rnf181	0,10	0,10	0,25	0,20	0,10	0,22	0,46
Trim56	0,12	0,14	0,27	0,31	0,13	0,29	0,46
Tymp	0,11	0,11	0,20	0,26	0,11	0,23	0,46
Nkiras2	0,18	0,14	0,35	0,35	0,16	0,35	0,46
Gpha2	0,02	0,20	0,32	0,17	0,11	0,25	0,46
Cldn23	0,13	0,09	0,24	0,26	0,11	0,25	0,46
Dpep1	0,10	0,10	0,24	0,20	0,10	0,22	0,46
Misp	0,16	0,20	0,37	0,43	0,18	0,40	0,46
Synpo	0,15	0,14	0,29	0,33	0,14	0,31	0,46
Prss32	0,11	0,10	0,24	0,21	0,10	0,22	0,46
Gsdmd	0,11	0,15	0,31	0,26	0,13	0,28	0,46
Gm14446	0,11	0,09	0,22	0,21	0,10	0,22	0,46
Otub1	0,12	0,10	0,23	0,25	0,11	0,24	0,46
Usp2	0,09	0,08	0,19	0,18	0,09	0,19	0,46
Fkbp1a	0,10	0,10	0,25	0,20	0,10	0,22	0,46
Igsf9	0,14	0,12	0,29	0,27	0,13	0,28	0,46
Uchl4	0,14	0,32	0,56	0,46	0,23	0,51	0,46
Igsf9	0,14	0,12	0,29	0,27	0,13	0,28	0,46
Rpl34	0,14	0,09	0,30	0,20	0,12	0,25	0,46
Chmp6	0,08	0,14	0,28	0,18	0,11	0,23	0,46
Oasl1	0,12	0,16	0,29	0,31	0,14	0,30	0,46
9630028B13Rik	0,11	0,07	0,19	0,19	0,09	0,19	0,46
Phf1	0,09	0,10	0,26	0,15	0,10	0,21	0,47
Rhbdl1	0,16	0,17	0,36	0,35	0,17	0,35	0,47
Jmjd8	0,12	0,15	0,31	0,27	0,13	0,29	0,47
Prdx6	0,20	0,18	0,42	0,39	0,19	0,41	0,47
Mrpl9	0,08	0,09	0,23	0,15	0,09	0,19	0,47
Selenbp1	0,12	0,08	0,24	0,19	0,10	0,21	0,47
Tpi1	0,21	0,15	0,45	0,33	0,18	0,39	0,47
Tmub2	0,10	0,09	0,24	0,18	0,10	0,21	0,47

Kbtbd13	0,09	0,14	0,25	0,23	0,11	0,24	0,47
Ndufa6	0,15	0,15	0,36	0,29	0,15	0,32	0,47
Tmub1	0,18	0,06	0,28	0,24	0,12	0,26	0,47
Mapk3	0,19	0,16	0,39	0,35	0,17	0,37	0,47
Eif5a	0,08	0,11	0,23	0,18	0,10	0,21	0,47
Eif5a	0,07	0,11	0,22	0,16	0,09	0,19	0,47
Map3k11	0,10	0,09	0,22	0,18	0,10	0,20	0,47
Pspn	0,20	0,01	0,18	0,26	0,11	0,22	0,47
Irf1	0,21	0,16	0,40	0,38	0,19	0,39	0,48
Inpp5j	0,15	0,11	0,26	0,27	0,13	0,27	0,48
Irf1	0,22	0,17	0,43	0,41	0,20	0,42	0,48
Zfp784	0,06	0,13	0,21	0,20	0,10	0,20	0,48
Smim15	0,18	0,15	0,42	0,28	0,17	0,35	0,48
Gmppb	0,08	0,12	0,23	0,19	0,10	0,21	0,48
Adat3	0,14	0,13	0,34	0,23	0,14	0,28	0,48
Snord17	0,18	0,09	0,45	0,11	0,13	0,28	0,48
Bad	0,14	0,09	0,26	0,21	0,12	0,24	0,48
Apoc1	0,09	0,13	0,29	0,17	0,11	0,23	0,48
Sephs2	0,31	0,09	0,47	0,37	0,20	0,42	0,48
Eid2b	0,10	0,12	0,21	0,23	0,11	0,22	0,48
Acot8	0,13	0,08	0,22	0,22	0,11	0,22	0,48
Tmem50b	0,12	0,11	0,22	0,24	0,11	0,23	0,48
Ly6g6d	0,11	0,11	0,21	0,26	0,11	0,24	0,48
1600029I14Rik	0,12	0,09	0,18	0,25	0,10	0,21	0,48
B2m	0,17	0,09	0,31	0,23	0,13	0,27	0,48
Fam134b	0,14	0,09	0,24	0,22	0,11	0,23	0,48
Eif6	0,23	0,20	0,48	0,40	0,22	0,44	0,48
Irf1	0,23	0,18	0,42	0,42	0,20	0,42	0,49
Atf5	0,10	0,11	0,24	0,19	0,10	0,21	0,49
Mir3109	0,54	0,29	1,29	0,43	0,42	0,86	0,49
Rabggtb	0,09	0,12	0,22	0,21	0,11	0,22	0,49
Tuba4a	0,14	0,15	0,32	0,28	0,15	0,30	0,49

Etfb	0,08	0,11	0,22	0,19	0,10	0,20	0,49
Zbtb12	0,33	0,22	0,63	0,49	0,28	0,56	0,49
Pcgf2	0,11	0,08	0,21	0,19	0,10	0,20	0,49
Pcgf2	0,12	0,09	0,21	0,19	0,10	0,20	0,49
Slc25a34	0,19	0,20	0,48	0,31	0,19	0,40	0,49
Dhcr7	0,11	0,08	0,20	0,20	0,10	0,20	0,49
Dhrs11	0,16	0,09	0,25	0,27	0,13	0,26	0,49
2210417A02Rik	0,12	0,08	0,19	0,22	0,10	0,20	0,49
Myl12b	0,20	0,21	0,42	0,41	0,20	0,41	0,49
Ap5s1	0,12	0,10	0,30	0,15	0,11	0,22	0,49
Ppa1	0,12	0,09	0,24	0,19	0,10	0,21	0,49
Mir3089	0,00	0,24	0,30	0,19	0,12	0,24	0,49
Apoc1	0,09	0,14	0,29	0,17	0,11	0,23	0,49
Agpat2	0,12	0,10	0,26	0,18	0,11	0,22	0,49
Slc9a3r1	0,20	0,12	0,33	0,30	0,16	0,31	0,49
Fis1	0,10	0,13	0,24	0,23	0,12	0,23	0,49
Akt1s1	0,16	0,07	0,25	0,21	0,11	0,23	0,50
Shisa5	0,33	0,14	0,52	0,42	0,23	0,47	0,50
Carhsp1	0,16	0,12	0,30	0,26	0,14	0,28	0,50
Kcnk5	0,25	0,18	0,44	0,41	0,21	0,43	0,50
Chmp1a	0,16	0,10	0,28	0,25	0,13	0,26	0,50
Mgst3	0,17	0,15	0,35	0,28	0,16	0,32	0,50
Gm12530	0,11	0,13	0,25	0,24	0,12	0,25	0,50
Slc39a7	0,20	0,16	0,39	0,33	0,18	0,36	0,50
Purg	0,13	0,08	0,22	0,19	0,10	0,21	0,50
Kctd17	0,14	0,11	0,27	0,24	0,13	0,25	0,50
Tor1aip2	0,10	0,11	0,22	0,20	0,10	0,21	0,50
Taf10	0,12	0,09	0,23	0,19	0,11	0,21	0,50
Akt1s1	0,18	0,08	0,30	0,24	0,13	0,27	0,50
4933404O12Rik	0,12	0,11	0,23	0,22	0,11	0,23	0,50
Rab3a	0,20	0,11	0,33	0,29	0,16	0,31	0,50
Synpo	0,12	0,09	0,21	0,21	0,10	0,21	0,50

Sphk2	0,27	0,16	0,51	0,36	0,22	0,43	0,50
2810013P06Rik	0,16	0,08	0,36	0,12	0,12	0,24	0,50
Arl1	0,15	0,19	0,39	0,29	0,17	0,34	0,50
Tor4a	0,12	0,15	0,32	0,21	0,13	0,26	0,50
Batf2	0,12	0,14	0,28	0,23	0,13	0,26	0,50
Il22ra1	0,15	0,17	0,35	0,29	0,16	0,32	0,50
Prr13	0,25	0,13	0,39	0,36	0,19	0,37	0,50
Mir135a-1	0,27	0,33	0,15	0,14	0,30	0,15	2,05
Ly6d	0,29	0,26	0,11	0,16	0,28	0,13	2,11
1700028B04Rik	0,43	0,17	0,13	0,15	0,30	0,14	2,15
Mir190b	0,54	0,00	0,20	0,05	0,27	0,12	2,17
Ly6g5b	0,34	0,09	0,06	0,14	0,21	0,10	2,21
Snora47	0,68	0,23	0,21	0,20	0,46	0,20	2,24
Snord42a	0,27	0,18	0,11	0,09	0,23	0,10	2,25
5830454E08Rik	0,34	0,02	0,08	0,07	0,18	0,08	2,28
Sapcd1	0,32	0,10	0,06	0,12	0,21	0,09	2,32
Rnu73b	0,27	0,21	0,17	0,04	0,24	0,10	2,34
BC033916	0,25	0,13	0,03	0,13	0,19	0,08	2,34
Csf3	0,30	0,05	0,08	0,07	0,18	0,07	2,39
1700092M07Rik	0,63	0,21	0,16	0,19	0,42	0,17	2,41
Mir32	0,54	0,36	0,20	0,18	0,45	0,19	2,42
Npvf	0,25	0,14	0,07	0,09	0,19	0,08	2,42
Mir196a-1	0,45	0,10	0,19	0,04	0,28	0,11	2,49
Mir148b	0,68	0,03	0,19	0,09	0,36	0,14	2,53
BC002163	0,31	0,04	0,09	0,05	0,18	0,07	2,53
Mir497	0,45	0,05	0,02	0,18	0,25	0,10	2,57
Zg16	0,41	0,06	0,09	0,09	0,24	0,09	2,62
Fabp4	0,18	0,15	0,05	0,07	0,17	0,06	2,66
Oxld1	0,38	0,18	0,09	0,12	0,28	0,10	2,76
Snord92	0,59	0,21	0,13	0,15	0,40	0,14	2,79
Mir1967	0,27	0,05	0,02	0,09	0,16	0,06	2,85
Mir1940	0,45	0,31	0,11	0,15	0,38	0,13	2,86

Calm4	0,26	0,07	0,04	0,08	0,17	0,06	2,91
Mir93	0,36	0,12	0,07	0,07	0,24	0,07	3,30
Mir449a	0,27	0,05	0,00	0,09	0,16	0,05	3,41
Mir181a-1	0,09	0,28	0,00	0,11	0,18	0,05	3,44
Mir3100	1,63	0,00	0,11	0,35	0,81	0,23	3,48
Mir22	0,00	0,29	0,02	0,06	0,15	0,04	3,77
Scarna3b	0,54	0,00	0,03	0,12	0,27	0,07	3,78
Olfr208	0,24	0,11	0,04	0,05	0,17	0,04	3,90
Mir181c	0,27	0,00	0,06	0,01	0,14	0,03	3,98
Mir3072	0,54	0,09	0,11	0,04	0,31	0,07	4,25
Snord73a	0,27	0,00	0,03	0,04	0,14	0,03	4,26
Spag11a	0,25	0,13	0,01	0,07	0,19	0,04	4,31
Rasd1	0,24	0,06	0,04	0,02	0,15	0,03	4,58
Mir16-2	0,99	0,31	0,07	0,20	0,65	0,14	4,73
Snord64	0,27	0,00	0,06	0,00	0,14	0,03	4,82
Mir1843	0,54	0,00	0,00	0,11	0,27	0,05	5,09
Prps1l1	0,22	0,08	0,02	0,04	0,15	0,03	5,16
Olfr282	0,26	0,05	0,03	0,03	0,15	0,03	5,21
C920025E04Rik	0,22	0,04	0,02	0,03	0,13	0,02	5,32
Mir145	0,41	0,26	0,00	0,12	0,33	0,06	5,36
Mir330	0,54	0,00	0,00	0,09	0,27	0,05	5,73
Mir759	0,54	0,00	0,04	0,05	0,27	0,04	6,39
Mir675	0,18	0,16	0,00	0,05	0,17	0,02	7,10
Dear1	0,19	0,04	0,00	0,03	0,12	0,02	7,11
Mir335	0,27	0,16	0,00	0,06	0,21	0,03	7,21
Mir877	0,54	0,00	0,07	0,00	0,27	0,04	7,23
Mir148a	0,90	0,26	0,13	0,02	0,58	0,08	7,51
Snord116l1	0,54	0,03	0,00	0,07	0,29	0,04	8,13
Ear5	0,16	0,11	0,01	0,03	0,13	0,02	8,29
Calm5	0,20	0,07	0,02	0,01	0,13	0,01	9,05
Snord45b	0,27	0,05	0,00	0,04	0,16	0,02	9,10
Krtap8-1	0,18	0,04	0,00	0,02	0,11	0,01	11,73

Olfr1324	0,22	0,05	0,01	0,02	0,13	0,01	11,73
Ube2dnl1	0,20	0,05	0,00	0,02	0,13	0,01	12,31
Olfr1413	0,20	0,01	0,00	0,02	0,11	0,01	14,34
Mir466	0,27	0,00	0,00	0,02	0,14	0,01	15,26
Mir1936	0,27	0,02	0,02	0,00	0,14	0,01	15,38
Olfr1509	0,22	0,09	0,00	0,02	0,15	0,01	16,14
Mir345	0,18	0,03	0,00	0,01	0,11	0,01	18,17
Olfr1048	0,16	0,05	0,00	0,01	0,11	0,00	29,89
Cypt8	0,41	0,02	0,01	0,00	0,21	0,01	36,33
Bhlha9	0,16	0,06	0,00	0,01	0,11	0,00	37,96
Cypt1	0,40	0,01	0,01	0,00	0,21	0,01	38,74
Hist1h3i	0,21	0,02	0,00	0,01	0,11	0,00	41,07
Vmn1r28	0,25	0,02	0,00	0,01	0,13	0,00	42,98
Mir122a	0,81	0,13	0,00	0,00	0,47	0,00	inf
Mir1955	0,81	0,10	0,00	0,00	0,46	0,00	inf
Mir378b	0,45	0,28	0,00	0,00	0,36	0,00	inf
Mir3108	0,54	0,05	0,00	0,00	0,30	0,00	inf
Snora19	0,54	0,00	0,00	0,00	0,27	0,00	inf
Mir200c	0,27	0,10	0,00	0,00	0,19	0,00	inf
Mir1942	0,00	0,36	0,00	0,00	0,18	0,00	inf
Dpm3	0,27	0,06	0,00	0,00	0,17	0,00	inf
Mir202	0,27	0,05	0,00	0,00	0,16	0,00	inf
H2afb1	0,21	0,08	0,00	0,00	0,15	0,00	inf
Olfr948	0,23	0,05	0,00	0,00	0,14	0,00	inf
Mir741	0,27	0,00	0,00	0,00	0,14	0,00	inf
Mir1938	0,27	0,00	0,00	0,00	0,14	0,00	inf
Snord2	0,27	0,00	0,00	0,00	0,14	0,00	inf
Mir125b-2	0,27	0,00	0,00	0,00	0,14	0,00	inf
Mir133a-1	0,27	0,00	0,00	0,00	0,14	0,00	inf
Mir684-1	0,27	0,00	0,00	0,00	0,14	0,00	inf
Mir137	0,27	0,00	0,00	0,00	0,14	0,00	inf
Mir127	0,00	0,26	0,00	0,00	0,13	0,00	inf

Olfr1049	0,19	0,03	0,00	0,00	0,11	0,00	inf

***Table* III** MiRs found in mice or in SY5Y cells, related to Fig. 4a

**miRs-mice**	**miRs-SY5Y**
miRLET7G	*miR1-2*
miRLET7D	*miR101-2*
miRLET7C-2	*miR103A1*
miR93	*miR103A2*
miR92-1	*miR103B1*
miR877	*miR103B2*
miR763	*miR105-2*
miR761	*miR106A*
miR759	*miR1178*
miR741	*miR1179*
miR706	*miR1193*
miR704	*miR1197*
miR700	*miR1200*
miR7-2	*miR1203*
miR684-1	*miR1205*
miR677	*miR1206*
miR675	*miR122*
miR540	*miR1224*
miR532	*miR1228*
miR511	*miR1231*
miR505	*miR1234*
miR497	*miR1236*
miR488	*miR1238*
miR468	*miR124-1*
miR466	*miR124-3*
miR449A	*miR1243*
miR434	*miR1244-1*
miR429	*miR1244-1*
miR3968	*miR1244-1*
miR378B	*miR1247*
miR345	*miR1252*
miR340	*miR1253*
miR335	*miR1257*
miR330	*miR1258*
miR33	*miR125A*
miR326	*miR125B1*
miR322	*miR126*
miR32	*miR1260B*
miR3109	*miR1262*
miR3108	*miR1264*
miR3103	*miR1265*
miR3100	*miR127*
miR31	*miR1270-1*

miR30A	*miR1275*
miR3095	*miR1276*
miR3089	*miR1278*
miR3076	*miR1282*
miR3075	*miR1283-1*
miR3072	*miR1283-2*
miR3069	*miR1287*
miR3063	*miR1289-2*
miR3061	*miR129-2*
miR3058	*miR1291*
miR29C	*miR1292*
miR26B	*miR1297*
miR26A-2	*miR1301*
miR26A-1	*miR1304*
miR22	*miR1305*
miR20A	*miR1306*
miR205	*miR130A*
miR202	*miR130B*
miR200C	*miR1322*
miR1982	*miR1323*
miR1981	*miR1324*
miR196A-1	*miR133A1*
miR1967	*miR133A2*
miR1961	*miR134*
miR1955	*miR135A1*
miR1942	*miR136*
miR1940	*miR137*
miR193B	*miR138-1*
miR1938	*miR138-2*
miR1936	*miR139*
miR1930	*miR144*
miR1929	*miR145*
miR191	*miR1468*
miR190B	*miR1469*
miR1904	*miR146A*
miR1843	*miR146B*
miR1839	*miR147A*
miR181C	*miR148A*
miR181A-1	*miR148B*
miR16-2	*miR149*
miR15A	*miR152*
miR148B	*miR153-1*
miR148A	*miR1538*
miR147	*miR1539*
miR145	*miR155*
miR139	*miR15A*

miR137	*miR15B*
miR135A-1	*miR17*
miR133A-1	*miR181A2*
miR127	*miR181B1*
miR125B-2	*miR181B2*
miR1247	*miR181C*
miR122A	*miR181D*
miR1192	*miR182*
miR1190	*miR1827*
miR10A	*miR183*
miR106A	*miR184*
miR103-1	*miR188*
	miR18A
	miR190A
	miR190B
	miR191
	miR1910
	miR1911
	miR1912
	miR1914
	miR193A
	miR193B
	miR194-1
	miR195
	miR196A1
	miR196B
	miR1973
	miR1976
	miR199A2
	miR199B
	miR19B1
	miR19B2
	miR200A
	miR200B
	miR202
	miR203
	miR204
	miR208B
	miR20B
	miR21
	miR210
	miR211
	miR2110
	miR2113
	miR2116
	miR2117

	miR216B	
	miR217	
	miR218-2	
	miR219-1	
	miR219-2	
	miR22	
	miR221	
	miR222	
	miR223	
	miR224	
	miR2276	
	miR2278	
	miR2392	
	miR23B	
	miR24-2	
	miR2682	
	miR26A2	
	miR27B	
	miR2861	
	miR2909	
	miR296	
	miR2964A	
	miR29A	
	miR29B1	
	miR302B	
	miR302D	
	miR3064	
	miR3065	
	miR30A	
	miR30B	
	miR30C1	
	miR30E	
	miR31	
	miR3115	
	miR3117	
	miR3119-2	
	miR3122	
	miR3126	
	miR3127	
	miR3128	
	miR3129	
	miR3130-2	
	miR3136	
	miR3139	
	miR3141	
	miR3142	

	miR3146	
	miR3148	
	miR3150A	
	miR3150B	
	miR3153	
	miR3158-2	
	miR3160-1	
	miR3160-2	
	miR3162	
	miR3163	
	miR3165	
	miR3167	
	miR3169	
	miR3173	
	miR3175	
	miR3178	
	miR3183	
	miR3184	
	miR3186	
	miR3187	
	miR3188	
	miR3189	
	miR3192	
	miR3194	
	miR3196	
	miR3197	
	miR3198-1	
	miR3199-1	
	miR3199-2	
	miR3201	
	miR320C1	
	miR323A	
	miR323B	
	miR324	
	miR326	
	miR328	
	miR329-1	
	miR329-2	
	miR330	
	miR331	
	miR335	
	miR337	
	miR338	
	miR339	
	miR342	
	miR34B	

	miR34C	
	miR3529	
	miR3545	
	miR3591	
	miR3605	
	miR3610	
	miR3613	
	miR3615	
	miR3618	
	miR362	
	miR3620	
	miR363	
	miR3646	
	miR3650	
	miR3652	
	miR3654	
	miR3655	
	miR3658	
	miR3659	
	miR365A	
	miR3663	
	miR3664	
	miR3666	
	miR3668	
	miR367	
	miR3671	
	miR3675	
	miR3675	
	miR3679	
	miR3684	
	miR3689A	
	miR3689D2	
	miR3689E	
	miR369	
	miR3691	
	miR3692	
	miR3714	
	miR371A	
	miR371B	
	miR373	
	miR374A	
	miR376A1	
	miR376A2	
	miR376C	
	miR377	
	miR378C	

	miR378D2	
	miR378F	
	miR380	
	miR381	
	miR382	
	miR3909	
	miR3910-1	
	miR3910-2	
	miR3912	
	miR3913-1	
	miR3913-2	
	miR3914-1	
	miR3914-2	
	miR3916	
	miR3917	
	miR3919	
	miR3921	
	miR3924	
	miR3925	
	miR3936	
	miR3938	
	miR3939	
	miR3940	
	miR3942	
	miR3943	
	miR3945	
	miR3960	
	miR3973	
	miR3976	
	miR411	
	miR412	
	miR423	
	miR4251	
	miR4255	
	miR4257	
	miR4258	
	miR4260	
	miR4261	
	miR4262	
	miR4263	
	miR4264	
	miR4265	
	miR4267	
	miR4268	
	miR4269	
	miR4270	

	miR4273	
	miR4275	
	miR4276	
	miR4277	
	miR4280	
	miR4281	
	miR4284	
	miR4285	
	miR4286	
	miR4287	
	miR4288	
	miR4290	
	miR4292	
	miR4294	
	miR4296	
	miR4297	
	miR4298	
	miR4299	
	miR4302	
	miR4305	
	miR4307	
	miR4308	
	miR4309	
	miR431	
	miR4312	
	miR4315-1	
	miR4316	
	miR432	
	miR4320	
	miR4322	
	miR4324	
	miR4325	
	miR4329	
	miR433	
	miR4417	
	miR4420	
	miR4423	
	miR4424	
	miR4429	
	miR4432	
	miR4436A	
	miR4436B1	
	miR4442	
	miR4443	
	miR4444-1	
	miR4446	

	miR4456	
	miR4457	
	miR4460	
	miR4464	
	miR4467	
	miR4468	
	miR4470	
	miR4472-1	
	miR4476	
	miR4478	
	miR448	
	miR4480	
	miR4482-1	
	miR4483	
	miR4484	
	miR4486	
	miR4488	
	miR4490	
	miR4491	
	miR4492	
	miR4493	
	miR4499	
	miR449A	
	miR449C	
	miR4500	
	miR4503	
	miR4508	
	miR450A1	
	miR450A2	
	miR4513	
	miR4514	
	miR4515	
	miR4518	
	miR4519	
	miR451A	
	miR451B	
	miR452	
	miR4521	
	miR4526	
	miR4529	
	miR4530	
	miR4531	
	miR4536-1	
	miR4540	
	miR455	
	miR4632	

	miR4633	
	miR4636	
	miR4637	
	miR4639	
	miR4640	
	miR4642	
	miR4644	
	miR4645	
	miR4647	
	miR4648	
	miR4650-1	
	miR4652	
	miR4654	
	miR4656	
	miR4657	
	miR4658	
	miR4660	
	miR4661	
	miR4666A	
	miR4667	
	miR4668	
	miR4669	
	miR4670	
	miR4671	
	miR4672	
	miR4673	
	miR4674	
	miR4677	
	miR4679-1	
	miR4679-2	
	miR4681	
	miR4682	
	miR4683	
	miR4684	
	miR4685	
	miR4686	
	miR4688	
	miR4689	
	miR4691	
	miR4693	
	miR4694	
	miR4695	
	miR4700	
	miR4704	
	miR4705	
	miR4707	

	miR4708	
	miR4709	
	miR4710	
	miR4711	
	miR4714	
	miR4716	
	miR4718	
	miR4719	
	miR4721	
	miR4723	
	miR4724	
	miR4725	
	miR4726	
	miR4727	
	miR4728	
	miR4731	
	miR4732	
	miR4733	
	miR4735	
	miR4736	
	miR4737	
	miR4738	
	miR4741	
	miR4742	
	miR4745	
	miR4748	
	miR4750	
	miR4751	
	miR4753	
	miR4757	
	miR4758	
	miR4759	
	miR4761	
	miR4763	
	miR4764	
	miR4767	
	miR4771-2	
	miR4773-2	
	miR4775	
	miR4778	
	miR4779	
	miR4782	
	miR4784	
	miR4786	
	miR4787	
	miR4788	

	miR4790	
	miR4793	
	miR4796	
	miR4797	
	miR4798	
	miR4799	
	miR4800	
	miR4802	
	miR4804	
	miR483	
	miR484	
	miR486	
	miR487A	
	miR487B	
	miR488	
	miR489	
	miR490	
	miR491	
	miR493	
	miR495	
	miR497	
	miR498	
	miR4999	
	miR5000	
	miR5001	
	miR5004	
	miR5008	
	miR5011	
	miR502	
	miR503	
	miR504	
	miR5047	
	miR506	
	miR507	
	miR508	
	miR5087	
	miR5088	
	miR5089	
	miR509-3	
	miR5090	
	miR5094	
	miR510	
	miR5100	
	miR511-1	
	miR515-2	
	miR516A1	

	miR516A2	
	miR516B1	
	miR517C	
	miR5186	
	miR5187	
	miR5189	
	miR518A1	
	miR518E	
	miR518F	
	miR5190	
	miR5191	
	miR5192	
	miR5193	
	miR5194	
	miR5197	
	miR519B	
	miR519C	
	miR519D	
	miR519E	
	miR520C	
	miR520E	
	miR520F	
	miR521-1	
	miR521-2	
	miR523	
	miR524	
	miR526A1	
	miR526A2	
	miR527	
	miR532	
	miR542	
	miR543	
	miR548A1	
	miR548AA2	
	miR548AD	
	miR548AR	
	miR548AR	
	miR548AR	
	miR548AV	
	miR548F2	
	miR548F4	
	miR548G	
	miR548J	
	miR548K	
	miR548U	
	miR548X2	

	miR549	
	miR550A1	
	miR550A2	
	miR551A	
	miR551B	
	miR553	
	miR554	
	miR555	
	miR556	
	miR557	
	miR5579	
	miR558	
	miR5582	
	miR5584	
	miR561	
	miR563	
	miR567	
	miR568	
	miR5687	
	miR5688	
	miR5690	
	miR5691	
	miR5692C2	
	miR5699	
	miR570	
	miR5700	
	miR5702	
	miR5703	
	miR5704	
	miR5707	
	miR5708	
	miR574	
	miR578	
	miR580	
	miR583	
	miR586	
	miR589	
	miR590	
	miR592	
	miR593	
	miR597	
	miR599	
	miR601	
	miR602	
	miR603	
	miR605	

	miR608	
	miR612	
	miR613	
	miR615	
	miR617	
	miR621	
	miR623	
	miR624	
	miR627	
	miR628	
	miR629	
	miR632	
	miR635	
	miR636	
	miR638	
	miR639	
	miR643	
	miR644A	
	miR644B	
	miR645	
	miR646	
	miR647	
	miR650	
	miR651	
	miR654	
	miR655	
	miR656	
	miR657	
	miR660	
	miR661	
	miR662	
	miR664	
	miR665	
	miR668	
	miR670	
	miR671	
	miR675	
	miR7-2	
	miR7-3	
	miR711	
	miR758	
	miR760	
	miR765	
	miR766	
	miR770	
	miR875	

		miR877	
		miR885	
		miR888	
		miR889	
		miR890	
		miR891A	
		miR891B	
		miR892B	
		miR9-2	
		miR921	
		miR92B	
		miR935	
		miR937	
		miR940	
		miR941-1	
		miR941-2	
		miR941-2	
		miR941-2	
		miR941-3	
		miR941-3	
		miR941-4	
		miR941-4	
		miR942	
		miR943	
		miR944	
		miR96	
		miR98	
		miRLET7A2	
		miRLET7B	
		miRLET7C	
		miRLET7D	
		miRLET7E	
		miRLET7F1	
		miRLET7G	

***Table* IV** MiRWalk Predicted mRNAs found in mice or in SY5Y cells, as well as common mRNAs found in mice and in SY5Y cells, related to Fig. 4b

Predicted mRNAs in mice	Predicted mRNAs in SY5Y	Common mRNAs
AACS	*ABAT*	*ABCB9*
AANAT	*ABCA12*	*ABHD6*
ABCA15	*ABCB7*	*ACADS*
ABCA3	*ABCB8*	*ACP2*
ABCA9	*ABCB9*	*ACTR1A*
ABCB6	*ABCC10*	*ACVR1*
ABCB9	*ABCC11*	*ACVR2A*
ABCC1	*ABCD1*	*ADCK4*
ABCC8	*ABCD3*	*AFAP1*
ABCC9	*ABCG5*	*AGBL5*
ABCD4	*ABHD12*	*AHDC1*
ABHD14A	*ABHD14B*	*AJAP1*
ABHD6	*ABHD6*	*AKR1D1*
ABL1	*ABI2*	*ALCAM*
ABL2	*ABI3*	*ALDH2*
ABLIM3	*ACACA*	*ALDH8A1*
ACAA2	*ACAD10*	*ALDOA*
ACADS	*ACAD9*	*ALOX5*
ACOT8	*ACADL*	*ANAPC13*
ACP2	*ACADS*	*ANAPC2*
ACP5	*ACBD6*	*ANKRA2*
ACP6	*ACP2*	*AP3B1*
ACSL4	*ACSBG1*	*AP4E1*
ACSM5	*ACSBG2*	*AQP11*
ACTC1	*ACSF3*	*AR*
ACTR1A	*ACTA1*	*ARFGAP2*
ACTR5	*ACTL7A*	*ARFIP2*
ACVR1	*ACTR1A*	*ARHGDIA*
ACVR1B	*ACVR1*	*ARID4A*
ACVR2A	*ACVR2A*	*ARID4B*
ACY1	*ADA*	*ARL11*
ADAM10	*ADAM19*	*ARL2*
ADAM11	*ADAM20*	*ARL6IP6*
ADAM12	*ADAM22*	*ARL8B*
ADAM15	*ADAM30*	*ARMCX2*
ADAM1B	*ADAM9*	*ARPC5*
ADAM2	*ADAMTS8*	*ARRB1*
ADAM6	*ADAMTSL1*	*ASB1*
ADAMTS1	*ADCK1*	*ASB3*
ADAMTS10	*ADCK2*	*ASH1L*
ADAMTS19	*ADCK4*	*ASH2L*

ADAMTS4	*ADCY6*	*ATG12*
ADAMTSL2	*ADCY7*	*ATG4B*
ADAR	*ADH1A*	*ATG9A*
ADCK4	*ADH1B*	*ATP8A1*
ADCY3	*ADH1C*	*ATXN2*
ADCY5	*ADNP2*	*ATXN7L2*
ADCYAP1R1	*ADO*	*B3GNT7*
ADFP	*ADORA1*	*B3GNTL1*
ADI1	*ADRA2A*	*BAG3*
ADIPOQ	*ADRB2*	*BATF3*
ADORA2B	*ADRBK1*	*BBC3*
ADRA1D	*ADRBK2*	*BCL2*
ADRA2B	*AFAP1*	*BCL9L*
AF529169	*AFF4*	*BECN1*
AFAP1	*AGBL2*	*BGN*
AGBL5	*AGBL5*	*BIRC5*
AGPAT6	*AGK*	*BRI3*
AHDC1	*AGTPBP1*	*BRSK2*
AHNAK	*AGTR1*	*BTG2*
AHSA2	*AGTRL1*	*C1QTNF5*
AHSG	*AGXT2L1*	*C1QTNF7*
AI118078	*AHDC1*	*CABLES2*
AI428936	*AHNAK2*	*CADPS*
AI429214	*AHR*	*CALM1*
AIFM2	*AIPL1*	*CAMK2B*
AIFM3	*AJAP1*	*CANT1*
AJAP1	*AKAP13*	*CARS*
AKAP14	*AKR1B1*	*CBFB*
AKAP6	*AKR1D1*	*CBY1*
AKAP8	*ALCAM*	*CCDC120*
AKAP9	*ALDH2*	*CCDC28A*
AKR1A4	*ALDH8A1*	*CCDC46*
AKR1B7	*ALDOA*	*CCDC88C*
AKR1D1	*ALDOC*	*CCDC96*
AKT1	*ALG10*	*CCM2*
AKT2	*ALG2*	*CCND2*
ALCAM	*ALG6*	*CCNE1*
ALDH2	*ALKBH3*	*CCNE2*
ALDH8A1	*ALOX12*	*CCNJL*
ALDH9A1	*ALOX5*	*CD40*
ALDOA	*ALPI*	*CD48*
ALG12	*ALPP*	*CD8A*
ALOX12B	*ALPPL2*	*CD93*
ALOX5	*ALX3*	*CD97*
ALS2CR12	*AMZ1*	*CDC37*
AMACR	*ANAPC11*	*CDC37L1*

AMIGO3	*ANAPC13*	*CDK8*
AMOTL1	*ANAPC2*	*CELSR3*
AMPH	*ANGEL1*	*CHD9*
ANAPC1	*ANGPT2*	*CHEK1*
ANAPC10	*ANK2*	*CHGA*
ANAPC13	*ANKHD1*	*CHIA*
ANAPC2	*ANKRA2*	*CHMP1A*
ANKMY1	*ANKRD12*	*CHMP7*
ANKRA2	*ANKRD23*	*CHORDC1*
ANKRD26	*ANKRD29*	*CIB2*
ANKRD34B	*ANKRD39*	*CIDEB*
ANKRD34C	*ANKRD43*	*CLDN1*
ANKRD40	*ANKRD45*	*CLDN2*
ANKRD49	*ANKRD53*	*CLN8*
ANKRD58	*ANKRD55*	*CLOCK*
ANKRD7	*ANKS4B*	*CLRN1*
ANKS3	*ANKS6*	*CNN1*
AOX3	*ANTXR2*	*CNOT6*
AP1S3	*ANXA13*	*CNP*
AP2A2	*ANXA4*	*CNTN2*
AP3B1	*ANXA6*	*CNTNAP4*
AP4B1	*AP1B1*	*COG2*
AP4E1	*AP2A1*	*COL13A1*
APAF1	*AP3B1*	*COMMD9*
APBH	*AP3B2*	*COPS2*
APITD1	*AP4E1*	*CORO7*
APLN	*APBB1IP*	*CPNE8*
APOA5	*APBB2*	*CPSF6*
AQP11	*APCDD1L*	*CRTAC1*
AQP4	*APH1B*	*CRYGN*
AQP5	*APOA2*	*CSDA*
AR	*APOA4*	*CSE1L*
ARF3	*APOBEC2*	*CSMD1*
ARFGAP1	*APOBEC3C*	*CST6*
ARFGAP2	*APOH*	*CTDSP2*
ARFIP2	*APOL6*	*CTHRC1*
ARHGAP1	*APPL2*	*CTTNBP2NL*
ARHGAP19	*AQP11*	*CUEDC1*
ARHGAP25	*AQP9*	*CXCL9*
ARHGAP28	*AR*	*CXCR7*
ARHGAP4	*ARCN1*	*CYP24A1*
ARHGAP5	*ARFGAP2*	*D2HGDH*
ARHGDIA	*ARFIP2*	*DAAM1*
ARHGEF2	*ARFRP1*	*DBC1*
ARID4A	*ARG2*	*DDX4*
ARID4B	*ARHGAP11A*	*DEDD*

ARL11	*ARHGAP15*	*DEF8*
ARL15	*ARHGAP18*	*DEPDC5*
ARL2	*ARHGAP22*	*DET1*
ARL6IP1	*ARHGAP26*	*DGKQ*
ARL6IP6	*ARHGAP27*	*DGKZ*
ARL8B	*ARHGDIA*	*DHRS13*
ARMC6	*ARHGDIB*	*DIXDC1*
ARMCX2	*ARHGEF12*	*DMRT2*
ARPC5	*ARID4A*	*DMRTA1*
ARPP19	*ARID4B*	*DMXL1*
ARPP21	*ARL10*	*DNAJA1*
ARRB1	*ARL11*	*DNAJC7*
ARRDC3	*ARL2*	*DOCK6*
ASAH3	*ARL6IP6*	*DOCK7*
ASB1	*ARL8B*	*DOCK8*
ASB3	*ARMC1*	*DPP3*
ASB6	*ARMC4*	*DR1*
ASCL3	*ARMCX1*	*DUSP10*
ASH1L	*ARMCX2*	*DUSP2*
ASH2L	*ARMCX3*	*DUSP8*
ASNA1	*ARPC5*	*EDARADD*
ASPRV1	*ARPC5L*	*EDNRA*
ASZ1	*ARPP-19*	*EED*
ATG12	*ARPP-21*	*EFCAB5*
ATG4B	*ARRB1*	*EHD1*
ATG9A	*ARSF*	*EIF2B2*
ATP11A	*ASB1*	*EIF2B5*
ATP5E	*ASB2*	*EIF3A*
ATP6V0D1	*ASB3*	*EIF3B*
ATP6V1G3	*ASB4*	*EIF3G*
ATP7A	*ASCC2*	*ELAVL2*
ATP8A1	*ASCC3*	*EME1*
ATP8B3	*ASH1L*	*EML5*
ATP9B	*ASH2L*	*EMX1*
ATRNL1	*ASNSD1*	*EPC2*
ATXN10	*ASXL1*	*EPHA1*
ATXN1L	*ASXL2*	*EPHA7*
ATXN2	*ATAD1*	*ERLIN1*
ATXN7L2	*ATF1*	*ERRFI1*
AU019823	*ATG12*	*ESCO1*
AU021034	*ATG2A*	*ESRRA*
AU022870	*ATG4B*	*ETFB*
AURKAIP1	*ATG5*	*ETS1*
AURKB	*ATG7*	*EVI5L*
AW146020	*ATG9A*	*EVPL*
AW551984	*ATG9B*	*FA2H*

AXIN1	ATN1	FAH
AXL	ATOH8	FAP
B230217C12RIK	ATP10A	FBXW5
B2M	ATP13A2	FDFT1
B3GALNT2	ATP1A2	FECH
B3GALTL	ATP1B4	FGGY
B3GAT1	ATP4B	FLVCR2
B3GNT5	ATP5G2	FMO1
B3GNT6	ATP5SL	FNDC4
B3GNT7	ATP6AP2	FNTA
B3GNTL1	ATP6V0A2	FOXD1
B4GALT1	ATP6V0B	FOXJ2
B4GALT3	ATP6V1A	FSCN1
B4GALT6	ATP6V1B2	FUK
B4GALT7	ATP8A1	FURIN
BAG3	ATPBD1C	FUT8
BAIAP2L2	ATPIF1	FXC1
BAMBI	ATXN2	From
BANF2	ATXN7L2	GABPB2
BAP1	AUTS2	GABRA3
BASP1	AVEN	GABRG3
BAT1A	AZU1	GADD45A
BATF3	B3GAT3	GALNT1
BBC3	B3GNT7	GALNT3
BC003266	B3GNTL1	GALNT7
BC004004	B4GALNT3	GBL
BC017612	B4GALNT4	GIN1
BC017643	B4GALT2	GJB3
BC017647	BAALC	GJB5
BC018465	BAG2	GLDC
BC021785	BAG3	GMFB
BC021891	BAG4	GNAI2
BC023829	BAHCC1	GNE
BC024479	BAI3	GOLGA1
BC046331	BAIAP2L1	GPATCH3
BC049715	BANF1	GPM6A
BC051019	BAT2D1	GPR137B
BC054059	BAT4	GPR26
BC066028	BATF3	GPR64
BC068281	BAZ1A	GPSM3
BCCIP	BAZ1B	GPT2
BCL2	BBC3	GPX7
BCL6	BCAN	GRB7
BCL9L	BCAR1	GRM3
BDH1	BCL2	GSTO1
BECN1	BCL2A1	GTF3C1

BGLAP-RS1	*BCL2L1*	*GZF1*
BGLAP2	*BCL2L11*	*H3F3B*
BGN	*BCL2L15*	*HCRT*
BHMT	*BCL7B*	*HDAC8*
BIN1	*BCL9L*	*HELZ*
BIRC5	*BCS1L*	*HERPUD2*
BLZF1	*BECN1*	*HEY1*
BMP1	*BEST3*	*HIST2H2BE*
BMX	*BEST4*	*HLX*
BMYC	*BET1*	*HMOX1*
BNIP2	*BGN*	*HOXA1*
BNIPL	*BIRC5*	*HPS4*
BPIL3	*BLK*	*HPSE2*
BRI3	*BLVRB*	*HSD3B2*
BRMS1L	*BMPR2*	*HSPA1B*
BRP16	*BMS1*	*HUS1*
BRPF1	*BNC2*	*IFT140*
BRSK2	*BOLA3*	*IHPK3*
BRUNOL4	*BRAF*	*IKZF3*
BRWD3	*BRAP*	*IL17RD*
BSCL2	*BRD1*	*IL1A*
BSPRY	*BRF2*	*IL21R*
BTBD10	*BRI3*	*IL8RB*
BTG2	*BRMS1*	*INVS*
BTN1A1	*BRP44L*	*IRF2BP1*
BUTR1	*BRPF3*	*ITGA11*
BZW2	*BRS3*	*ITPR2*
C1QA	*BRSK1*	*ITSN2*
C1QL1	*BRSK2*	*JAG1*
C1QL3	*BSND*	*KCNA1*
C1QTNF2	*BTBD11*	*KCNE1L*
C1QTNF3	*BTBD9*	*KCNF1*
C1QTNF5	*BTG2*	*KCNIP4*
C1QTNF7	*BTG4*	*KCNJ11*
C1QTNF9	*BTLA*	*KCNK3*
C330005M16RIK	*C10ORF12*	*KCNN3*
CAB39L	*C10ORF125*	*KCNQ5*
CABIN1	*C10ORF26*	*KCTD13*
CABLES2	*C10ORF4*	*KCTD5*
CABP5	*C10ORF55*	*KDELR2*
CACNB1	*C10ORF63*	*KDELR3*
CACNG1	*C10ORF65*	*KIF5C*
CADM2	*C10ORF72*	*KLC1*
CADPS	*C10ORF81*	*KLC3*
CALB1	*C10ORF82*	*KLHDC8B*
CALCA	*C10ORF83*	*KLHL10*

CALCOCO1	*C10ORF92*	*KRR1*
CALD1	*C11ORF10*	*KRT34*
CALM1	*C11ORF17*	*LAMB2*
CALML3	*C11ORF30*	*LAMC3*
CALY	*C11ORF41*	*LCK*
CAMK2B	*C11ORF42*	*LGI1*
CANT1	*C11ORF61*	*LGI3*
CAP2	*C11ORF68*	*LHCGR*
CAPG	*C11ORF75*	*LHX8*
CAR10	*C12ORF32*	*LIMCH1*
CAR14	*C12ORF4*	*LIPE*
CAR7	*C12ORF52*	*LIPI*
CARD10	*C12ORF56*	*LMBR1L*
CARS	*C12ORF57*	*LPO*
CASKIN2	*C12ORF59*	*LRRC52*
CASP1	*C13ORF16*	*LRRN2*
CASP9	*C13ORF23*	*LYSMD2*
CASS4	*C13ORF3*	*MAF1*
CBFB	*C14ORF149*	*MAFB*
CBL	*C14ORF181*	*MAGI3*
CBLC	*C14ORF2*	*MAN1B1*
CBX1	*C14ORF28*	*MAP3K12*
CBY1	*C14ORF37*	*MAP3K14*
CC2D1B	*C14ORF39*	*MAP3K7IP3*
CCBP2	*C15ORF17*	*MAPKAPK2*
CCDC100	*C15ORF2*	*MARCH4*
CCDC101	*C15ORF48*	*MAT2A*
CCDC11	*C16ORF3*	*MBOAT1*
CCDC113	*C16ORF46*	*MED8*
CCDC117	*C16ORF57*	*MED9*
CCDC120	*C16ORF63*	*METTL9*
CCDC122	*C16ORF73*	*MFAP4*
CCDC123	*C16ORF84*	*MGAT5B*
CCDC144B	*C17ORF56*	*MICAL1*
CCDC28A	*C17ORF57*	*MKRN3*
CCDC38	*C17ORF58*	*MLXIP*
CCDC46	*C17ORF59*	*MMD*
CCDC49	*C18ORF22*	*MON1B*
CCDC59	*C18ORF25*	*MPDU1*
CCDC64	*C18ORF34*	*MPP1*
CCDC71	*C18ORF62*	*MREG*
CCDC80	*C19ORF10*	*MRPL18*
CCDC85A	*C19ORF22*	*MRPS2*
CCDC86	*C19ORF25*	*MTDH*
CCDC88C	*C19ORF36*	*MTFR1*
CCDC90B	*C19ORF40*	*MTTP*

CCDC96	*C19ORF50*	*MYBL2*
CCL1	*C19ORF56*	*MYH3*
CCL9	*C1GALT1*	*NAB2*
CCM2	*C1ORF102*	*NADK*
CCND2	*C1ORF110*	*NAGPA*
CCNE1	*C1ORF113*	*NAT14*
CCNE2	*C1ORF122*	*NCLN*
CCNJL	*C1ORF129*	*NCOA2*
CCNY	*C1ORF135*	*NCOR2*
CCT2	*C1ORF142*	*NDUFA4*
CD200R1	*C1ORF144*	*NEDD1*
CD209A	*C1ORF150*	*NEFL*
CD2BP2	*C1ORF163*	*NEURL*
CD36	*C1ORF164*	*NEUROD1*
CD3D	*C1ORF173*	*NEUROD2*
CD40	*C1ORF19*	*NEUROG3*
CD48	*C1ORF21*	*NFASC*
CD79A	*C1ORF26*	*NFATC3*
CD81	*C1ORF27*	*NLRX1*
CD86	*C1ORF54*	*NMI*
CD8A	*C1ORF69*	*NOL4*
CD93	*C1ORF92*	*NPAS4*
CD97	*C1QB*	*NPEPL1*
CDC123	*C1QTNF5*	*NPR2*
CDC25A	*C1QTNF7*	*NR2C2AP*
CDC2L1	*C20ORF108*	*NRBP1*
CDC37	*C20ORF114*	*NT5DC2*
CDC37L1	*C20ORF118*	*NT5E*
CDC42EP2	*C20ORF134*	*NUMA1*
CDC42SE2	*C20ORF186*	*NXPH2*
CDCA5	*C20ORF20*	*OMG*
CDCA7	*C20ORF200*	*OPRS1*
CDCA8	*C20ORF26*	*ORC3L*
CDH12	*C20ORF29*	*ORMDL3*
CDH15	*C20ORF43*	*OSGEP*
CDH16	*C20ORF46*	*OSGEPL1*
CDH20	*C20ORF96*	*OSGIN2*
CDH26	*C21ORF55*	*P4HA2*
CDH7	*C21ORF56*	*PACS1*
CDK5RAP1	*C21ORF62*	*PAWR*
CDK5RAP2	*C21ORF70*	*PDC*
CDK8	*C22ORF28*	*PDCD6*
CDK9	*C22ORF29*	*PDE10A*
CDKL2	*C22ORF32*	*PDE1B*
CDS2	*C2CD2*	*PDE4A*
CEACAM10	*C2ORF15*	*PDLIM3*

CELSR3	*C2ORF42*	*PDSS1*
CENTG2	*C2ORF60*	*PDZD4*
CEP152	*C2ORF61*	*PGAM2*
CFHR1	*C2ORF7*	*PGD*
CFTR	*C3ORF10*	*PGM1*
CGA	*C3ORF25*	*PGM2*
CHCHD8	*C3ORF37*	*PHOX2B*
CHD7	*C3ORF58*	*PIGM*
CHD9	*C4ORF15*	*PIP*
CHEK1	*C4ORF34*	*PIP5K1B*
CHEK2	*C5ORF13*	*PITPNM2*
CHGA	*C5ORF23*	*PLA2G2F*
CHGB	*C5ORF39*	*PLAG1*
CHIA	*C5ORF42*	*PLCD1*
CHMP1A	*C6ORF108*	*PLEKHA6*
CHMP7	*C6ORF120*	*PLXNB1*
CHORDC1	*C6ORF154*	*PMF1*
CHRAC1	*C6ORF201*	*PMPCB*
CHRNA10	*C6ORF203*	*PNO1*
CHRNB2	*C6ORF223*	*PNPLA2*
CHRNB4	*C6ORF47*	*PNPLA6*
CHRNE	*C6ORF57*	*POLQ*
CHST1	*C6ORF60*	*POMC*
CHUK	*C6ORF66*	*POU4F2*
CIAO1	*C7ORF20*	*PPAP2A*
CIB1	*C7ORF27*	*PPARGC1B*
CIB2	*C7ORF30*	*PPID*
CIDEA	*C7ORF33*	*PPIH*
CIDEB	*C7ORF41*	*PPM1E*
CLCN7	*C7ORF42*	*PPP2R1A*
CLCNKB	*C7ORF43*	*PPP5C*
CLDN1	*C7ORF49*	*PPT1*
CLDN2	*C7ORF52*	*PRICKLE1*
CLDND1	*C8A*	*PRKAG2*
CLEC14A	*C8ORF31*	*PRKCZ*
CLEC1A	*C8ORF32*	*PRKG1*
CLEC1B	*C8ORF44*	*PROZ*
CLEC2E	*C8ORF53*	*PRR16*
CLINT1	*C8ORF55*	*PRTG*
CLN8	*C8ORF58*	*PSD2*
CLOCK	*C8ORF76*	*PSKH1*
CLRN1	*C8ORF79*	*PSMD13*
CLTB	*C9ORF125*	*PTCD3*
CLYBL	*C9ORF128*	*PTH*
CMC1	*C9ORF150*	*PTPN13*
CNFN	*C9ORF18*	*PTPN18*

CNGA2	*C9ORF19*	*PTPN2*
CNGA4	*C9ORF24*	*PYGO2*
CNGB3	*C9ORF32*	*R3HDM1*
CNIH2	*C9ORF47*	*RAB11FIP5*
CNN1	*C9ORF61*	*RAB15*
CNOT6	*C9ORF7*	*RAB32*
CNP	*C9ORF72*	*RAB34*
CNTF	*CA14*	*RAB4B*
CNTN2	*CA9*	*RAB7L1*
CNTNAP2	*CABLES2*	*RABEPK*
CNTNAP4	*CABP1*	*RABL4*
COASY	*CACNB4*	*RAVER2*
COG2	*CADPS*	*RBM15*
COL10A1	*CALB2*	*RBM6*
COL13A1	*CALCR*	*RBMS2*
COL1A1	*CALM1*	*RCN3*
COL4A1	*CALML6*	*RCOR2*
COL4A4	*CALR*	*RDH12*
COL9A2	*CALU*	*REEP3*
COLEC10	*CAMK2B*	*RELL1*
COMMD4	*CAMK2D*	*RFTN2*
COMMD8	*CAMK2N1*	*RFXDC1*
COMMD9	*CAMK4*	*RGMA*
COMTD1	*CAMKK1*	*RGS10*
COPA	*CAMSAP1L1*	*RGS7BP*
COPS2	*CAND1*	*RHEBL1*
COQ2	*CANT1*	*RHOBTB1*
CORIN	*CAPN3*	*RHOD*
CORO1B	*CAPN6*	*RNF135*
CORO1C	*CARHSP1*	*RNF138*
CORO7	*CARS*	*RNF219*
COTL1	*CARS2*	*RNF25*
COX11	*CASKIN1*	*RNGTT*
COX5B	*CASP3*	*RPA2*
COX6A2	*CASQ1*	*RPL37A*
COX6B2	*CASZ1*	*RPP25*
COX6C	*CAT*	*RPP40*
CPEB1	*CATSPER1*	*RPS6KA5*
CPLX3	*CBARA1*	*RPS6KB2*
CPNE7	*CBFA2T3*	*RRAD*
CPNE8	*CBFB*	*RRAS*
CPSF1	*CBR3*	*RSF1*
CPSF3	*CBX6*	*RSPO2*
CPSF4L	*CBX7*	*RTN4R*
CPSF6	*CBY1*	*RUNX1T1*
CPT1B	*CC2D2B*	*S100A3*

CPXCR1	*CCDC120*	*S100A5*
CPXM2	*CCDC126*	*SAP30BP*
CRABP1	*CCDC134*	*SATB2*
CRADD	*CCDC135*	*SBF1*
CRELD2	*CCDC19*	*SCEL*
CRISPLD2	*CCDC28A*	*SCN1A*
CRLF2	*CCDC28B*	*SCNN1G*
CRTAC1	*CCDC3*	*SDCBP2*
CRTC2	*CCDC46*	*SEC22C*
CRYGA	*CCDC47*	*SEC23B*
CRYGB	*CCDC52*	*SEMA3A*
CRYGF	*CCDC56*	*SEMA6D*
CRYGN	*CCDC57*	*SERP1*
CRYL1	*CCDC6*	*SERPINB8*
CSDA	*CCDC65*	*SERPINF2*
CSDC2	*CCDC81*	*SESN1*
CSE1L	*CCDC83*	*SETD7*
CSF1R	*CCDC88C*	*SFRS11*
CSF2RA	*CCDC96*	*SFXN2*
CSF2RB	*CCL15*	*SGCG*
CSF3R	*CCL19*	*SH2D2A*
CSMD1	*CCL23*	*SH3BP5*
CSN1S2A	*CCL25*	*SIN3A*
CSNK1G1	*CCM2*	*SKIV2L2*
CSNK1G2	*CCNA1*	*SLC1A5*
CSPG4	*CCND2*	*SLC22A18*
CST6	*CCNE1*	*SLC25A10*
CTDSP2	*CCNE2*	*SLC25A5*
CTHRC1	*CCNG2*	*SLC29A1*
CTPS	*CCNJL*	*SLC2A1*
CTR9	*CCNK*	*SLC35A5*
CTSW	*CCR10*	*SLC35B3*
CTTNBP2NL	*CCR4*	*SLC36A1*
CUEDC1	*CCT7*	*SLC37A1*
CUL5	*CD177*	*SLC37A3*
CXADR	*CD180*	*SLC38A7*
CXCL3	*CD1A*	*SLC39A5*
CXCL9	*CD1D*	*SLC41A2*
CXCR3	*CD2*	*SLC41A3*
CXCR5	*CD207*	*SLC43A2*
CXCR7	*CD300C*	*SLC46A3*
CXXC4	*CD300LG*	*SLC5A11*
CYC1	*CD33*	*SLC6A4*
CYFIP2	*CD3E*	*SLC6A8*
CYP24A1	*CD4*	*SLC7A10*
CYP2C38	*CD40*	*SMC6*

CYP2C54	*CD48*	*SMPD1*
CYP2D22	*CD55*	*SNAI1*
CYP39A1	*CD6*	*SNAPIN*
CYP3A13	*CD70*	*SNCG*
CYPT2	*CD72*	*SNF1LK*
CYTL1	*CD80*	*SNRK*
D0H4S114	*CD83*	*SNX2*
D16H22S680E	*CD8A*	*SNX25*
D17H6S56E-3	*CD93*	*SNX33*
D18ERTD653E	*CD97*	*SOCS1*
D19ERTD386E	*CDC14A*	*SOCS3*
D2ERTD750E	*CDC14B*	*SOS1*
D2HGDH	*CDC37*	*SPAG7*
D2WSU81E	*CDC37L1*	*SPATA16*
D330028D13RIK	*CDC42*	*SPEN*
D330038O06RIK	*CDH13*	*SPON1*
D4BWG0951E	*CDK5RAP3*	*SRFBP1*
D630042P16RIK	*CDK8*	*SRP72*
DAAM1	*CEACAM4*	*SRPX2*
DAAM2	*CEACAM6*	*SSTR1*
DACH1	*CELSR3*	*ST3GAL3*
DAF2	*CENPO*	*STAG3*
DAK	*CENPT*	*STC1*
DAP3	*CENTA2*	*STK17B*
DAPK2	*CEP76*	*STK19*
DAPP1	*CH25H*	*STOX2*
DAZL	*CHD9*	*SUOX*
DBC1	*CHEK1*	*SURF4*
DBP	*CHGA*	*SYNGR3*
DCPS	*CHI3L1*	*SYT1*
DCTD	*CHIA*	*SYVN1*
DCXR	*CHMP1A*	*TACC2*
DDB2	*CHMP6*	*TASP1*
DDI1	*CHMP7*	*TBC1D12*
DDIT4L	*CHORDC1*	*TBC1D19*
DDX1	*CHPT1*	*TCF25*
DDX21	*CHRNA4*	*TCP1*
DDX4	*CHRNA5*	*TDRD7*
DDX54	*CHST2*	*TGIF2*
DEAF1	*CHST3*	*THBD*
DEDD	*CHST4*	*THUMPD1*
DEF8	*CHST9*	*TJAP1*
DEFB25	*CIAPIN1*	*TK2*
DEFB30	*CIB2*	*TKTL1*
DEFB50	*CIDEB*	*TM9SF3*
DENND2C	*CIDEC*	*TMEFF1*

DEPDC5	*CIITA*	*TMEM121*
DERL1	*CINP*	*TMEM128*
DET1	*CISH*	*TMEM177*
DEXI	*CITED1*	*TMEM55A*
DGCR6	*CLDN1*	*TMEM80*
DGCR8	*CLDN12*	*TNFAIP1*
DGKQ	*CLDN18*	*TNFSF10*
DGKZ	*CLDN2*	*TNNC2*
DGUOK	*CLDN3*	*TNRC4*
DHRS13	*CLDN9*	*TOMM34*
DHRS9	*CLEC12B*	*TOR1AIP1*
DHX35	*CLEC4D*	*TRIM6*
DIABLO	*CLEC4F*	*TRO*
DIP2B	*CLIP4*	*TSHB*
DISP2	*CLK4*	*TSN*
DIXDC1	*CLMN*	*TSP50*
DLL1	*CLN5*	*TSPYL2*
DLX3	*CLN6*	*TSPYL4*
DMN	*CLN8*	*TUBA1A*
DMRT2	*CLOCK*	*TUT1*
DMRTA1	*CLRN1*	*TXNDC12*
DMWD	*CLSTN1*	*TXNDC13*
DMXL1	*CMTM1*	*TXNDC4*
DNAJA1	*CMTM6*	*UACA*
DNAJA2	*CMTM7*	*UBAC1*
DNAJA3	*CNN1*	*UBN1*
DNAJC15	*CNOT6*	*UGP2*
DNAJC7	*CNOT7*	*UHRF1BP1*
DNAJC9	*CNP*	*UMOD*
DNASE1L3	*CNTD1*	*UNC119B*
DNM1L	*CNTFR*	*UNC93B1*
DNMBP	*CNTN2*	*UNK*
DNTTIP2	*CNTN4*	*UQCRC1*
DOCK6	*CNTNAP4*	*USP14*
DOCK7	*COCH*	*USP32*
DOCK8	*COG2*	*USP48*
DOHH	*COG3*	*UTP15*
DOS	*COIL*	*VAMP8*
DPAGT1	*COL11A1*	*VARS*
DPH5	*COL13A1*	*VAT1*
DPP3	*COL24A1*	*VIT*
DPP4	*COL2A1*	*VPS24*
DPP7	*COL5A1*	*VPS33B*
DPT	*COL6A3*	*VPS39*
DPY19L3	*COL9A3*	*VTI1B*
DPY30	*COMMD9*	*WARS2*

DPYS	*COPS2*	*WBP11*
DR1	*COPS4*	*WDR59*
DRD1A	*CORO7*	*WDR91*
DRD2	*CPA3*	*WDR92*
DSEL	*CPE*	*WDTC1*
DTL	*CPNE8*	*WNT8A*
DTNA	*CPO*	*WRNIP1*
DTX2	*CPSF3L*	*WSCD2*
DTYMK	*CPSF6*	*XYLB*
DUOXA2	*CRAMP1L*	*YWHAB*
DUSP10	*CRB1*	*ZBTB7B*
DUSP16	*CRB3*	*ZC3H3*
DUSP19	*CRHBP*	*ZCCHC9*
DUSP2	*CRIP2*	*ZDHHC16*
DUSP4	*CRISP1*	*ZFHX4*
DUSP8	*CRK*	*ZFP36L1*
DYNC2H1	*CRLS1*	*ZFP91*
DYNLL2	*CRMP1*	*ZNHIT3*
DYNLT3	*CROCC*	*ZNRF1*
DYRK1B	*CRTAC1*	*ZYX*
E030010A14RIK	*CRYBA1*	
E130203B14RIK	*CRYGN*	
E130306D19RIK	*CRYM*	
E2F5	*CSDA*	
EAF1	*CSE1L*	
EAF2	*CSMD1*	
EAPA2	*CSMD2*	
EBAG9	*CSNK2A2*	
EBI2	*CSPG5*	
EBPL	*CST11*	
ECD	*CST6*	
ECHDC2	*CST9L*	
ECHS1	*CSTL1*	
EDARADD	*CTAG2*	
EDN2	*CTDSP2*	
EDNRA	*CTHRC1*	
EDNRB	*CTNNA3*	
EED	*CTNNBIP1*	
EEF1G	*CTRC*	
EEF2	*CTSA*	
EEF2K	*CTSC*	
EFCAB1	*CTSF*	
EFCAB2	*CTSL1*	
EFCAB5	*CTSO*	
EFCBP2	*CTTNBP2NL*	
EFNA3	*CUEDC1*	

EFR3A	*CUL2*		
EG214403	*CUL3*		
EHD1	*CX3CL1*		
EIF2AK4	*CX3CR1*		
EIF2B2	*CXCL9*		
EIF2B5	*CXCR7*		
EIF2C2	*CXORF23*		
EIF2S1	*CXORF58*		
EIF3A	*CYB561*		
EIF3B	*CYB5D1*		
EIF3G	*CYBA*		
EIF4E3	*CYBRD1*		
EIF4G1	*CYFIP1*		
EIF4G2	*CYHR1*		
EIF4H	*CYP24A1*		
ELA1	*CYP2C9*		
ELA2	*CYP2E1*		
ELAVL1	*CYP2S1*		
ELAVL2	*CYYR1*		
ELAVL4	*D2HGDH*		
ELF4	*D4S234E*		
ELK3	*DAAM1*		
ELL	*DACT1*		
ELL2	*DAD1*		
ELMO2	*DAPK1*		
ELOVL4	*DAPK3*		
ELOVL7	*DARC*		
ELP4	*DAZAP2*		
ELTD1	*DBC1*		
EMB	*DBF4B*		
EME1	*DBNL*		
EMILIN3	*DCBLD2*		
EML5	*DCUN1D5*		
EMX1	*DDEF1*		
ENAH	*DDN*		
ENO2	*DDX10*		
ENPEP	*DDX39*		
ENPP1	*DDX4*		
ENSA	*DDX51*		
ENTPD7	*DDX52*		
EPC2	*DDX53*		
EPDR1	*DDX56*		
EPHA1	*DDX58*		
EPHA7	*DEDD*		
EPHA8	*DEF8*		
EPN2	*DEFA6*		

EPN3	*DEFB1*		
EPO	*DEFB127*		
EPS15	*DENND4A*		
EPS8L1	*DEPDC1B*		
EPYC	*DEPDC4*		
ERC2	*DEPDC5*		
ERCC4	*DERL3*		
ERCC6	*DET1*		
ERCC8	*DFFB*		
ERG	*DGKE*		
ERGIC3	*DGKH*		
ERLIN1	*DGKQ*		
ERN1	*DGKZ*		
ERP29	*DHCR24*		
ERRFI1	*DHRS13*		
ESCO1	*DHRS3*		
ESPN	*DHX30*		
ESRRA	*DHX58*		
ETAA1	*DICER1*		
ETFB	*DIP2A*		
ETHE1	*DIRAS2*		
ETNK2	*DISC1*		
ETS1	*DIXDC1*		
EVI5L	*DKK2*		
EVPL	*DLG1*		
EXO1	*DLGAP2*		
EXOC6	*DLK1*		
EXOSC2	*DLST*		
EXT2	*DMPK*		
EXTL3	*DMRT2*		
F11R	*DMRTA1*		
F630043A04RIK	*DMXL1*		
F730047E07RIK	*DNA2*		
FA2H	*DNAI2*		
FAH	*DNAJA1*		
FAIM	*DNAJB11*		
FAM170A	*DNAJB12*		
FANCI	*DNAJC16*		
FANCM	*DNAJC18*		
FAP	*DNAJC3*		
FASTKD3	*DNAJC4*		
FAT4	*DNAJC5G*		
FBN1	*DNAJC7*		
FBP2	*DNASE1L2*		
FBXL12	*DNASE2*		
FBXL18	*DNHD1*		

FBXL2	*DNMT1*		
FBXO11	*DOCK2*		
FBXO2	*DOCK5*		
FBXO25	*DOCK6*		
FBXO30	*DOCK7*		
FBXO34	*DOCK8*		
FBXO6	*DOK2*		
FBXW4	*DOK4*		
FBXW5	*DOK6*		
FBXW8	*DOLPP1*		
FCER1A	*DOT1L*		
FCER2A	*DPF1*		
FCGR2B	*DPH3*		
FCNA	*DPH4*		
FDFT1	*DPM2*		
FECH	*DPM3*		
FERMT1	*DPP3*		
FGD4	*DPPA4*		
FGF12	*DR1*		
FGF16	*DRD5*		
FGF9	*DSCR10*		
FGGY	*DSCR3*		
FGL2	*DSCR4*		
FHDC1	*DSG2*		
FIGF	*DSG3*		
FILIP1	*DTWD1*		
FKBP4	*DUS1L*		
FLCN	*DUS2L*		
FLNA	*DUSP10*		
FLRT3	*DUSP13*		
FLT1	*DUSP2*		
FLVCR2	*DUSP28*		
FMO1	*DUSP8*		
FMO4	*DUT*		
FNBP4	*DVL2*		
FNDC4	*DYDC1*		
FNDC7	*DYX1C1*		
FNTA	*DZIP1L*		
FOLR2	*E2F1*		
FOLR4	*EAPP*		
FOXA2	*EBF2*		
FOXD1	*EBI3*		
FOXD2	*ECE2*		
FOXI2	*EDAR*		
FOXJ2	*EDARADD*		
FOXJ3	*EDG4*		

FOXM1	*EDNRA*		
FOXN1	*EED*		
FOXRED1	*EEF1A1*		
FPGT	*EEPD1*		
FREM1	*EFCAB4A*		
FREM2	*EFCAB4B*		
FRMD8	*EFCAB5*		
FRMPD1	*EFHD1*		
FRRS1	*EFTUD1*		
FSCN1	*EHBP1L1*		
FSD1	*EHD1*		
FTL1	*EHD4*		
FUK	*EIF2AK1*		
FURIN	*EIF2B2*		
FUT7	*EIF2B5*		
FUT8	*EIF2C4*		
FUT9	*EIF3A*		
FXC1	*EIF3B*		
FXYD3	*EIF3G*		
FXYD6	*ELAVL2*		
FZD3	*ELMOD1*		
From	*ELMOD2*		
G0S2	*ELOVL1*		
G3BP2	*ELOVL2*		
GABPA	*EMCN*		
GABPB2	*EME1*		
GABRA1	*EMG1*		
GABRA3	*EMID1*		
GABRB1	*EML5*		
GABRD	*EMP1*		
GABRG2	*EMR1*		
GABRG3	*EMX1*		
GADD45A	*EN2*		
GAK	*ENAM*		
GALNT1	*ENO1*		
GALNT2	*ENOPH1*		
GALNT3	*ENPP4*		
GALNT7	*ENPP5*		
GALNTL1	*ENTPD1*		
GALR1	*ENTPD6*		
GALR3	*EPAS1*		
GAMT	*EPB41L4B*		
GARS	*EPC1*		
GAS2	*EPC2*		
GBL	*EPHA1*		
GCAT	*EPHA7*		

GCDH	*EPHX2*		
GCK	*EPS15L1*		
GCLC	*ERAP1*		
GDF6	*ERBB4*		
GDF9	*ERCC2*		
GDPD2	*ERCC5*		
GFM2	*ERLIN1*		
GGA3	*ERMP1*		
GGCT	*ERRFI1*		
GGT1	*ESCO1*		
GIGYF2	*ESF1*		
GIMAP7	*ESRRA*		
GIN1	*ETF1*		
GIYD2	*ETFB*		
GJB3	*ETFDH*		
GJB5	*ETS1*		
GJC1	*ETV5*		
GJD4	*EVI5L*		
GLB1L2	*EVPL*		
GLCE	*EXDL2*		
GLDC	*EXOC3L2*		
GLIPR1L2	*EXOC4*		
GLTSCR1	*EXOSC1*		
GM128	*EXOSC10*		
GM1568	*F9*		
GM1673	*FA2H*		
GM1679	*FABP3*		
GM266	*FAH*		
GM561	*FAM101A*		
GM806	*FAM109A*		
GM813	*FAM109B*		
GM962	*FAM110A*		
GMEB1	*FAM123B*		
GMEB2	*FAM134C*		
GMFB	*FAM13A1*		
GMNN	*FAM19A2*		
GMPPB	*FAM20A*		
GMPR2	*FAM20B*		
GNA12	*FAM24A*		
GNAI2	*FAM26E*		
GNAI3	*FAM49A*		
GNAO1	*FAM57A*		
GNAT1	*FAM58A*		
GNAT2	*FAM63A*		
GNAZ	*FAM71F1*		
GNE	*FAM73B*		

GNG4	*FAM76A*		
GNPAT	*FAM81B*		
GNRH1	*FAM82C*		
GNS	*FAM83H*		
GOLGA1	*FAP*		
GOLGA4	*FARSA*		
GOSR2	*FAS*		
GP2	*FBLIM1*		
GPATCH3	*FBXL13*		
GPC5	*FBXL15*		
GPD2	*FBXL4*		
GPER	*FBXL8*		
GPI1	*FBXO10*		
GPM6A	*FBXO16*		
GPR126	*FBXO27*		
GPR137B	*FBXO39*		
GPR146	*FBXO41*		
GPR156	*FBXO42*		
GPR162	*FBXO7*		
GPR174	*FBXW5*		
GPR26	*FCER2*		
GPR4	*FCN1*		
GPR44	*FCN3*		
GPR45	*FCRL1*		
GPR56	*FCRL2*		
GPR64	*FDFT1*		
GPRC5A	*FECH*		
GPSM3	*FES*		
GPT2	*FETUB*		
GPX7	*FEV*		
GRB7	*FFAR2*		
GRCC10	*FGF17*		
GREB1	*FGF2*		
GREM1	*FGFR2*		
GRHPR	*FGFR3*		
GRID2IP	*FGGY*		
GRIT	*FGL1*		
GRK5	*FHL2*		
GRM3	*FKBP15*		
GSDMD	*FKBP1B*		
GSTA1	*FLRT2*		
GSTA2	*FLVCR2*		
GSTM5	*FMO1*		
GSTO1	*FMO5*		
GTF2A1	*FMOD*		
GTF2E1	*FMR1NB*		

GTF2E2	*FNBP1L*		
GTF2H3	*FNDC3A*		
GTF2H4	*FNDC4*		
GTF3C1	*FNTA*		
GYK	*FOXC1*		
GYS1	*FOXD1*		
GZF1	*FOXJ2*		
H2-EB1	*FOXK1*		
H2-M3	*FOXO1*		
H2-Q7	*FRG2C*		
H2-T22	*FRMD4B*		
H2AFY	*FRMD6*		
H3F3B	*FSCN1*		
HAGH	*FSHR*		
HAS2	*FSTL3*		
HAVCR2	*FTL*		
HBA-A1	*FUK*		
HBQ1	*FUNDC1*		
HCRT	*FURIN*		
HDAC10	*FUS*		
HDAC6	*FUT8*		
HDAC8	*FXC1*		
HDDC2	*From*		
HDGF	*G6PC*		
HDGFL1	*G6PC3*		
HDHD1A	*G6PD*		
HDLBP	*GABBR2*		
HEBP2	*GABPB2*		
HELZ	*GABRA3*		
HERPUD2	*GABRA6*		
HES1	*GABRG3*		
HEXB	*GABRQ*		
HEY1	*GABRR2*		
HGFAC	*GADD45A*		
HHAT	*GADD45G*		
HHIP	*GALM*		
HIGD1C	*GALNAC4S-6ST*		
HIP2	*GALNT1*		
HIPK3	*GALNT12*		
HIRA	*GALNT3*		
HIST2H2BE	*GALNT7*		
HLCS	*GART*		
HLX	*GAS2L1*		
HMG20A	*GAS8*		
HMG20B	*GATA2*		
HMOX1	*GATA5*		

HNRNPUL2	*GATA6*		
HOXA1	*GBA3*		
HOXC8	*GBL*		
HOXD12	*GCA*		
HOXD13	*GCET2*		
HPS4	*GCG*		
HPSE	*GCLM*		
HPSE2	*GCNT2*		
HRASLS	*GDE1*		
HRASLS5	*GDF11*		
HS2ST1	*GDI2*		
HS3ST1	*GFER*		
HSD11B1	*GFI1*		
HSD17B12	*GGT5*		
HSD17B7	*GHDC*		
HSD3B1	*GIGYF1*		
HSD3B2	*GIN1*		
HSD3B5	*GINS4*		
HSD3B6	*GJA5*		
HSD3B7	*GJB3*		
HSDL1	*GJB5*		
HSDL2	*GK*		
HSPA1B	*GKN2*		
HSPA4	*GLCCI1*		
HSPA5	*GLDC*		
HTRA1	*GLG1*		
HUS1	*GLI4*		
HVCN1	*GLIPR1L1*		
HYAL2	*GLIS2*		
HYAL5	*GLP1R*		
HYOU1	*GLRX*		
IBTK	*GLRX5*		
ICMT	*GLT8D3*		
IDH1	*GLUD2*		
IDI1	*GLULD1*		
IFI47	*GLYCTK*		
IFITM6	*GMFB*		
IFLTD1	*GMIP*		
IFNAR2	*GML*		
IFNG	*GNAI2*		
IFNGR1	*GNB4*		
IFNK	*GNE*		
IFRG15	*GNG2*		
IFT140	*GNG7*		
IGF2R	*GNPDA1*		
IGFALS	*GOLGA1*		

IGFBP4	*GOLGA8A*		
IHPK3	*GOLPH3L*		
IKBKE	*GOLT1A*		
IKZF3	*GP9*		
IKZF5	*GPATCH3*		
IL12RB1	*GPBAR1*		
IL17RA	*GPC6*		
IL17RD	*GPD1*		
IL19	*GPKOW*		
IL1A	*GPM6A*		
IL1RAP	*GPR103*		
IL21	*GPR108*		
IL21R	*GPR125*		
IL23R	*GPR132*		
IL25	*GPR137*		
IL28RA	*GPR137B*		
IL31RA	*GPR171*		
IL6RA	*GPR180*		
IL8RB	*GPR19*		
ILF3	*GPR20*		
ING1	*GPR26*		
ING3	*GPR61*		
ING5	*GPR63*		
INHBE	*GPR64*		
INPP5A	*GPR92*		
INPP5D	*GPR98*		
INPP5E	*GPS2*		
INVS	*GPSM3*		
IQCE	*GPT2*		
IQCG	*GPX7*		
IRF2BP1	*GRAMD1C*		
IRS3	*GRB7*		
IRS4	*GREM2*		
ISG20	*GRHL1*		
ISG20L2	*GRIA3*		
ISLR2	*GRIK5*		
ITCH	*GRIP1*		
ITFG1	*GRM3*		
ITGA11	*GRM5*		
ITGA2B	*GRM8*		
ITGA8	*GRWD1*		
ITGAX	*GSK3B*		
ITGB2	*GSTK1*		
ITGBL1	*GSTO1*		
ITK	*GTF2B*		
ITLN1	*GTF2H5*		

ITPKC	*GTF3A*		
ITPR2	*GTF3C1*		
ITSN1	*GTF3C3*		
ITSN2	*GTF3C4*		
JAG1	*GTPBP2*		
JDP2	*GTSF1L*		
JMJD2A	*GUCY1B3*		
JOSD1	*GUCY2C*		
JPH3	*GUF1*		
JRK	*GYLTL1B*		
JUB	*GYPB*		
JUP	*GZF1*		
KCNA1	*GZMA*		
KCNAB3	*GZMB*		
KCNE1L	*GZMM*		
KCNF1	*H2AFY2*		
KCNG4	*H3F3B*		
KCNIP4	*H6PD*		
KCNJ11	*HACE1*		
KCNJ8	*HACL1*		
KCNK3	*HADH*		
KCNMB2	*HAO1*		
KCNN3	*HAO2*		
KCNN4	*HAPLN2*		
KCNQ5	*HARS*		
KCNS2	*HBG2*		
KCNT1	*HBS1L*		
KCNV1	*HCCA2*		
KCTD13	*HCRT*		
KCTD15	*HCST*		
KCTD3	*HDAC1*		
KCTD5	*HDAC5*		
KDELR2	*HDAC8*		
KDELR3	*HDC*		
KDSR	*HEBP1*		
KIF13A	*HELZ*		
KIF17	*HEPACAM*		
KIF19A	*HERPUD2*		
KIF2C	*HES6*		
KIF5C	*HEY1*		
KLC1	*HEY2*		
KLC2	*HGS*		
KLC3	*HGSNAT*		
KLF11	*HIC1*		
KLF12	*HIF1AN*		
KLF9	*HIGD1A*		

KLHDC8A	*HIP1R*		
KLHDC8B	*HISPPD1*		
KLHL10	*HIST1H2AK*		
KLHL20	*HIST1H4D*		
KLHL24	*HIST2H2BE*		
KPNA3	*HLA-A*		
KPRP	*HLA-B*		
KRBA1	*HLA-C*		
KRR1	*HLA-DQA1*		
KRT12	*HLA-DQB1*		
KRT14	*HLX*		
KRT15	*HMBOX1*		
KRT17	*HMCN1*		
KRT32	*HMG2L1*		
KRT34	*HMGCS1*		
KRTAP16-7	*HMGN3*		
KRTAP26-1	*HMOX1*		
KRTDAP	*HNRNPA2B1*		
KTI12	*HNRPA3*		
KY	*HOMER1*		
L7RN6	*HOMER3*		
LACTB	*HOPX*		
LAGE3	*HORMAD1*		
LAMB2	*HOXA1*		
LAMC3	*HOXA13*		
LANCL3	*HOXA6*		
LBR	*HOXB2*		
LBX2	*HOXB4*		
LCE1B	*HOXC13*		
LCE1L	*HPS4*		
LCK	*HPS5*		
LCN12	*HPSE2*		
LCOR	*HRB*		
LCORL	*HS6ST2*		
LEF1	*HSD11B2*		
LEFTY1	*HSD17B6*		
LEFTY2	*HSD3B2*		
LENG1	*HSF2BP*		
LEPRE1	*HSPA1B*		
LGI1	*HSPB6*		
LGI3	*HSPBP1*		
LHCGR	*HTATIP*		
LHX3	*HTN1*		
LHX8	*HTN3*		
LIF	*HTR1F*		
LIG4	*HTR3A*		

LIMCH1	*HTR3B*		
LIMD1	*HTRA2*		
LIMK1	*HTT*		
LIN28	*HUS1*		
LINGO3	*ICAM3*		
LIPC	*ICOSLG*		
LIPE	*IDH3G*		
LIPF	*IFI35*		
LIPH	*IFITM2*		
LIPI	*IFNA21*		
LMAN2L	*IFT140*		
LMBR1	*IFT74*		
LMBR1L	*IGFBP7*		
LMNB2	*IGSF21*		
LMOD1	*IGSF5*		
LMTK2	*IHPK3*		
LNX2	*IKIP*		
LOXL2	*IKZF2*		
LOXL3	*IKZF3*		
LPAR3	*IKZF4*		
LPO	*IL12A*		
LPP	*IL12RB2*		
LRCH1	*IL15*		
LRCH3	*IL17RD*		
LRIG1	*IL17RE*		
LRP2BP	*IL1A*		
LRP4	*IL1F10*		
LRRC14	*IL1F9*		
LRRC15	*IL1RAPL2*		
LRRC23	*IL1RL1*		
LRRC36	*IL21R*		
LRRC52	*IL5RA*		
LRRC55	*IL8RB*		
LRRC56	*ILDR1*		
LRRFIP2	*IMMP2L*		
LRRK2	*IMPACT*		
LRRN2	*ING2*		
LSG1	*INHBB*		
LTB4R1	*INPP4A*		
LTF	*INPP5B*		
LUC7L	*INSC*		
LUZP1	*INSIG1*		
LYNX1	*INTS7*		
LYRM4	*INTS9*		
LYRM5	*INVS*		
LYSMD1	*IQCB1*		

LYSMD2	*IQUB*		
LYZL6	*IRAK1BP1*		
LZTS2	*IRAK3*		
MAB21L1	*IRF2BP1*		
MACROD2	*IRF5*		
MAF	*IRS2*		
MAF1	*IRX4*		
MAFB	*ITGA10*		
MAGI3	*ITGA11*		
MAK	*ITGA3*		
MAL	*ITGB1BP3*		
MAN1B1	*ITGB3*		
MAN1C1	*ITIH1*		
MAN2C1	*ITPR2*		
MAP1LC3A	*ITPR3*		
MAP2K4	*ITSN2*		
MAP3K12	*IVD*		
MAP3K14	*JAG1*		
MAP3K2	*JAKMIP2*		
MAP3K3	*JAM3*		
MAP3K7IP1	*JMJD1A*		
MAP3K7IP2	*JMJD2B*		
MAP3K7IP3	*KALRN*		
MAPK3	*KARS*		
MAPK4	*KATNAL1*		
MAPKAPK2	*KATNB1*		
MARCH4	*KCMF1*		
MARCKS	*KCNA1*		
MARE	*KCNA2*		
MARVELD2	*KCNB2*		
MAS1	*KCNC3*		
MASTL	*KCNE1L*		
MAT2A	*KCNF1*		
MBL1	*KCNG1*		
MBOAT1	*KCNH1*		
MC3R	*KCNH4*		
MCART6	*KCNH8*		
MCM7	*KCNIP4*		
MCOLN2	*KCNJ11*		
MCPT8	*KCNK10*		
MED1	*KCNK3*		
MED20	*KCNK9*		
MED22	*KCNMA1*		
MED6	*KCNN3*		
MED8	*KCNQ5*		
MED9	*KCNS1*		

MEIG1	*KCTD13*		
MEIS2	*KCTD5*		
MESDC2	*KDELR2*		
METRN	*KDELR3*		
METTL3	*KIAA0133*		
METTL9	*KIAA0174*		
MFAP3	*KIAA0241*		
MFAP4	*KIAA0247*		
MFF	*KIAA0284*		
MFSD1	*KIAA0317*		
MFSD2	*KIAA0355*		
MFSD8	*KIAA0556*		
MGAT4A	*KIAA0586*		
MGAT5B	*KIAA0652*		
MGST2	*KIAA0664*		
MGST3	*KIAA0754*		
MICAL1	*KIAA1147*		
MICAL2	*KIAA1274*		
MIR16	*KIAA1279*		
MITD1	*KIAA1324L*		
MIXL1	*KIAA1383*		
MKI67IP	*KIAA1409*		
MKL2	*KIAA1429*		
MKRN1	*KIAA1430*		
MKRN2	*KIAA1530*		
MKRN3	*KIAA1576*		
MLXIP	*KIAA1600*		
MLYCD	*KIAA1755*		
MMAB	*KIAA1797*		
MMD	*KIAA1804*		
MMP1B	*KIF16B*		
MMP9	*KIF18A*		
MMS19	*KIF1B*		
MN1	*KIF23*		
MND1	*KIF5A*		
MOBKL1A	*KIF5C*		
MOBKL2B	*KISS1*		
MON1B	*KIT*		
MON2	*KLC1*		
MORN3	*KLC3*		
MORN5	*KLC4*		
MOV10L1	*KLF15*		
MPDU1	*KLF4*		
MPP1	*KLF5*		
MPP2	*KLF6*		
MPRIP	*KLF8*		

MPZL2	*KLHDC3*		
MREG	*KLHDC8B*		
MRGPRA4	*KLHL10*		
MRM1	*KLHL17*		
MRP63	*KLK13*		
MRPL17	*KLKBL4*		
MRPL18	*KLRK1*		
MRPL21	*KRAS*		
MRPL24	*KRR1*		
MRPL36	*KRT16*		
MRPS16	*KRT20*		
MRPS17	*KRT33B*		
MRPS2	*KRT34*		
MRPS6	*KRT6C*		
MRPS7	*KRT73*		
MRPS9	*KRT75*		
MRRF	*KRT76*		
MTA2	*KRT80*		
MTAP	*KRT85*		
MTCH2	*KRT86*		
MTDH	*KRTAP1-3*		
MTFR1	*KRTAP1-5*		
MTHFD1	*KRTAP11-1*		
MTHFD1L	*KRTAP4-4*		
MTMR4	*KRTAP4-5*		
MTRF1	*KRTAP9-3*		
MTSS1	*KRTAP9-4*		
MTTP	*KSR1*		
MTUS1	*KYNU*		
MTVR2	*LAIR2*		
MTX1	*LALBA*		
MUC1	*LAMB2*		
MUC15	*LAMC3*		
MUC5AC	*LASS3*		
MYBL2	*LASS6*		
MYCT1	*LBX1*		
MYH14	*LCK*		
MYH3	*LCN2*		
MYH8	*LCP2*		
MYL1	*LCT*		
MYLK	*LDB1*		
MYO10	*LDB3*		
MYO1D	*LDOC1L*		
MYO1F	*LELP1*		
MYO5B	*LEMD1*		
MYOM1	*LEPREL2*		

MYRIP	*LEPROTL1*		
MYST1	*LFNG*		
MZF1	*LGI1*		
N28178	*LGI3*		
NAB2	*LHCGR*		
NADK	*LHX5*		
NAGPA	*LHX8*		
NANP	*LIG3*		
NAP1L3	*LIMCH1*		
NAPB	*LIMS1*		
NAT1	*LIN54*		
NAT14	*LIPE*		
NAV1	*LIPI*		
NCAM2	*LITAF*		
NCAPG2	*LMBR1L*		
NCBP1	*LMLN*		
NCK1	*LMTK3*		
NCKAP1L	*LOC642587*		
NCKIPSD	*LOC643923*		
NCLN	*LOX*		
NCOA2	*LOXL4*		
NCOR2	*LPCAT1*		
NDFIP1	*LPCAT2*		
NDRG4	*LPHN3*		
NDUFA4	*LPIN2*		
NDUFB5	*LPIN3*		
NDUFS5	*LPO*		
NEBL	*LRDD*		
NEDD1	*LRFN2*		
NEFL	*LRFN4*		
NEK11	*LRIG2*		
NEK9	*LRP1B*		
NETO1	*LRP8*		
NETO2	*LRRC16B*		
NEURL	*LRRC17*		
NEUROD1	*LRRC20*		
NEUROD2	*LRRC27*		
NEUROG3	*LRRC29*		
NFASC	*LRRC41*		
NFATC3	*LRRC52*		
NFE2L2	*LRRC8D*		
NFKBIL2	*LRRN2*		
NFS1	*LRRTM1*		
NFYA	*LSM6*		
NG23	*LSS*		
NGDN	*LYN*		

NGF	*LYPD1*		
NGP	*LYPLA3*		
NHEDC2	*LYSMD2*		
NIPSNAP3A	*LYST*		
NKG7	*LZTFL1*		
NKX6-1	*MAF1*		
NKX6-2	*MAFB*		
NLGN1	*MAGEA1*		
NLRP1B	*MAGEA4*		
NLRX1	*MAGEA8*		
NME3	*MAGEE1*		
NMI	*MAGEH1*		
NODAL	*MAGI3*		
NOL4	*MAGIX*		
NOL5A	*MALL*		
NOLA2	*MAML1*		
NOLC1	*MAMLD1*		
NONO	*MAN1A2*		
NOXA1	*MAN1B1*		
NPAS4	*MAOA*		
NPEPL1	*MAP1A*		
NPFFR2	*MAP2K2*		
NPLOC4	*MAP3K1*		
NPNT	*MAP3K12*		
NPPA	*MAP3K14*		
NPPC	*MAP3K4*		
NPR1	*MAP3K6*		
NPR2	*MAP3K7IP3*		
NPSR1	*MAPKAPK2*		
NR1H5	*MAPRE3*		
NR2C2AP	*MAPT*		
NRARP	*MARCH4*		
NRBP1	*MARCH9*		
NRD1	*MARCO*		
NRN1L	*MARK1*		
NRSN2	*MAST4*		
NSG1	*MAT2A*		
NT5DC1	*MAX*		
NT5DC2	*MBD1*		
NT5E	*MBD3*		
NTHL1	*MBD6*		
NTN4	*MBOAT1*		
NUDT10	*MBOAT2*		
NUDT2	*MCCC1*		
NUDT3	*MCF2L*		
NUDT4	*MCM3AP*		

NUDT6	*MCTS1*		
NUMA1	*MDH2*		
NUPL2	*MDM2*		
NUS1	*MECR*		
NUT	*MED11*		
NWD1	*MED12*		
NXNL1	*MED15*		
NXPH1	*MED27*		
NXPH2	*MED8*		
OAS1C	*MED9*		
OAS1D	*MEIS3*		
OAS1E	*METAP2*		
OAS1H	*METTL1*		
OASL1	*METTL9*		
OASL2	*MEX3B*		
OBFC2A	*MEX3C*		
OBOX3	*MFAP4*		
ODZ3	*MFN1*		
OLFM2	*MFRP*		
OLFM3	*MGAT5*		
OLFML2B	*MGAT5B*		
OLFR43	*MGC33894*		
OLFR59	*MGC42105*		
OLFR70	*MIB1*		
OLR1	*MICAL1*		
OMG	*MICAL3*		
OPA3	*MICALCL*		
OPALIN	*MID2*		
OPCML	*MINPP1*		
OPRL1	*MIOX*		
OPRS1	*MIP*		
ORC3L	*MIZF*		
ORC5L	*MKL1*		
ORMDL2	*MKRN3*		
ORMDL3	*MKS1*		
OSBPL1A	*MLF1IP*		
OSGEP	*MLH1*		
OSGEPL1	*MLKL*		
OSGIN2	*MLSTD1*		
OSTA	*MLXIP*		
OTOS	*MMD*		
OTT	*MME*		
OTUB1	*MMP1*		
OTUB2	*MMP10*		
OXCT2A	*MMP15*		
OXSM	*MMP21*		

OXT	*MMP24*		
P2RX3	*MMP28*		
P2RX6	*MMP3*		
P2RY2	*MMP8*		
P2RY6	*MNT*		
P4HA1	*MOAP1*		
P4HA2	*MOBKL2C*		
P4HA3	*MOCS1*		
PACS1	*MON1A*		
PACSIN2	*MON1B*		
PACSIN3	*MPDU1*		
PADI4	*MPI*		
PAH	*MPP1*		
PAIP1	*MPP4*		
PAK7	*MPST*		
PAPPA	*MREG*		
PAQR4	*MRGPRX3*		
PAQR9	*MRPL18*		
PARC	*MRPL28*		
PARL	*MRPL37*		
PARP14	*MRPL4*		
PARP2	*MRPL40*		
PARVB	*MRPS2*		
PAWR	*MRPS21*		
PAX3	*MS4A4A*		
PBK	*MSI1*		
PBLD	*MTA3*		
PBP2	*MTBP*		
PCDH17	*MTDH*		
PCDH20	*MTFR1*		
PCDHB13	*MTG1*		
PCDHB19	*MTHFD2L*		
PCDHB21	*MTHFR*		
PCDHB22	*MTMR1*		
PCSK4	*MTMR10*		
PCSK9	*MTMR11*		
PDC	*MTMR14*		
PDCD6	*MTMR2*		
PDE10A	*MTTP*		
PDE1A	*MUC20*		
PDE1B	*MUCL1*		
PDE4A	*MUM1*		
PDE7A	*MVD*		
PDE8A	*MXD3*		
PDGFB	*MYBL2*		
PDK1	*MYCN*		

PDK4	*MYD88*		
PDLIM3	*MYH3*		
PDLIM4	*MYH6*		
PDLIM5	*MYO18B*		
PDLIM7	*MYO1G*		
PDRG1	*MYOC*		
PDSS1	*MYOM3*		
PDXK	*MYOZ2*		
PDZD4	*MYOZ3*		
PEA15A	*N4BP3*		
PEBP1	*NAB2*		
PEF1	*NACA*		
PELI3	*NACA2*		
PEO1	*NADK*		
PEPD	*NAGPA*		
PER2	*NAPG*		
PEX11A	*NAT14*		
PEX6	*NAT6*		
PFDN5	*NAT8*		
PFKL	*NAT8B*		
PGAM2	*NAT9*		
PGCP	*NBEA*		
PGD	*NBL1*		
PGM1	*NCLN*		
PGM2	*NCOA2*		
PGM2L1	*NCOR2*		
PHACTR2	*NDUFA12*		
PHC3	*NDUFA4*		
PHF19	*NDUFB10*		
PHKA1	*NDUFB8*		
PHLDB2	*NDUFS4*		
PHOX2B	*NEDD1*		
PI16	*NEFL*		
PI4K2A	*NEFM*		
PI4KA	*NEK10*		
PIGA	*NES*		
PIGH	*NEURL*		
PIGL	*NEUROD1*		
PIGM	*NEUROD2*		
PIK3R3	*NEUROD4*		
PIK3R4	*NEUROG3*		
PIK3R5	*NEXN*		
PIP	*NFASC*		
PIP5K1B	*NFATC3*		
PIP5K1C	*NFATC4*		
PITPNM2	*NFE2L3*		

PIWIL1	*NFKBIA*		
PKD1	*NFKBIL1*		
PKMYT1	*NFRKB*		
PKNOX2	*NGEF*		
PLA2G2F	*NHLRC3*		
PLA2G5	*NIP30*		
PLA2G6	*NIPBL*		
PLAC1L	*NISCH*		
PLAG1	*NKX3-1*		
PLCB1	*NKX3-2*		
PLCD1	*NLRC5*		
PLCH2	*NLRP14*		
PLCXD1	*NLRP3*		
PLD3	*NLRX1*		
PLEC1	*NME4*		
PLEKHA3	*NMI*		
PLEKHA6	*NMNAT2*		
PLEKHB2	*NMNAT3*		
PLEKHG1	*NMUR2*		
PLK4	*NOL1*		
PLRG1	*NOL4*		
PLXNB1	*NOPE*		
PMF1	*NOS1*		
PML	*NOS2A*		
PMM1	*NOS3*		
PMPCB	*NOX1*		
PNKD	*NOX4*		
PNMA3	*NPAS4*		
PNO1	*NPEPL1*		
PNP1	*NPHP4*		
PNPLA2	*NPR2*		
PNPLA6	*NPTX2*		
POFUT1	*NPY2R*		
POFUT2	*NR1I3*		
POLL	*NR2C2*		
POLM	*NR2C2AP*		
POLQ	*NR2F2*		
POLR2C	*NR4A1*		
POLR2I	*NRBP1*		
POLR3A	*NRIP3*		
POLR3E	*NSD1*		
POLR3H	*NSDHL*		
POLR3K	*NSF*		
POMC	*NSUN6*		
POMT2	*NT5C3*		
POU4F2	*NT5DC2*		

PPA1	*NT5E*		
PPA2	*NUDT14*		
PPAP2A	*NUDT15*		
PPAP2B	*NUMA1*		
PPAP2C	*NUP133*		
PPAPDC1A	*NUP188*		
PPARD	*NUP35*		
PPARGC1B	*NXF1*		
PPCS	*NXF3*		
PPID	*NXPH2*		
PPIH	*OAS3*		
PPM1D	*ODF1*		
PPM1E	*ODZ1*		
PPM1L	*OIP5*		
PPP1R12A	*OLFML1*		
PPP1R14D	*OLFML3*		
PPP1R15B	*OMG*		
PPP1R3D	*OOEP*		
PPP2R1A	*OPLAH*		
PPP2R2C	*OPN5*		
PPP2R5A	*OPRM1*		
PPP2R5C	*OPRS1*		
PPP3CA	*OR2H1*		
PPP4R2	*ORC3L*		
PPP5C	*ORMDL3*		
PPP6C	*OS9*		
PPT1	*OSBPL11*		
PPWD1	*OSBPL5*		
PRDX6-RS1	*OSGEP*		
PREPL	*OSGEPL1*		
PRICKLE1	*OSGIN2*		
PRICKLE4	*OSR1*		
PRKAG2	*OSTM1*		
PRKAR1A	*OTC*		
PRKAR2A	*OTUD6B*		
PRKCZ	*OVOL2*		
PRKG1	*OXR1*		
PRKRA	*P2RY11*		
PRKRIP1	*P4HA2*		
PRL2C2	*PAAF1*		
PRL2C4	*PABPC1L2B*		
PROM2	*PABPC4L*		
PROX1	*PACS1*		
PROZ	*PAF1*		
PRPF4	*PAFAH1B3*		
PRPF40B	*PAFAH2*		

PRR11	*PAK3*		
PRR16	*PAPD4*		
PRRXL1	*PAPD5*		
PRSS23	*PAQR3*		
PRTG	*PARP15*		
PSCD3	*PARP16*		
PSD2	*PARP3*		
PSENEN	*PARVA*		
PSG21	*PAWR*		
PSG22	*PAXIP1*		
PSG27	*PBOV1*		
PSG29	*PC*		
PSKH1	*PCBD2*		
PSMA3	*PCDH18*		
PSMA7	*PCGF5*		
PSMC1	*PCMTD2*		
PSMC6	*PCTK1*		
PSMD10	*PCYT2*		
PSMD11	*PDAP1*		
PSMD13	*PDC*		
PTCD2	*PDCD2L*		
PTCD3	*PDCD6*		
PTGDS	*PDE10A*		
PTGER1	*PDE1B*		
PTGER2	*PDE2A*		
PTGER4	*PDE4A*		
PTGFR	*PDGFC*		
PTGFRN	*PDLIM3*		
PTGIS	*PDP2*		
PTH	*PDS5A*		
PTHLH	*PDSS1*		
PTMS	*PDXDC1*		
PTPN1	*PDZD4*		
PTPN13	*PEBP4*		
PTPN14	*PERLD1*		
PTPN18	*PES1*		
PTPN2	*PEX11B*		
PTPN22	*PEX12*		
PTPN23	*PEX26*		
PTPN4	*PFDN6*		
PTPN5	*PFKFB4*		
PTPRF	*PFKM*		
PTPRM	*PFTK1*		
PTPRT	*PGAM2*		
PTRF	*PGBD2*		
PUM2	*PGD*		

PURB	*PGLS*		
PUS3	*PGLYRP1*		
PWP2	*PGM1*		
PYCRL	*PGM2*		
PYGO2	*PGM3*		
PYROXD1	*PHACTR3*		
QRFP	*PHACTR4*		
R3HDM1	*PHEX*		
RAB10	*PHF15*		
RAB11FIP4	*PHF2*		
RAB11FIP5	*PHF21A*		
RAB13	*PHF21B*		
RAB15	*PHF23*		
RAB27A	*PHF5A*		
RAB32	*PHGDH*		
RAB34	*PHIP*		
RAB40C	*PHKG1*		
RAB4B	*PHKG2*		
RAB6B	*PHOX2B*		
RAB7L1	*PHTF1*		
RAB8A	*PHTF2*		
RABEP1	*PIAS2*		
RABEPK	*PICK1*		
RABIF	*PIGK*		
RABL4	*PIGM*		
RABL5	*PIGN*		
RAC1	*PIGS*		
RAD50	*PIGY*		
RAD52	*PIK3AP1*		
RAET1B	*PIK3C2B*		
RAET1C	*PIK3CD*		
RALB	*PIK3R2*		
RAPGEF1	*PIK3R6*		
RAPGEF3	*PIM3*		
RAPGEF6	*PIN4*		
RARRES2	*PIP*		
RASD1	*PIP5K1A*		
RASSF8	*PIP5K1B*		
RASSF9	*PITPNB*		
RAVER2	*PITPNM2*		
RBBP5	*PKD2*		
RBM13	*PLA2G2F*		
RBM15	*PLA2G4F*		
RBM19	*PLAA*		
RBM44	*PLAG1*		
RBM6	*PLCD1*		

RBMS2	*PLCL1*	
RCC2	*PLCZ1*	
RCN1	*PLEKHA6*	
RCN3	*PLEKHA9*	
RCOR2	*PLEKHF2*	
RDH11	*PLEKHH1*	
RDH12	*PLEKHJ1*	
RDH9	*PLEKHM1*	
REEP3	*PLEKHO2*	
REG3D	*PLTP*	
REL	*PLXNA4*	
RELL1	*PLXNB1*	
REV1	*PMF1*	
REV3L	*PMPCB*	
RFC2	*PNLIPRP1*	
RFESD	*PNMA1*	
RFTN2	*PNO1*	
RFWD3	*PNOC*	
RFXDC1	*PNPLA1*	
RG9MTD3	*PNPLA2*	
RGMA	*PNPLA6*	
RGS10	*PNPT1*	
RGS16	*PNRC2*	
RGS19	*PODN*	
RGS2	*PODXL2*	
RGS3	*POLDIP2*	
RGS7BP	*POLE4*	
RGS8	*POLQ*	
RGS9BP	*POLR2A*	
RHBDL3	*POLR3D*	
RHEBL1	*POLS*	
RHOBTB1	*POMC*	
RHOD	*POP4*	
RHOV	*POPDC3*	
RHOX10	*POSTN*	
RHOX11	*POU2F3*	
RHOX12	*POU4F2*	
RHOX6	*POU6F1*	
RIC3	*PPAP2A*	
RIMBP2	*PPAPDC1B*	
RIOK3	*PPAPDC2*	
RMND5B	*PPARG*	
RNASEH1	*PPARGC1B*	
RNASEH2B	*PPAT*	
RNF10	*PPBP*	
RNF12	*PPFIA4*	

RNF135	*PPFIBP1*	
RNF138	*PPHLN1*	
RNF151	*PPID*	
RNF17	*PPIF*	
RNF219	*PPIH*	
RNF220	*PPIL2*	
RNF25	*PPIL3*	
RNF40	*PPIL4*	
RNF5	*PPL*	
RNF8	*PPM1E*	
RNGTT	*PPP1R14C*	
RNPS1	*PPP1R2*	
RP9	*PPP1R3C*	
RPA2	*PPP1R7*	
RPGR	*PPP2CA*	
RPL13	*PPP2R1A*	
RPL18A	*PPP2R1B*	
RPL28	*PPP2R2A*	
RPL36A	*PPP2R3A*	
RPL37A	*PPP5C*	
RPL39	*PPT1*	
RPL6	*PPTC7*	
RPL7L1	*PQLC3*	
RPN1	*PRDM1*	
RPN2	*PRDM13*	
RPP25	*PREB*	
RPP38	*PRELID2*	
RPP40	*PRICKLE1*	
RPS19BP1	*PRKAG2*	
RPS24	*PRKCE*	
RPS5	*PRKCZ*	
RPS6KA5	*PRKD3*	
RPS6KB1	*PRKG1*	
RPS6KB2	*PRKG2*	
RPS6KC1	*PRMT2*	
RPS6KL1	*PRMT6*	
RRAD	*PRMT7*	
RRAS	*PRNP*	
RS1	*PROL1*	
RSAD1	*PROZ*	
RSF1	*PRPF38A*	
RSPO2	*PRPH2*	
RSPRY1	*PRR16*	
RTCD1	*PRR18*	
RTN4	*PRR6*	
RTN4R	*PRRG3*	

RUFY1	*PRRT2*
RUNX1	*PRSS21*
RUNX1T1	*PRSS7*
RUNX2	*PRSS8*
RUSC1	*PRTG*
RWDD3	*PSAP*
RWDD4A	*PSCA*
RYR1	*PSCDBP*
S100A16	*PSD2*
S100A3	*PSD4*
S100A5	*PSKH1*
SALL3	*PSMB3*
SAMD10	*PSMB6*
SAMM50	*PSMB7*
SAP30	*PSMB8*
SAP30BP	*PSMC3*
SAR1A	*PSMC5*
SARDH	*PSMD1*
SARM1	*PSMD13*
SARS	*PSMD14*
SART1	*PSMD7*
SATB2	*PSORS1C1*
SBF1	*PSRC1*
SBK1	*PTAR1*
SCAMP1	*PTBP1*
SCAMP2	*PTCD3*
SCARA5	*PTCHD2*
SCARB1	*PTGER3*
SCCPDH	*PTGS2*
SCD2	*PTH*
SCEL	*PTP4A3*
SCG3	*PTPLB*
SCGB1A1	*PTPMT1*
SCGB3A1	*PTPN13*
SCGN	*PTPN18*
SCN1A	*PTPN2*
SCN3B	*PTPRA*
SCN4B	*PTPRK*
SCNN1G	*PTRH1*
SDAD1	*PUF60*
SDCBP2	*PUS10*
SDF2	*PVALB*
SDPR	*PXDN*
SDS	*PXDNL*
SEC14L4	*PXK*
SEC22C	*PXMP2*

SEC23B	*PXMP3*		
SECISBP2	*PYGO2*		
SEMA3A	*QARS*		
SEMA6D	*QPCTL*		
SEPP1	*QRICH1*		
SEPX1	*R3HDM1*		
SERBP1	*RAB11FIP3*		
SERINC1	*RAB11FIP5*		
SERINC4	*RAB15*		
SERP1	*RAB21*		
SERPINA3C	*RAB24*		
SERPINA3F	*RAB25*		
SERPINB12	*RAB26*		
SERPINB13	*RAB27B*		
SERPINB3A	*RAB32*		
SERPINB8	*RAB34*		
SERPINB9C	*RAB35*		
SERPINC1	*RAB3IP*		
SERPINF1	*RAB4B*		
SERPINF2	*RAB6A*		
SERPINI1	*RAB7L1*		
SERTAD2	*RABEPK*		
SESN1	*RABL4*		
SETD7	*RACGAP1*		
SF3A1	*RAD51*		
SF3B2	*RAD51L1*		
SF3B4	*RAD9A*		
SF3B5	*RAD9B*		
SFRS11	*RAG2*		
SFRS17B	*RAGE*		
SFXN2	*RAMP1*		
SFXN4	*RAMP3*		
SGCG	*RANBP2*		
SGIP1	*RANGAP1*		
SGPP1	*RAPGEF4*		
SGTB	*RAPGEF5*		
SH2B3	*RAPGEFL1*		
SH2D2A	*RARRES1*		
SH3BGR	*RASA1*		
SH3BP2	*RASAL1*		
SH3BP5	*RASAL2*		
SH3PXD2B	*RASGRF1*		
SH3RF2	*RASGRP3*		
SH3TC2	*RASIP1*		
SH3YL1	*RASL10B*		
SHISA3	*RASSF2*		

SHKBP1	*RASSF4*		
SIAH1A	*RASSF5*		
SIAH1B	*RAVER2*		
SIGIRR	*RBM15*		
SIL1	*RBM28*		
SIN3A	*RBM35B*		
SIRT1	*RBM4B*		
SIRT3	*RBM5*		
SIX5	*RBM6*		
SKIV2L2	*RBM8A*		
SKP1A	*RBMS2*		
SLC10A5	*RCAN2*		
SLC16A2	*RCL1*		
SLC16A4	*RCN3*		
SLC17A5	*RCOR2*		
SLC17A6	*RDH12*		
SLC19A2	*RDH16*		
SLC1A2	*RECQL4*		
SLC1A5	*REEP3*		
SLC22A18	*REEP4*		
SLC22A2	*REEP5*		
SLC23A2	*REG3A*		
SLC24A1	*RELA*		
SLC24A6	*RELL1*		
SLC25A10	*RELL2*		
SLC25A14	*REXO4*		
SLC25A16	*RFT1*		
SLC25A23	*RFTN2*		
SLC25A25	*RFX2*		
SLC25A38	*RFX5*		
SLC25A46	*RFXAP*		
SLC25A5	*RFXDC1*		
SLC26A4	*RGL4*		
SLC26A5	*RGMA*		
SLC28A3	*RGS10*		
SLC29A1	*RGS22*		
SLC29A4	*RGS4*		
SLC2A1	*RGS7BP*		
SLC2A3	*RHBDD1*		
SLC2A6	*RHBG*		
SLC34A2	*RHD*		
SLC35A5	*RHEBL1*		
SLC35B1	*RHOB*		
SLC35B2	*RHOBTB1*		
SLC35B3	*RHOC*		
SLC35B4	*RHOD*		

SLC35C1	*RHOQ*		
SLC35D1	*RHOT2*		
SLC36A1	*RICTOR*		
SLC37A1	*RILPL2*		
SLC37A3	*RIMS1*		
SLC38A11	*RIOK2*		
SLC38A7	*RIPK1*		
SLC39A11	*RIPK4*		
SLC39A5	*RNASE6*		
SLC3A1	*RNASE7*		
SLC41A2	*RNASEK*		
SLC41A3	*RNASET2*		
SLC43A2	*RND3*		
SLC43A3	*RNF13*		
SLC46A3	*RNF135*		
SLC47A2	*RNF138*		
SLC4A10	*RNF139*		
SLC4A9	*RNF217*		
SLC5A11	*RNF219*		
SLC6A13	*RNF25*		
SLC6A17	*RNF41*		
SLC6A20A	*RNGTT*		
SLC6A4	*RNUXA*		
SLC6A8	*ROGDI*		
SLC7A10	*ROM1*		
SLC7A13	*ROR2*		
SLC7A4	*RORA*		
SLC7A7	*RORC*		
SLC9A2	*RP11-529I10.4*		
SLCO2A1	*RPA2*		
SLCO4A1	*RPAP1*		
SLMO1	*RPL10L*		
SMAD2	*RPL11*		
SMARCAL1	*RPL27A*		
SMARCD1	*RPL37A*		
SMC6	*RPP25*		
SMG7	*RPP40*		
SMOX	*RPRML*		
SMPD1	*RPS20*		
SMPDL3B	*RPS3*		
SMS	*RPS6KA5*		
SMU1	*RPS6KA6*		
SMYD1	*RPS6KB2*		
SNAI1	*RPTN*		
SNAPIN	*RPUSD1*		
SNCG	*RRAD*		

SNF1LK	*RRAGA*		
SNF1LK2	*RRAGD*		
SNN	*RRAS*		
SNRK	*RSBN1L*		
SNRP70	*RSF1*		
SNTB1	*RSPO1*		
SNX15	*RSPO2*		
SNX19	*RSPO4*		
SNX2	*RTF1*		
SNX25	*RTN4R*		
SNX29	*RTN4RL1*		
SNX30	*RTTN*		
SNX33	*RUFY2*		
SNX4	*RUNX1T1*		
SNX5	*RXFP2*		
SOCS1	*RXRB*		
SOCS2	*S100A14*		
SOCS3	*S100A3*		
SON	*S100A5*		
SORCS2	*S100A8*		
SOS1	*S100P*		
SOSTDC1	*SACS*		
SOX11	*SALL4*		
SOX8	*SAP30BP*		
SP4	*SAP30L*		
SPAG6	*SAPS3*		
SPAG7	*SART3*		
SPARCL1	*SAT1*		
SPATA16	*SATB2*		
SPATA2	*SBF1*		
SPCS2	*SCAP*		
SPEER4B	*SCEL*		
SPEER4D	*SCGB1D2*		
SPEER4F	*SCN1A*		
SPEF1	*SCN2A*		
SPEN	*SCN2B*		
SPHK2	*SCN3A*		
SPIC	*SCN4A*		
SPINK2	*SCNN1A*		
SPINT1	*SCNN1D*		
SPINT2	*SCNN1G*		
SPNB3	*SCRT1*		
SPOCK3	*SDC2*		
SPON1	*SDCBP2*		
SPP2	*SDHD*		
SPRR2F	*SEC22A*		

SPRR2K	*SEC22C*		
SPTLC1	*SEC23A*		
SPTLC2	*SEC23B*		
SRFBP1	*SEC61A1*		
SRP68	*SEC61G*		
SRP72	*SEH1L*		
SRPX2	*SELENBP1*		
SRRP	*SELS*		
SSBP2	*SEMA3A*		
SSBP4	*SEMA3B*		
SSR1	*SEMA5A*		
SSR4	*SEMA6D*		
SSTR1	*SEMA7A*		
SSTR4	*SENP3*		
SSTR5	*SENP6*		
ST3GAL3	*SEPN1*		
ST3GAL5	*SEPSECS*		
ST6GAL1	*SERAC1*		
ST6GAL2	*SERP1*		
ST6GALNAC5	*SERPINA1*		
STAC	*SERPINA3*		
STAG3	*SERPINB1*		
STARD3NL	*SERPINB2*		
STAT3	*SERPINB8*		
STC1	*SERPINE1*		
STK17B	*SERPINF2*		
STK19	*SERPING1*		
STK38L	*SESN1*		
STOX2	*SESN2*		
STRN3	*SETD1A*		
STT3B	*SETD6*		
STUB1	*SETD7*		
STX4A	*SEZ6L2*		
STX5A	*SF3A2*		
SUCLG1	*SF3B3*		
SULF1	*SF4*		
SULT5A1	*SFN*		
SUNC1	*SFRS10*		
SUOX	*SFRS11*		
SUPT3H	*SFRS16*		
SURF4	*SFRS2IP*		
SVA	*SFT2D2*		
SYNGR3	*SFXN2*		
SYT1	*SGCA*		
SYT15	*SGCG*		
SYT4	*SGEF*		

SYTL1	*SGSM2*		
SYTL4	*SH2D2A*		
SYVN1	*SH3BGRL*		
T	*SH3BGRL2*		
TAC1	*SH3BP5*		
TACC2	*SH3GL1*		
TADA3L	*SH3MD4*		
TAF1C	*SH3PXD2A*		
TAF8	*SH3TC1*		
TAOK1	*SHBG*		
TAS1R2	*SHC4*		
TASP1	*SHD*		
TATDN1	*SHF*		
TBC1D12	*SHMT2*		
TBC1D13	*SHROOM2*		
TBC1D19	*SIAH1*		
TBC1D20	*SIDT1*		
TBCC	*SIGLEC8*		
TBPL1	*SIM2*		
TBPL2	*SIN3A*		
TBRG4	*SIRPB1*		
TBX10	*SIX4*		
TBXA2R	*SKAP2*		
TBXAS1	*SKIL*		
TCF25	*SKIV2L2*		
TCF7	*SLC10A1*		
TCFCP2	*SLC11A2*		
TCFCP2L1	*SLC12A5*		
TCFEB	*SLC12A7*		
TCFEC	*SLC13A2*		
TCN2	*SLC13A5*		
TCP1	*SLC15A4*		
TCP10	*SLC17A1*		
TCP11L1	*SLC1A5*		
TCP11L2	*SLC20A2*		
TCTA	*SLC22A1*		
TDGF1	*SLC22A11*		
TDH	*SLC22A18*		
TDRD7	*SLC22A5*		
TECTA	*SLC22A8*		
TEDDM1	*SLC25A10*		
TEP1	*SLC25A12*		
TERF1	*SLC25A15*		
TESP2	*SLC25A24*		
TFDP2	*SLC25A30*		
TFF3	*SLC25A40*		

TFRC	*SLC25A43*		
TG	*SLC25A45*		
TGDS	*SLC25A5*		
TGFBRAP1	*SLC26A11*		
TGIF2	*SLC26A9*		
THAP1	*SLC29A1*		
THAP2	*SLC2A1*		
THBD	*SLC2A4RG*		
THRB	*SLC30A4*		
THUMPD1	*SLC30A7*		
THY1	*SLC30A8*		
TICAM2	*SLC35A4*		
TIMD2	*SLC35A5*		
TIPARP	*SLC35B3*		
TIRAP	*SLC35D2*		
TJAP1	*SLC35D3*		
TK2	*SLC36A1*		
TKTL1	*SLC37A1*		
TLN1	*SLC37A3*		
TM2D2	*SLC38A7*		
TM2D3	*SLC38A9*		
TM4SF1	*SLC39A5*		
TM7SF3	*SLC39A9*		
TM9SF3	*SLC41A2*		
TMC7	*SLC41A3*		
TMCO1	*SLC43A2*		
TMCO3	*SLC45A3*		
TMCO4	*SLC46A3*		
TMED3	*SLC4A11*		
TMEFF1	*SLC5A10*		
TMEM104	*SLC5A11*		
TMEM121	*SLC6A14*		
TMEM128	*SLC6A3*		
TMEM132A	*SLC6A4*		
TMEM135	*SLC6A8*		
TMEM144	*SLC7A10*		
TMEM154	*SLC7A9*		
TMEM161A	*SLC8A1*		
TMEM162	*SLC9A6*		
TMEM177	*SLC9A8*		
TMEM208	*SLCO3A1*		
TMEM30B	*SLCO5A1*		
TMEM37	*SLCO6A1*		
TMEM40	*SLFN5*		
TMEM41A	*SLIT1*		
TMEM51	*SLIT2*		

TMEM55A	*SLITRK6*		
TMEM56	*SLK*		
TMEM66	*SLMAP*		
TMEM80	*SLURP1*		
TMEM85	*SMARCA4*		
TMEM90A	*SMARCC1*		
TMOD1	*SMARCD2*		
TMSB4X	*SMC1B*		
TMTC3	*SMC6*		
TNFAIP1	*SMCP*		
TNFRSF13C	*SMG5*		
TNFRSF19	*SMPD1*		
TNFRSF21	*SMUG1*		
TNFRSF22	*SMYD5*		
TNFSF10	*SNAI1*		
TNFSF11	*SNAP29*		
TNNC2	*SNAP91*		
TNNT3	*SNAPIN*		
TNPO2	*SNCA*		
TNRC4	*SNCG*		
TOM1L1	*SNED1*		
TOMM34	*SNF1LK*		
TOMT	*SNRK*		
TOP3A	*SNRPC*		
TOR1AIP1	*SNRPD1*		
TOX4	*SNRPD3*		
TPD52L1	*SNRPE*		
TPM2	*SNX1*		
TPO	*SNX10*		
TPPP3	*SNX12*		
TPRGL	*SNX13*		
TPRKB	*SNX16*		
TPSB2	*SNX2*		
TRAK2	*SNX20*		
TREX1	*SNX21*		
TRHR2	*SNX25*		
TRIM11	*SNX31*		
TRIM16	*SNX33*		
TRIM31	*SNX9*		
TRIM37	*SOAT2*		
TRIM6	*SOCS1*		
TRIML1	*SOCS3*		
TRIT1	*SORBS2*		
TRO	*SOS1*		
TRP53INP2	*SOX15*		
TRUB1	*SOX6*		

TSEN54	*SOX9*		
TSHB	*SP140*		
TSHR	*SP5*		
TSN	*SPA17*		
TSP50	*SPAG7*		
TSPAN10	*SPATA16*		
TSPAN11	*SPATA7*		
TSPAN14	*SPATS2*		
TSPAN17	*SPEF2*		
TSPYL1	*SPEN*		
TSPYL2	*SPERT*		
TSPYL4	*SPHKAP*		
TSR1	*SPINLW1*		
TSSK1	*SPO11*		
TSTA3	*SPON1*		
TTC12	*SPON2*		
TTC19	*SPRN*		
TTC21B	*SPRYD3*		
TTC39B	*SPSB1*		
TTLL6	*SPSB3*		
TTPA	*SPTBN2*		
TTR	*SPTBN4*		
TTYH1	*SPTLC3*		
TTYH2	*SQSTM1*		
TUBA1A	*SR140*		
TUBA1C	*SRC*		
TUBA3B	*SRFBP1*		
TUBA4A	*SRGAP1*		
TUBB3	*SRGAP3*		
TUBGCP5	*SRP54*		
TULP2	*SRP72*		
TUT1	*SRPRB*		
TXN1	*SRPX2*		
TXNDC12	*SS18L2*		
TXNDC13	*SSR3*		
TXNDC16	*SSTR1*		
TXNDC4	*SSU72*		
U2AF1	*SSX5*		
UACA	*ST13*		
UBAC1	*ST18*		
UBE1Y1	*ST3GAL3*		
UBE2A	*ST6GALNAC2*		
UBE2T	*STAB2*		
UBE2U	*STAG3*		
UBFD1	*STAMBP*		
UBL4B	*STAP1*		

UBN1	STAR		
UBR2	STARD3		
UBR7	STATH		
UBTD1	STAU1		
UBXN1	STC1		
UCHL5IP	STEAP1		
UCKL1	STEAP3		
UFM1	STK10		
UGCG	STK17B		
UGP2	STK19		
UGT2B34	STK3		
UGT3A1	STK33		
UHMK1	STK35		
UHRF1	STK38		
UHRF1BP1	STK4		
ULK1	STMN3		
UMOD	STOX2		
UNC119B	STRA13		
UNC93B1	STRAP		
UNG	STX18		
UNK	STX2		
UNKL	STX4		
UPP1	STX5		
UQCRC1	STXBP1		
URM1	SUGT1		
UROC1	SULT1C2		
UROS	SUMF2		
USH1G	SUOX		
USP12	SURF1		
USP14	SURF4		
USP32	SUSD1		
USP37	SUSD3		
USP43	SV2A		
USP48	SVIP		
USP6NL	SYF2		
UTP15	SYMPK		
UTS2	SYN2		
UVRAG	SYNC1		
UXT	SYNCRIP		
V1RD6	SYNE1		
VAMP8	SYNGR2		
VARS	SYNGR3		
VAT1	SYNJ1		
VEGFC	SYNJ2		
VIP	SYNPO2L		
VIPR2	SYT1		

VIT	*SYT12*		
VKORC1L1	*SYT17*		
VPS24	*SYT8*		
VPS28	*SYVN1*		
VPS33A	*TAC4*		
VPS33B	*TACC2*		
VPS39	*TACC3*		
VPS41	*TAF7L*		
VPS4A	*TAL2*		
VPS54	*TANK*		
VRK1	*TARBP1*		
VRK3	*TASP1*		
VSX2	*TATDN2*		
VTI1B	*TATDN3*		
VWA2	*TBC1D1*		
VWA5B2	*TBC1D10A*		
WAP	*TBC1D10B*		
WAPAL	*TBC1D12*		
WARS2	*TBC1D15*		
WAS	*TBC1D19*		
WBP11	*TBCE*		
WBSCR22	*TBKBP1*		
WBSCR27	*TBL2*		
WDR20B	*TBP*		
WDR32	*TBX5*		
WDR42A	*TCF25*		
WDR45	*TCF7L2*		
WDR59	*TCFL5*		
WDR68	*TCP1*		
WDR75	*TCTE1*		
WDR82	*TCTE3*		
WDR91	*TCTN3*		
WDR92	*TDRD6*		
WDR93	*TDRD7*		
WDTC1	*TEKT4*		
WEE1	*TENC1*		
WHSC2	*TEX2*		
WNT11	*TFAP2B*		
WNT16	*TFAP2C*		
WNT8A	*TFB2M*		
WRNIP1	*TFCP2L1*		
WSB2	*TFG*		
WSCD1	*TGFBI*		
WSCD2	*TGIF2*		
WWP2	*TH1L*		
XRCC5	*THAP7*		

XYLB	*THBD*		
YIPF5	*THEM2*		
YWHAB	*THNSL1*		
ZBED3	*THOP1*		
ZBED4	*THRAP3*		
ZBP1	*THSD3*		
ZBTB1	*THUMPD1*		
ZBTB45	*THUMPD3*		
ZBTB7B	*THYN1*		
ZBTB8OS	*TICAM1*		
ZC3H3	*TIFAB*		
ZC3HC1	*TIGD1*		
ZCCHC17	*TIGD5*		
ZCCHC3	*TIMM17B*		
ZCCHC6	*TIPIN*		
ZCCHC9	*TJAP1*		
ZDHHC11	*TJP2*		
ZDHHC16	*TK2*		
ZDHHC20	*TKTL1*		
ZER1	*TKTL2*		
ZFAND2B	*TLX2*		
ZFAND3	*TM4SF20*		
ZFHX4	*TM6SF1*		
ZFP106	*TM6SF2*		
ZFP109	*TM7SF4*		
ZFP110	*TM9SF3*		
ZFP148	*TM9SF4*		
ZFP157	*TMC8*		
ZFP161	*TMCC1*		
ZFP217	*TMCC3*		
ZFP238	*TMED7*		
ZFP281	*TMEFF1*		
ZFP316	*TMEM102*		
ZFP330	*TMEM103*		
ZFP341	*TMEM121*		
ZFP352	*TMEM127*		
ZFP362	*TMEM128*		
ZFP365	*TMEM141*		
ZFP36L1	*TMEM151A*		
ZFP385C	*TMEM165*		
ZFP418	*TMEM16F*		
ZFP438	*TMEM170*		
ZFP456	*TMEM173*		
ZFP498	*TMEM174*		
ZFP512	*TMEM176B*		
ZFP560	*TMEM177*		

ZFP568	*TMEM184B*		
ZFP575	*TMEM187*		
ZFP595	*TMEM205*		
ZFP598	*TMEM22*		
ZFP608	*TMEM43*		
ZFP619	*TMEM54*		
ZFP644	*TMEM55A*		
ZFP664	*TMEM59L*		
ZFP68	*TMEM62*		
ZFP691	*TMEM80*		
ZFP7	*TMEM86B*		
ZFP715	*TMEM97*		
ZFP748	*TMF1*		
ZFP770	*TMPRSS4*		
ZFP777	*TMUB1*		
ZFP784	*TNFAIP1*		
ZFP809	*TNFAIP6*		
ZFP828	*TNFRSF10B*		
ZFP830	*TNFRSF10C*		
ZFP91	*TNFRSF10D*		
ZFP94	*TNFRSF13B*		
ZFYVE1	*TNFRSF14*		
ZFYVE20	*TNFRSF6B*		
ZMAT3	*TNFSF10*		
ZNHIT3	*TNFSF13B*		
ZNRF1	*TNIP2*		
ZRANB3	*TNKS2*		
ZUFSP	*TNN*		
ZW10	*TNNC2*		
ZXDB	*TNRC4*		
ZYG11B	*TNRC6C*		
ZYX	*TNXB*		
	TOM1		
	TOMM20		
	TOMM34		
	TOR1AIP1		
	TPCN1		
	TPP2		
	TPPP2		
	TPRG1L		
	TRADD		
	TRAF1		
	TRAF3IP1		
	TRAF5		
	TRAF6		
	TRAM2		

	TRAPPC2L		
	TRAPPC6B		
	TRDN		
	TRIM10		
	TRIM21		
	TRIM32		
	TRIM41		
	TRIM44		
	TRIM46		
	TRIM5		
	TRIM56		
	TRIM6		
	TRIOBP		
	TRIP11		
	TRIP6		
	TRMT1		
	TRMT5		
	TRMT6		
	TRMU		
	TRO		
	TRPA1		
	TRPM7		
	TRPV4		
	TRSPAP1		
	TSC1		
	TSC2		
	TSFM		
	TSGA13		
	TSHB		
	TSKU		
	TSLP		
	TSN		
	TSNARE1		
	TSP50		
	TSPYL2		
	TSPYL4		
	TSR2		
	TSSC4		
	TSSK6		
	TTC1		
	TTC23		
	TTC26		
	TTC29		
	TTC31		
	TTC5		
	TTC8		

	TTC9C		
	TTF2		
	TTLL12		
	TTLL4		
	TTLL5		
	TTLL7		
	TUBA1A		
	TUBB2A		
	TUBB6		
	TUBE1		
	TULP3		
	TUSC3		
	TUT1		
	TWF2		
	TXLNA		
	TXN2		
	TXNDC12		
	TXNDC13		
	TXNDC4		
	U1SNRNPBP		
	UACA		
	UAP1L1		
	UBA1		
	UBA52		
	UBAC1		
	UBAP1		
	UBASH3A		
	UBC		
	UBE2G2		
	UBE2I		
	UBN1		
	UBQLNL		
	UCN2		
	UCP2		
	UCP3		
	UGP2		
	UGT1A6		
	UGT1A8		
	UGT2A3		
	UHRF1BP1		
	ULBP1		
	UMOD		
	UNC119B		
	UNC13B		
	UNC45B		
	UNC93B1		

	UNK		
	UPK1A		
	UQCRC1		
	USF1		
	USP13		
	USP14		
	USP31		
	USP32		
	USP4		
	USP44		
	USP46		
	USP48		
	USP5		
	USP6		
	USP7		
	USP8		
	USP9Y		
	UTF1		
	UTP15		
	UTS2D		
	VAMP3		
	VAMP8		
	VARS		
	VAT1		
	VCL		
	VGLL3		
	VGLL4		
	VIT		
	VKORC1		
	VPRBP		
	VPS24		
	VPS29		
	VPS33B		
	VPS37A		
	VPS39		
	VPS8		
	VSIG1		
	VSIG4		
	VTI1B		
	VWA3B		
	VWF		
	WARS2		
	WBP11		
	WDFY4		
	WDR13		
	WDR26		

	WDR31		
	WDR33		
	WDR37		
	WDR47		
	WDR59		
	WDR6		
	WDR62		
	WDR81		
	WDR85		
	WDR91		
	WDR92		
	WDSOF1		
	WDTC1		
	WFDC1		
	WFDC10A		
	WFDC12		
	WFDC5		
	WFDC8		
	WHSC1L1		
	WIBG		
	WIPF1		
	WISP1		
	WNT4		
	WNT8A		
	WNT9B		
	WRNIP1		
	WSCD2		
	WWC1		
	XBP1		
	XPO4		
	XPO5		
	XPO6		
	XYLB		
	XYLT1		
	YDJC		
	YIF1A		
	YIF1B		
	YPEL3		
	YPEL5		
	YTHDC1		
	YTHDC2		
	YWHAB		
	YWHAZ		
	ZBTB25		
	ZBTB46		
	ZBTB5		

	ZBTB7A		
	ZBTB7B		
	ZC3H11A		
	ZC3H12A		
	ZC3H3		
	ZC3H7A		
	ZCCHC2		
	ZCCHC9		
	ZDHHC16		
	ZDHHC17		
	ZDHHC19		
	ZDHHC22		
	ZDHHC3		
	ZDHHC6		
	ZDHHC7		
	ZFHX3		
	ZFHX4		
	ZFP1		
	ZFP28		
	ZFP30		
	ZFP36L1		
	ZFP91		
	ZFYVE16		
	ZFYVE26		
	ZFYVE9		
	ZIC1		
	ZIC5		
	ZIK1		
	ZIM2		
	ZMYM4		
	ZNF114		
	ZNF124		
	ZNF132		
	ZNF135		
	ZNF148		
	ZNF16		
	ZNF169		
	ZNF175		
	ZNF187		
	ZNF193		
	ZNF20		
	ZNF202		
	ZNF211		
	ZNF224		
	ZNF233		
	ZNF238		

	ZNF264		
	ZNF274		
	ZNF275		
	ZNF277		
	ZNF280D		
	ZNF281		
	ZNF285A		
	ZNF317		
	ZNF346		
	ZNF35		
	ZNF358		
	ZNF362		
	ZNF385D		
	ZNF474		
	ZNF498		
	ZNF544		
	ZNF546		
	ZNF566		
	ZNF570		
	ZNF573		
	ZNF594		
	ZNF598		
	ZNF608		
	ZNF622		
	ZNF628		
	ZNF689		
	ZNF691		
	ZNF695		
	ZNF701		
	ZNF711		
	ZNF746		
	ZNF766		
	ZNF789		
	ZNF83		
	ZNF831		
	ZNF84		
	ZNHIT3		
	ZNRF1		
	ZNRF3		
	ZPLD1		
	ZRANB1		
	ZSCAN1		
	ZSWIM3		
	ZSWIM7		
	ZYX		
	ZZEF1		
